# The impact of environmental exposures on DNA methylation in the EXPANSE project

**DOI:** 10.1016/j.ebiom.2025.106084

**Published:** 2025-12-19

**Authors:** Megi Vogli, Ayoung Jeong, Zhebin Yu, Judith M. Vonk, Dorina Ibi, Jaanika Kronberg, Petr Gregor, Lisa Maier, Miriam Leskien, Marta Cirach, Payam Dadvand, Ondřej Mikeš, Olena Gruzieva, Ulrike Gehring, Kathrin Wolf, Melanie Waldenberger, Medea Imboden, Pavel Čupr, Kees de Hoogh, Gerard H. Koppelman, Erik Melén, Regina Pickford, Elisabeth Thiering, Marie Standl, Estonian Biobank Research Team, Apolline Saucy, Cathryn Tonne, Cristina O'Callaghan, Manolis Kogevinas, Mark Nieuwenhuijsen, Marta Cirach, Natalia Ortega, Payam Dadvand, Sergio Olmos, Erik Melén, Douglas Walker, Joline Beulens, Maria Gabriela Matias Pinho, Joreintje Mackenbach, Licia Iacoviello, Daniela Porta, Federica Asta, Martina Culasso, Massimo Stafoggia, Ardine de Wit, Tabea Sonnenschein, Simon Scheider, Karin Jongsma, Annelien Bredenoord, Caspar Safarlou, Anna Oudin, Bertil Forsberg, David Olsson, Matteo Bottai, Craig Wheelock, Jaanika Kronberg, Tarmo Annilo, Tõnu Esko, René Luigies, Rob Tieben, Anna Carreras, Rafael de Cid, Beatriz Cortés, Mireia Obon, Barbara Bodinier, Dragana Vuckovic, Dusan Petrovic, Jennifer Quint, Marc Chadeau-Hyam, Matthew Whitaker, Paolo Vineis, Sarah Filippi, Sonia Dagninio, Thomas Wright, Verena Zuber, Anna Bergström, Göran Pershagen, Olena Gruzieva, Petter Ljungman, Shizhen He, Hynek Pikhart, Martin Bobak, Alexandra Schneider, Annette Peters, Kathrin Wolf, Marie Standl, Regina Pickford, Susanne Breitner, Tianyu Zhao, Melanie Waldenberger, Augustin Scalbert, Inge Huybrechts, Marc Gunter, Pekka Keski-Rahkonen, Reza Salek, Jana Klánová, Lenka Andrýsková, Ondřej Mikeš, Pavel Čupr, Pavel Piler, Richard Hůlek, Zdenka Dudová, Nicole Janssen, Alonso Bussalleu, Ayoung Jeong, Benjamin Flückiger, Danielle Vienneau, Dirk Keidel, Emmanuel Schaffner, Gianfranco Lovison, Ikenna Eze, Kees de Hoogh, Marek Kwiatkowski, Medea Imboden, Nicole Probst-Hensch, Cyrille Delpierre, Marine Maurel, Michelle Kelly-Irving, Raphaële Castagné, Benoit Lepage, Evangelia Samoli, Klea Katsouyanni, Konstantina Dimakopoulou, Sophia Rodopoulou, Maria Iosifina Kasdagli, Dimitris Evangelopoulos, Anke Huss, Esmeralda Krop, Gerard Hoek, Jelle Vlaanderen, Jingxian You, Jules Kerckhoffs, Kalliopi Kyriakou, Lützen Portengen, Martje Ebberink, Roel Vermeulen, Ulrike Gehring, You-chen Shen, Zhendong Yuan, Jeroen Lakerveld, Alessandro Gialluisi, Gary Miller, Jurriaan van Rijswijk, Simona Costanzo, Werner Rutten, Marta Mańczuk, Paweł Koczkodaj, Agata Ciuba, Kinga Polańska, Wojciech Hanke, Agnieszka Jankowska, Agnieszka Pac, Elzbieta Sochacka-Tatara, Renata Majewska, Jana Klánová, Jelle Vlaanderen, Roel Vermeulen, Nicole Probst-Hensch, Annette Peters, EXPANSE Project

**Affiliations:** a Institute of Epidemiology, Helmholtz Zentrum München, German Research Center for Environmental Health, 85764, Neuherberg, Germany; b Swiss Tropical and Public Health Institute, Allschwil, Switzerland; c Department of Public Health, University of Basel, Basel, Switzerland; d Institute of Environmental Medicine, Karolinska Institute, Stockholm, Sweden; e Department of Epidemiology, University of Groningen, University Medical Center Groningen, Groningen, the Netherlands; f Institute for Risk Assessment Sciences, Utrecht University, the Netherlands; g Estonian Genome Centre, Institute of Genomics, University of Tartu, Tartu, Estonia; h RECETOX, Faculty of Science, Masaryk University, Brno, Czechia; i Ludwig-Maximilians-Universität Munich, Institute for Medical Information Processing, Munich, Germany; j ISGlobal, Barcelona, Spain; k Universitat Pompeu Fabra (UPF), Barcelona, Spain; l CIBER Epidemiología y Salud Pública (CIBERESP), Madrid, Spain; m Department of Pediatric Pulmonology and Pediatric Allergology, Beatrix Children's Hospital, University Medical Center Groningen Groningen, the Netherlands; n Groningen Research Institute for Asthma and COPD, University of Groningen, University Medical Center Groningen, Groningen, the Netherlands; o Department of Clinical Science and Education Södersjukhuset, Karolinska Institute and Sachs' Children and Youth Hospital, Stockholm, Sweden; p German Center for Lung Research (DZL), Munich, Germany; q German Center for Child and Adolescent Health (DZKJ), Partner Site Munich, Munich, Germany

**Keywords:** DNA methylation, Exposome, External exposome, Internal exposome, Life course assessment, European cohorts

## Abstract

**Background:**

Living in an urban environment exposes the population to a mix of environmental and social factors, known as the Urban Exposome, that can influence health via changes in DNA methylation. We hypothesised that linking urban exposures with epigenome-wide DNA methylation in blood can reveal impacts across the lifespan.

**Methods:**

In the EXPANSE project, we conducted an inverse variance-weighted meta-analysis of epigenome-wide association studies of seven European cohorts. Urban exposures were estimated at participants' home addresses and included air pollution (PM2.5, NO_2_, O_3_), light at night, modified soil-adjusted vegetation index, and urbanicity.

**Findings:**

DNA methylation was measured in blood samples from 1778 children (4–10 years), 878 adolescents (16 years), and 5975 adults (18–87 years). PM2.5, NO_2_, and greenness were associated with methylation differences in children, while greenness and urbanicity showed associations in adults. Regional analyses showed differentially methylated regions (DMRs) across all life stages. Pathway analysis showed that monthly NO_2_ in children was linked to immune and infectious disease pathways, whereas adult urbanicity was associated with immune pathways as well as PD-L1 expression and the PD-1 checkpoint pathway in cancer.

**Interpretation:**

Urban environmental factors induce DNA methylation changes across life stages, with stronger associations in young children and adults. We observed a distinct contrast in the methylation changes associated with greenness compared to other urban environmental factors. However, disentangling exposure-specific methylome signatures remains a challenge.

**Funding:**

This work was supported by the EXPANSE project, funded by the 10.13039/501100007601European Union’s Horizon 2020 research and innovation programme under grant agreement No. 874627.

**Research in context**

**Evidence before this study**

Previous epigenome-wide association studies have investigated the effects of environmental exposures on DNA methylation. However, most of the available evidence derives from analyses focussing on a single exposure at a specific point in life. We searched PubMed and Web of Science for peer-reviewed studies using the search terms (“Methylation” AND “Environment”), (“Methylation” AND “Environment” AND “Exposome”), and (“Methylation” AND “Environment” AND “Life course”), from inception to November 2025. Our search confirmed that existing studies have primarily examined specific environmental exposures during a particular life stage and targeted single outcomes, rather than providing a broader, integrative overview.

**Added value of this study**

This study analysed data from seven cohorts across Europe, spanning four different life stages, from early childhood to adulthood. Uniformly estimated environmental exposures were linked in relation to DNA methylation measured at each stage. A combination of statistical and bioinformatical approaches was applied to evaluate associations across exposures and life stages, enabling both cross-exposure comparisons and the identification of potential life-stage-specific effects. This approach provides a comprehensive view of how different environmental factors shape the epigenome across the life course.

**Implications of all the available evidence**

Our findings reinforce the evidence that environmental factors influence DNA methylation and suggest that the involvement of specific genes varies across life stages. This contributes to a better understanding of the biological mechanisms underlying environmental impacts on health. Moreover, the observation that greenness may exert distinct effects compared with urban-related environmental exposures has potential implications for public health strategies and urban planning, informing future decisions to promote healthier cities.

## Introduction

Given ongoing urbanisation worldwide, understanding the impact of urban living on human health is one of the major challenges of our time. The urban exposome is a broad concept that includes the cumulative environmental exposures that individuals encounter daily, from the physic-chemical environment (such as air and water pollution, temperature fluctuations, and chemical exposures) to the built environment (including green and blue spaces and urban density). Together, these factors can have both beneficial and harmful effects on health, but so far, the mechanisms underlying these effects remain only partly understood. The interactions between environmental exposures and the molecular processes that drive cellular function have long been a focus of research. Molecular biology traditionally centres on the central dogma, the flow of genetic information from DNA to RNA to protein.^1^ However, this process can be modulated by the environment at multiple levels, including at the epigenetic level.^2^^,^^3^

Epigenetic modifications are essential for normal cell development and function. In this work, we focused on DNA methylation, which consist of the attachment of a methyl group (CH_3_) to the 5th carbon of a cytosine nucleotide, usually followed by a guanine nucleotide, known as CpG sites.^4^ DNA methylation is a dynamic process that can influence gene expression by regulating the binding of methylation-sensitive transcription factors.^5^ Changes in blood DNA methylation profiles may results from exposome induced alterations in cell type composition, as well as the activation or suppression of specific white blood cells, reflecting immune system modulation and inflammatory responses.^6^ Previous studies have demonstrated that exposure to ambient air pollutants can alter blood DNA methylation at several gene loci.^7^^,^^8^ Additionally, residential greenness has been associated with blood DNA methylation profiles enriched in allergy, physical activity and allostatic load.^9^^,^^10^ However, important gaps remain: most studies have focused on single exposures at a specific life stage, and evidence across different life stages is still scarce.

In this study, we examined how the exposure to urban environmental factors, modelled for individuals’ home addresses as part of the EXPANSE project,^11^ associates with blood DNA methylation levels across different life stages in various European cohort studies. In our analyses, we characterised the urban exposome by fine particulate matter (PM2.5), nitrogen dioxide (NO_2_) (both monthly and annual averages), ozone (O_3_ warm season), light at night (LAN), the modified soil-adjusted vegetation index (MSAVI), and urbanicity. By grouping the cohort studies by life stage, we aimed to determine^1^ whether the relationship between environmental factors and DNA methylation differs across life stages and^2^ whether these effects are specific to individual exposures or if there is an overlap among different environmental factors.

## Methods

### Participants

All cohorts participating in the EXPANSE project with available DNA methylation data were included in the meta-analysis. EXPANSE^11^ is a 5-year European project aiming to improve the quality of health in urban environments (https://expanseproject.eu/). In total, six cohorts from central and northern Europe and one from Eastern Europe, covering multiple time points, were included in the meta-analysis. The studies were grouped by life stage into the following groups: children aged 4–6 years, children aged 8–10 years, adolescents aged 16 years, and adults aged over 18 years. The PIAMA (Prevention and Incidence of Asthma and Mite Allergy) study, an ongoing cohort from the Netherlands, contributed data from participants aged 4, 8, and 16 years collected between 2000 and 2014.^12^ Similarly, the BAMSE (Children, Allergy, Milieu, Stockholm, Epidemiology) study from Sweden provided data for participants at the same ages (4, 8, and 16 years), collected between 1998 and 2013.^13^ The LISA (Lifestyle Influence of Life-style factors on the development of the Immune System and Allergies in East and West Germany) study from Germany contributed data for participants aged 6 and 10 years, collected between 2004 and 2009.^14^ For the adult group, the NTR (Netherlands Twin Register) study from the Netherlands provided data collected between 2004 and 2008^15^ and the Estonian Biobank contributed with data from 2002 to 2012.^16^^,^^17^ The SAPALDIA (Study on Air Pollution and Lung Disease in Adults) cohort from Switzerland provided data collected between 2010 and 2011.^18^^,^^19^ Additionally, the KORA (Cooperative Health Research in the Region of Augsburg) study from southern Germany, contributed with data from the F4 follow-up period (2006–2008).^20^ The CELSPAC: YA (Central European Longitudinal Studies of Parents and Children: Young Adults) cohort provided saliva samples data from young adults, which were included in this work for a comparative analysis. Further details about this cohort study and the other participating cohorts are provided in the Supplementary Material.

### External exposome data

Europe-wide environmental exposure data were modelled at each participant's home address following the harmonised protocol within the EXPANSE project,^11^ using data collected between 2000 and 2019. All exposures were estimated using land-use regression models (LUR). These are statistical models commonly used in environmental epidemiology to estimate and explain the spatial variability of long-term outdoor air pollution. The models rely on predictors variables from Geographic Information System (GIS),^21^ such as traffic density, and proximity to industrial sources, population density, and land-use characteristics.

#### Physical-chemical environment

Exposure data included monthly and annual average concentrations of PM2.5, NO_2_, and ozone (O_3_), with detailed descriptions of data collection and modelling procedures reported elsewhere.^22^^,^^23^ For PM2.5, daily measurements were aggregated to derive the monthly and annual averages. For NO_2_, hourly measurements were aggregated into both monthly and annual averages. For O_3_, a daily maximum 8-h mean was computed for each day and subsequently aggregated into monthly and annual averages. Land-use regression (LUR) was built for each year from 2000 to 2019 for PM2.5 NO_2_ and O_3_. Annual PM2.5 and NO_2_ concentrations were linked to the participants data based on the mean value for the same year as the blood sample collection. Monthly PM2.5 and NO_2_ concentrations were linked based on the mean value of the month preceding blood sampling. For O_3_, the warm-season average (April–September) was used as an indicator of annual exposure.

#### Built environment exposures

Built environment exposures included LAN, MSAVI as a proxy for greenness, and urbanicity. LAN and MSAVI were assessed within a 500 m buffer of the home address (determined via GIS-based distance calculations), with a spatial resolution of 250 × 250 m across five different time periods (2000–2020). The exposure value for each participant was assigned based on the year closest to their examination date.

LAN data were assessed from harmonised data produced with the Defence Meteorological Satellite Program (DMSP) and Version 1 VIIRS Day/Night Band Night-time Lights.^24^ LAN intensity was represented by an integer scale from 0 to 63, where higher values indicate greater light intensity. MSAVI was calculated using reflectance data from the MOD13Q1 product of the Terra satellite, specifically the RED and near infrared band bands (NIR).^25^

Urbanicity, was defined as population density, calculated using a focal mean within a 1500 m square, with a spatial resolution of 100 × 100 m.^26^ As urbanicity data were only available for 2015, these values were linked to all cohorts, assuming minimal temporal variation in population density across Europe during the time.

### DNA methylation data

DNA methylation was measured in blood samples of the study participants from the different cohorts. The methylation level across the cohorts was measured using either the Infinium HumanMethylation450 K Bead Chip or the MethylationEPIC BeadChip platform (Illumina, Inc., San Diego, CA). Each cohort conducted the quality control of the methylation data by using their pre-processing and normalisation pipelines, details are given in the Supplementary Material. Transformed M-values were used for epigenome-wide association study (EWAS) analyses.^27^

### Statistics

#### Cohorts specific–EWAS

For the analyses, each participant was assigned the exposure value from the time point closest to their examination date. For instance, for LAN and MSAVI, if a participant was examined in 2006, the exposure was derived from the 2005. Each cohort conducted multiple linear regression analyses, with methylation data as the outcome and each available exposure as the predictor (per 1-unit increase) adjusting for confounders. The only exception is the NTR study, which represents a random sample of Dutch families with twins; to account for this, they used a generalised estimating equations (GEE) model (Supplementary Material). The potential influence of technical factors was accounted for by including principal components (PCs) derived from negative control probes as covariates in the model. Specifically, cohorts could either regress M-values on the first PCs and use the residuals as DNA methylation values in the model^28^ or include the PCs directly as covariates. A single model was applied, adjusting for key covariates such as age, sex, and smoking behaviour. Sex (male/female) was recorded at recruitment by questionnaire or registry data in each cohort. In mature birth cohorts, environmental tobacco smoke (ETS) exposure was categorised as yes/no, whereas in adult cohorts, smoking status was classified as smoker, ex-smoker, or non-smoker. Furthermore, the model accounted for cell-type composition to ensure that observed associations reflected exposure-related methylation changes rather than variations in leucocyte composition. In the Supplementary Material, the estimate cell counts methods are reported for each cohort. Additionally, cohorts included study-specific variables for adjustment as needed. EWAS results were received for all available environmental factors from all studies.

#### Quality control and meta-analysis

Quality control (QC) of cohort-specific EWAS results followed the QCEWAS (v1.2.3) R package approach.^29^ Results with genome inflation (ʎ) > 1.3 or < 0.8 were considered inflated, and therefore, Bacon's correction^30^ was applied. To ensure consistency across all studies, Bacon's correction was applied to all cohort-specific EWAS results. Exception was made for EWAS results of LISA 6 years with NO_2_ annual and O_3,_ were correction further inflated the results. If present, probes targeting sex chromosomes were removed. Outlier values, defined as those below Q1–3 × interquartile range (IQR) or above Q3 + 3 × IQR, were removed during QC of EWAS cohort-specific results.

A two-stage meta-analysis was performed using an inverse-variance weighted, fixed effect model in METAL,^31^ separately for each exposure for the four life stages: children aged 4–6 years, children aged 8–10 years, adolescents (16 years), and adults (18 years or older). Meta-analyses were conducted only on common probes, and inflation factor was checked for all exposures. The Benjamini-Hochberg procedure was used to control the False Discovery Rate (FDR), and probes with FDR <10% were considered significant. Results with substantial inter-cohort heterogeneity (*I*^*2*^ > 70% and/or Q-test p ≤ 0.05) were excluded ensuring that the reported associations were not driven by inconsistent effects across studies. Probe annotations were obtained from the Illumina Infinium HumanMethylation450 BeadChip manifest file (Illumina, Inc., San Diego, CA).^32^ To account for geographical variation, for the adult age group, a sensitivity analysis was conducted with exclusion of Estonian BioBank, located in Eastern Europe. Lastly, all the results derived from blood samples were compared with those from saliva samples to investigate potential concordance.

#### Intersection analysis

The results were analysed by life stage to identify potential common hits across exposures within each life stage group. A suggestive significance threshold of p-value <1 × 10^−5^ was applied to select significant findings. The significant results were then intersected across exposures to identify overlapping hits within each age group. Additionally, we examined consistency by intersecting results for each exposure across age groups.

#### Differentially methylated regions analysis

Differentially methylated regions (DMRs) analysis was conducted using DMRcate (v2.16.1) R package,^33^ and results were compared to those obtained with the ipDMR (v1.38.1) method.^34^ The two approaches rely on different statistical methods. DMRcate identifies DMRs by applying Gaussian kernel smoothing to the differential methylation signal across the genome.^33^ In contrast, ipDMR identifies DMRs by leveraging auto-correlated p-values from individual CpGs obtained through EWAS analysis.^34^ For the DMRcate analysis, parameters were set as follows: lambda (smoothing bandwidth) = 1000, min.cpgs (minimum CpGs per region) = 5, and C (scaling factor for test statistic) = 2. For the ipDMR analysis, parameters were set as dist.cutoff (maximum distance between CpGs to be grouped, analogous to lambda in DMRcate) = 1000. Notably, ipDMR can yield significant regions that consist of only one CpG, whereas DMRcate was configured to require a minimum of five CpGs per region. This difference was taken into consideration when comparing the results from the two methods.

In the results obtained with DMRcate, we filtered the data to retain only those DMRs that were common across the different exposures within each life stage group. To determine the direction of methylation changes, we used the parameter “mean differences” (meandiff), which calculates the average difference in methylation levels across all CpGs within a given DMR. In general, a positive meandiff value indicates an average increase in methylation, whereas a negative value indicates an average decrease.^33^ Probe annotations were obtained from the Illumina Infinium HumanMethylation450 BeadChip manifest file (Illumina, Inc., San Diego, CA).^32^

#### Integrative expression quantitative trait methylation (eQTM) analysis

To explore the potential relationship between CpGs methylation level and nearby gene expression we cross-referenced the CpGs resulted from the meta-analysis with the BIOS QTL public repository (BIOS QTL browser). This resource provides 18,881 significant cis-eQTM associations (FDR <0.05) derived from whole blood expression, based on DNA methylation (Illumina 450 K array) and RNA-seq data.^35^ First, CpGs results that have met a suggestive significance threshold (p-value <1 × 10^−5^) in the meta-analysis were compared in BIOS. Second, all CpGs included within the DMRs identified by with DMRcate were mapped to help with the biological interpretation of regional methylation effect potentially reflecting alterations in gene expression and thereby increasing clinical relevance.

#### Pathway enrichment analysis

The primary pathway enrichment analysis was conducted on all CpGs that were part of significant DMRs identified by DMRcate. All identified CpGs were mapped to genes using BIOS dataset and included in the pathway analysis. Pathway enrichment analysis was performed using the Enrichr (v3.4) R package^36^ applying the enrichr function with the curated databases *GO_Biological_Process_2023* and *KEGG_2021_Human*. Pathways with an adjusted p-value (Benjamini-Hochberg) < 0.05, and with more than two gene (count) from the input set per pathway were considered significant. Results were compared with those obtained using MissMethyl (v1.36.0) R package,^37^ which directly includes in the *gometh* function the CpGs sites, adjusting for the number of CpGs associated with each gene. Pathways with an adjusted p-value (Benjamini-Hochberg) < 0.05 were considered significantly enriched.

### Ethics

This meta-analysis was conducted using summary statistics from consortium-based epigenome-wide association studies. Each contributing cohort obtained ethical approval from its respective institutional review board and informed consent from all participants. No additional ethical approval was required for the present analysis. Acknowledgements and detailed ethical information for each contributing study are provided in the Supplementary Material.

### Role of the funders

The funders had no role in study design, data collection, data analysis, data interpretation, or writing of the report. The corresponding author had full access to all the data in the study and had final responsibility for the decision to submit for publication.

## Results

### Study population

This study involved participants from seven European cohorts of different ages (Table 1). Among them, 717 children aged 4–6 years, with a mean age of 4.8 (±0.9), and 1061 children aged 8–10 years, with mean age of 8.7 (±0.9). The adolescent group consisted of 878 participants, all aged 16 years, with a mean age of 16.4 (±0.3). The adult group included 5975 participants aged 18–87 years, with a mean age of 48.1 (±16.5). Overall, 55.4% of participants were female. Among the adult participants, 18.7% were smokers while of 48.3% were never non-smokers, and the remaining 31.5% ex-smokers. Among children and adolescents, 11.8% were exposed to ETS.

**Table 1:** Descriptive statistics of cohort studies included in the meta-analysis by life stage.

Life stage	N tot.	Cohorts	N	Age (Mean ± SD)	Female (%)	Male (%)	Current smoker (%)	Former smoker (%)	Never smoker (%)	ETS (%)
Adults	5975	KORA F4	1722	61.0 ± 8.9	882 (51.2)	840 (48.8)	248 (14.4)	753 (43.7)	721 (41.9)	–
		Sapaldia	969	58.8 ± 11.3	521 (53.8)	448 (46.2)	217 (22.4)	357 (36.8)	395 (40.8)	–
		EstonianBiobank	303	50.1 ± 16.9	151 (49.8)	152 (50.2)	56 (18.5)	93 (30.7)	154 (50.8)	–
		NTR	2981	36.9 ± 13.0	1900 (63.7)	1081 (36.3)	598 (20.7)	677 (23.4)	1616 (55.9)	–
Adolescents	878	PIAMA	611	16.3 ± 0.2	311 (50.9)	300 (49.1)	30 (4.9)	79 (12.9)	502 (82.2)	42 (6.9)
		BAMSE	267	16.7 ± 0.3	117 (43.8)	150 (56.2)	–	–	–	31 (11.6)
Children^8^, ^9^, ^10^	1061	LISA	226	10.2 ± 0.1	96 (42.5)	130 (57.5)	–	–	–	7 (3.2)
		PIAMA	205	8.1 ± 0.3	103 (50.2)	102 (49.8)	–	–	–	27 (13.2)
		BAMSE EpiGene	375	8.3 ± 0.7	172 (45.9)	203 (54.1)	–	–	–	63 (16.8)
		BAMSE MeDALL	264	8.4 ± 0.4	121 (45.8)	143 (54.2)	–	–	–	46 (17.4)
Children^4^, ^5^, ^6^	717	LISA	234	6.1 ± 0.2	99 (42.3)	135 (57.7)	–	–	–	15 (6.9)
		PIAMA	228	4.1 ± 0.2	122 (53.5)	106 (46.5)	–	–	–	38 (16.7)
		BAMSE	255	4.3 ± 0.3	117 (45.9)	138 (54.1)	–	–	–	44 (17.3)

N tot. = Total number of participants grouped by life stage; N = Number of participants within each study population; SD = standard deviation; ETS = Environmental Tabacco Exposure (yes).

### Exposure data

Descriptive statistics of exposure variables for each cohort are presented in Table S1. Exposure data were available for nearly all cohorts. Monthly averages of PM2.5, however, were not available for the years of the children's study age group (4–8 years), except for the LISA (10 years) cohort. Monthly NO_2_ data were unavailable for BAMSE (4 years), precluding its inclusion in the analysis. Overall, cohorts from geographically close areas exhibited similar pollutant levels (Figure S1). Estonian region, in particular, had lower levels of air pollutants compared to other regions. Urbanicity for the BAMSE cohort showed a considerable variation, indicated by a large standard deviation, likely due to the diversity in population density across different study areas.

### Meta-analysis of epigenome-wide association studies

All cohort-specific results that successfully passed the quality control were included in the meta-analysis. The inflation factor was checked for each EWAS for all exposures, and in general was for all around 1 (Table S2). The EWAS results from the LISA 6 years cohort with urbanicity were excluded from the meta-analysis due to high inflation factor value.

**Fig. 1:**
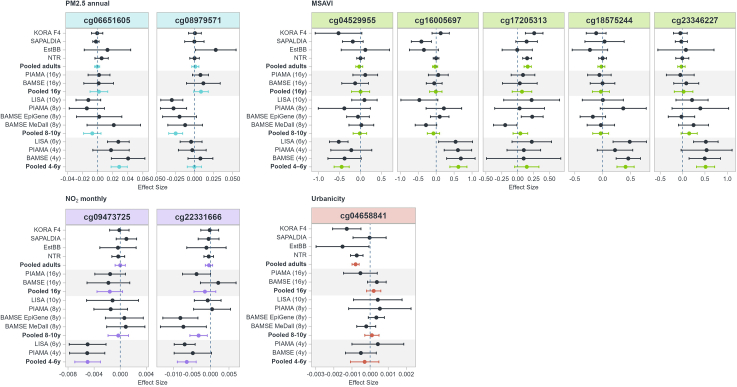
Effect estimates (95% CI) for significant DMPs identified in the meta-analysis, shown across all cohorts. Data for NO_2_ monthly were not available in BAMSE at age 4 years, and EWAS results for urbanicity from LISA at age 6 years were excluded from the meta-analysis during quality control. Results are grouped by exposure and ordered by the cohorts' average age, with pooled estimates also presented (*children 4–6 years: n = 717; children 8–10 years: n = 1061; adolescents: n = 878; adults: n = 5975*).

The main results are presented in Table 2. In children aged 4–6 years, annual PM2.5 average was associated with one differentially methylated position (DMP) (cg06651605), monthly NO_2_ with two DMPs (cg22331666, cg09473725) and MSAVI with four DMPs (cg16005697, cg18575244, cg04529955, cg23346227). In children aged 8–10 years annual PM2.5 was associated with one DMP (cg08979571). In adults, MSAVI was associated with one DMP (cg17205313), as was urbanicity (cg04658841). The findings varied across life stages and exposure types. Annotation details for all significant DMPs associated with the exposures are provided in Table 2. The trend of significant DMPs across all cohorts was examined to assess whether they varied among different age groups (Fig. 1). In general, there is a difference in the direction of the results by life stage, particularly between the children's cohorts at ages 4–6 years and the adult cohorts. Within each age group, in general, the direction of the estimates is consistent across studies. Within the adult age group, we conducted a sensitivity analysis excluding Estonian Biobank to account for potential geographic variation and results are presented in Table S3. The association with MSAVI remained unchanged, while annual NO_2_ was additionally associated with the same DMP (cg17205313) but in the opposite direction. A significant association was also observed for monthly PM2.5 with one DMP (cg10112003). In contrast, the association for Urbanicity was no longer significant. All DMPs identified in the meta-analysis were examined in the saliva sample results for comparison; the findings are reported in the Supplementary Material (Tables S4–S5, Figure S2).

**Table 2:** Summary of meta-analysis results for different exposures according to life stage.

Life stage	Exposure	Probe ID	β	StdErr	pvalue	FDR	Dir.	I^2^	CHR	Relation to CpG Island	Gene region	Mapped gene
Adult	MSAVI	cg17205313	0.157	0.029	5.53E-08	0.021	+−++	25.7	18	OpenSea	5′UTR; 1stExon	TTC39C
	Urbanicity	cg04658841	−0.001	0	2.37E-07	0.089	−−−−	43	16	OpenSea	–	–
Children 8-10	PM2.5 annual	cg08979571	−0.025	0.005	8.33E-08	0.03	−−−−	0	19	Island	Body	C2CD4C
Children 4-6	PM2.5 annual	cg06651605	0.029	0.006	2.44E-07	0.088	+++	0	5	OpenSea	–	–
	NO_2_ Monthly	cg22331666	−0.006	0.001	3.22E-07	0.077	−−	0	20	Island	TSS200	STX16
		cg09473725	−0.005	0.001	4.27E-07	0.077	−−−	0	1	OpenSea	Body	CD247
	MSAVI	cg16005697	0.62	0.121	3.34E-07	0.049	+++	0	11	OpenSea	–	–
		cg18575244	0.409	0.079	1.88E-07	0.049	+++	0	14	S_Shelf	3′UTR	UBR7
		cg04529955	−0.45	0.092	1.07E-06	0.077	−−−	0	4	N_Shore	Body	SLC10A7
		cg23346227	0.515	0.103	6.11E-07	0.055	+++	0	12	S_Shore	Body	BAZ2A

β = coefficient estimate. StdErr = standard error. FDR = False Discovery Rate 10% (threshold). Dir. = Direction, summary of the effect direction for each study. Description of the annotation columns all provided by Illumina^31^: CHR = chromosome number; Relation to CpG Island: the location of the CpG relative to the CpG island (Shore = 0–2 kb from island; Shelf = 2–4 kb from island; N = upstream (5′) of CpG island; S = downstream (3′) of CpG island); Gene region: (TSS200 = 0–200 bases upstream of the transcriptional start site (TSS); 5′UTR = Within the 5′ untranslated region, between the TSS and the ATG start site; 1stExon = within the first exon of the transcript; Body = Between the ATG and stop codon–irrespective of the presence of introns, exons, TSS, or promoters; 3′UTR = Between the stop codon and poly A signa); Mapped gene = reference genome the Illumina Infinium HumanMethylation450 BeadChip manifest file (Illumina, Inc., San Diego, CA).

### Exposures intersection analysis

**Fig. 2:**
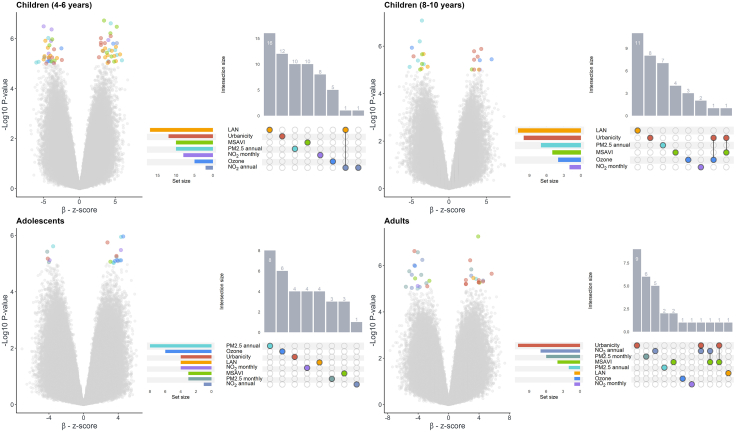
Volcano plot displaying differential methylation results across multiple exposures. Standardised estimates (β) are shown on the x-axis. CpG sites meeting the suggestive significance threshold (p-value <1 × 10^−5^) are colour-coded according to their associated exposures. The adjacent UpSet plot illustrates the unique intersections and unique sets of significant CpG sites across the exposures. Results are shown by age group *(children 4–6 years: n = 717; children 8–10 years: n = 1061; adolescents: n = 878; adults: n = 5975).*

To further explore our findings over the life course, we conducted an intersection analysis. First, we applied a suggestive significance threshold (p-value <1 × 10^−5^); results by life stage are provided in the Supplementary Material (Tables S6–S9), CpGs excluded due to high heterogeneity (*I*^*2*^ > 70%) are reported in Table S10, along with cis-eQTM analysis results (Table S11). Following this, we conducted an intersection analysis. The results are presented in Fig. 2, using both a volcano plot and an UpSet plot for visualisation. Among the life stages, in children 4–6 years, LAN exhibited the highest number DMPs-association, including one DMP (cg20428720) shared with annual NO_2_, where no annotated gene was reported. Similarly, children 8–10 years LAN showed the highest number of associated DMPs. Notable intersections were observed between urbanicity with O_3_ (cg09775260), and with MSAVI (cg08774231). The latter annotated to the *PPP2R2B* gene. In adolescents, annual PM2.5 showed the highest number of DMPs, but no intersections across the exposures were observed. Among adults, urbanicity showed the highest number of DMPs, including two intersections: cg04658841 with annual NO_2_ and cg14464245 with MSAVI. Notably, cg14464245 was mapped to *AGPAT2*. Another intersection between annual NO_2_ and MSAVI involved cg17205313, which was mapped to the *TTC39C* (Table 2). Additionally, we examined consistency by intersecting results for each exposure across age groups. As shown in Figure S3, we did not observe any intersections across age groups for any of the exposures.

### Environmental exposures associated with DMRs

DMR analysis was conducted for all life stages and exposures. Significant DMRs were identified for nearly all exposures across all life stage groups, with exceptions for children aged 4–6 years and adolescents with O_3_, and children aged 8–10 years with MSAVI and LAN (Figure S4).

**Fig. 3:**
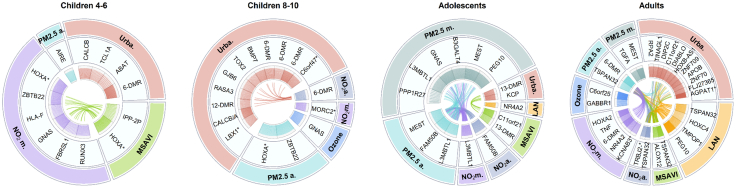
Overview of the significant differentially methylated regions (DMRs) across exposures, with a false discovery rate (FDR) threshold of 5%. Only DMRs with a minimum of 10 CpGs are displayed. Exposures are colour-coded in the outer circle, gene names are presented in the middle circle, and CpGs are shown in the inner circle. Connecting lines in the middle represent shared CpGs across different exposures. Results are shown by age group (*children 4–6 years: n = 717; children 8–10 years: n = 1061; adolescents: n = 878; adults: n = 5975*). Full details are provided in Supplemental Data 1. *Abbreviations:* PM2.5/NO_2_ a = annual; PM2.5/NO_2_ m = monthly; Urba. = Urbanicity; *HOXA∗ = HOXA-AS3, HOXA3, HOXA5; C6orf47∗ = C6orf47-AS1, C6orf47; LBX1∗ = LBX1-AS1, LBX1; MORC2∗ = MORC2-AS1; TRBJ2-∗ = TRBJ2-1/2/3; KCNAB3∗ = KCNAB3/TRAPPC1; AGPAT1∗ = RNF5, AGPAT1;* X-DMR = unannotated DMRs, with the initial number indicating the chromosome.

In children aged 4–6 years, monthly NO_2_ was associated with the 41.3% of significant DMRs (Fig. 3 and Supplemental Data 1). Among the significant DMRs with the highest number of CpGs, an association with urbanicity was identified on chromosome 6, and an association with MSAVI was observed on chromosome 7, each encompassing 30 CpGs. Annotation was available for a DMR on chromosome 7 (chr7:27183274-27184853) associated with MSAVI, mapped to the *HOXA-AS3, HOXA3, HOXA5*. Similarly, a DMR (chr7:27183401-27184521) associated with monthly NO_2_ exposure, also located on chromosome 7 and comprising a high number of CpGs (N = 23), was mapped also to *HOXA-AS3, HOXA3*. However, searching on the EWAS catalogue,^38^ both DMRs were found to be mapped only to *HOXA5*. Additionally, 23 CpGs were included in a DMR on chromosome 20 (chr20:57463572-57464973), which was specifically mapped to the *GNAS* complex locus. In children aged 8–10 years, urbanicity was associate to the 87.5% of DMRs; nevertheless, the results were comparable to those observed in children aged 4–6 years. When ranking the results by the number of CpGs included in the DMRs, the top five mapped to the same genes: *HOXA5, ZBTB22, CALCB/CALCA,* and *GNAS* (Table 3), but were generally associated with different exposures. Additionally, both age groups exhibited a DMR on chromosome 6, which is not mapped in the genome, and in both, it was associated with urbanicity. Overall, children aged 8–10 years show a higher number of DMRs associated with urbanicity compared to 4-years old children (Fig. 3). In adolescents monthly PM2.5 was associated with the 41.9% of significant DMRs (Fig. 3 and Supplemental Data 1). Consistently, these DMRs included the highest number of CpGs. On chromosome 7, one DMR (chr7:130131189-130132727) was mapped to the *MEST* gene and another DMR (chr7:94285942-94286669) to the *PEG10* gene, while on chromosome 20 a DMR (chr20:57463265–57464571) was mapped to the *GNAS* gene. Adult participants exhibited a substantial number of significant DMRs across nearly all exposures. The highest number of DMRs was identified in relation to urbanicity (57.7%); however, these did not correspond to the highest CpG counts. The DMR (chr7:94285642-94286936) with the greatest number of CpGs was associated with LAN exposure on chromosome 7, mapped to the *PEG10* gene, consistent with findings observed in adolescents (Fig. 3). The results were compared with those obtained using the ipDMR method, with findings reported in the Supplementary Material (section: “3.3.1 Comparison with ipDMR results”) and Supplemental Data 2.

**Table 3:** Comparison of the top DMR results in common between Children 4–6 years and children 8–10 years.

Children 4–6 years							Children 8–10 years						
CHR-pos^a^	N_CpGs	s-FDR^b^	meandiff^c^	Mapped gene^d^	EWAS catalogue^e^	Exposure	CHR-pos^a^	N_CpGs	s-FDR^b^	meandiff^c^	Mapped gene^d^	EWAS catalogue^e^	Exposure
chr6:30094947-30095802	30	9.06E-07	6.10E-04	NA		Urbanicity	chr6:30094980-30095802	21	3.59E-04	3.86E-04	NA		Urbanicity
chr7:27183274-27184853	30	1.14E-04	3.04E-01	HOXA-AS3, HOXA3, RP1-170O19.22, HOXA5	HOXA5	MSAVI	chr7:27183794-27184853	22	3.10E-03	−1.55E-02	HOXA-AS3, HOXA3	HOXA5	PM2.5 annual
chr7:27183401-27184521	23	4.00E-03	−3.07E-03	HOXA-AS3, HOXA3, RP1-170O19.22		NO_2_ monthly							
chr20:57463572–57464973	23	2.81E-08	−1.57E-03	GNAS, RP1-309F20.3	GNAS	NO_2_ monthly	chr20:57425979–57426425	17	1.76E-03	−1.61E-03	GNAS	GNAS; GNASAS	Ozone
chr6:33282856-33283317	20	5.39E-03	−2.14E-03	ZBTB22	ZBTB22; TAPBP	NO_2_ monthly	chr6:33282624-33283987	28	1.16E-05	−1.10E-02	ZBTB22	ZBTB22; TAPBP	PM2.5 annual
chr11:14993818–14994397	15	2.14E-03	4.07E-04	CALCB, CALCA	CALCA	Urbanicity	chr11:14993378–14994644	21	8.08E-10	3.62E-04	CALCB, CALCA	CALCA	Urbanicity

a CHR-pos = chromosome:start-end.

b s-FDR = Smooths the FDR values across the identified regions.

c Meandiff = mean differences.

d Mapped gene = annotation provided by Illumina manifest file.^31^

e EWAS catalogue = annotation provided by the EWAS catalogue.^38^

### Common DMRs across exposures

**Fig. 4:**
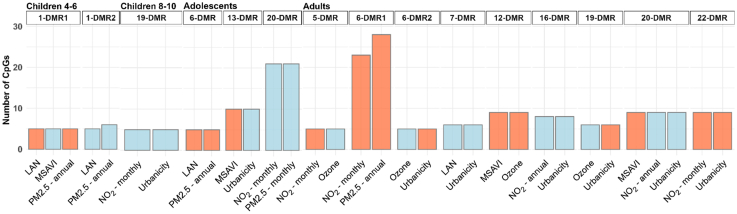
Bar plots of common DMRs among different exposures, grouped by age group *(children 4–6 years: n = 717; children 8–10 years: n = 1061; adolescents: n = 878; adults: n = 5975).* On the y-axis is reported the number of CpGs for each DMRs-exposure associated. On the x-axis, are reported the different exposures. DMRs with positive mean differential values (average increase in methylation) are shown in red; those with negative values (average decrease) are shown in blue. Results are organised by chromosome number and all included DMRs meet the criterion of a minimum smoothed FDR of <0.05. The common DMRs are labelled as “X-DMRY”, where “X” indicates the chromosome number and ‘DMRY’ denotes the progressive count of methylated regions on that chromosome.

DMRs common across different exposures were identified for all life stages, as shown in Fig. 4 (with further details in Supplemental Data 1), which also illustrates the methylation status of these DMRs, represented by the mean differential.

**Fig. 5:**
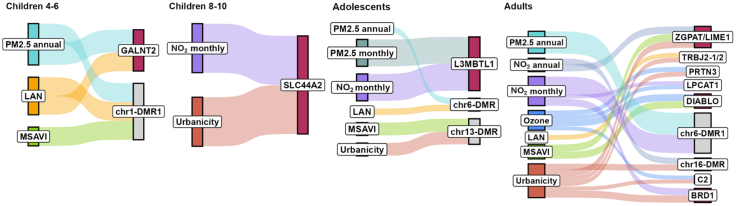
Sankey plot illustrating the common DMRs across exposures and the corresponding mapped gene. Exposures are colour-coded, and the connection width represents the number of CpGs included in the common DMRs (thicker connection indicates higher counts). The non-annotated DMRs are labelled as “chrX-DMRY”, where “chrX” indicates the chromosome number and ‘DMRY’ denotes the progressive count of methylated regions on that chromosome. Results are shown by age group *(children 4–6 years: n = 717; children 8–10 years: n = 1061; adolescents: n = 878; adults: n = 5975).*

In children aged 4–6 years, two shared DMR-exposure associations were observed, both located on chromosome 1 (Figs. 4 and 5). The DMR (chr1:230414987-230415547) in common between LAN and annual PM2.5, which showed a decreased average methylation in both, was mapped on *GALNT2*. The DMR (chr1:111218079-111218554) common among LAN, MSAVI, and PM2.5 showed no mapped gene. It showed an average increase in methylation with LAN and annual PM2.5, and a decrease one with MSAVI. Children aged 8–10 years reported one shared DMR-exposure association, located on chromosome 19. The chr19-DMR showed an average decrease in methylation with both monthly NO_2_ and urbanicity and it is mapped to the *SLC44A2* gene. Among adolescents, three shared DMR-exposure associations were identified on chromosomes 6, 13, and 20. The chr20-DMR (chr20:42142596-42143502), mapped to the *L3MBTL1* gene, contained the highest number of CpGs (N = 21). No annotated genes were identified for the other two DMRs, one in chromosome 6 (chr6:33175140-33175225) and 13 (chr13:47471705–47472429).

Adults exhibited the highest number (N = 9) of shared DMR-exposure associations. Chromosome 6 included the DMR (chr6:30094947-30095802) with highest number of CpGs, in common between PM2.5 annual (N = 28) and NO_2_ monthly (N = 23), in both an average increase in methylation was observed. No annotated gene was identified for this region. To follow, chr12-DMR, is shared between MSAVI and O_3_ exposure (both with average increase in methylation), and is mapped to the *DIABLO* gene. On chromosome 20, a shared DMR was observed for MSAVI (average increase in methylation), annual NO_2_ and urbanicity (both average decrease in methylation), is mapped to the *ZGAPT* and *LIME1* genes. An average increase in methylation was observed in the chr22-DMR, which was associated with monthly NO_2_ and urbanicity. This region mapped to the *BRD1* gene. Other common DMR-exposure associations are shown in Figs. 4 and 5 (detailed in Supplemental Data 1).

In general, the direction of methylation, either positive or negative, remains consistent for exposures associated with the urban environment compared to MSAVI.

### Pathway enrichment analysis

We investigated the biological responses to various exposures across different life stages. Pathway analysis was conducted based on eQTM-mapped genes from CpGs located within DMRs. Results, derived from GO Biological Process and KEGG are presented in Supplemental Data 3 and Figures S5–S6 across all life stages and exposures. The results from the two pathway resources were comparable. GO showed more enriched biological processes, while KEGG identifies fewer, with largely overlapping content. The only exception was in adolescents, where KEGG showed no results, but GO highlights enrichment in “Protein Modification By Small Protein Conjugation” and “Protein Ubiquitination” in response to annual PM2.5 (Figures S5 and S6). In children 4–6 years, significantly enriched pathways were mainly related to immune and infectious disease subcategory in response to monthly NO_2_ and urbanicity. No significantly enriched pathways were observed for children aged 8–10 years. In adults, the mapped genes were predominantly enriched in immune and infectious disease and cancer related pathways in response to monthly NO_2_, O_3_ and urbanicity (Supplemental Data 3, Figures S5 and S6). Using the KEGG database, the pathways related to immune response were comparable between children and adults. This overlap was mainly driven by the presence of *HLA-E* and complement component genes (*C4A* and *C4B*) in both age groups, which contributed to the same pathways.

As a sensitivity analysis, we compare these results to those obtained using MissMethyl R package, which performs the enrichment analysis directly at CpGs level. The findings from both methods were comparable. Specifically, in children 4–6 years, significant results were observed in response to monthly NO_2_, and the enriched pathways identified were related to immune and infectious disease as observed using the gene mapped in the BIOS dataset (Supplemental Data 4). In adults, “PD-L1 expression and PD-1 checkpoint pathway in cancer” resulted to be significant in response to urbanicity, and this finding was also confirmed using BIOS mapped genes (Supplemental Data 4).

## Discussion

Understanding the impact of the urban environment on human biology is crucial for maintaining human health and for improving causal understanding towards prioritising targets in urban public health and better city planning. The dynamic nature of DNA methylation plays a key role in elucidating how the urban exposome influences health throughout life. In our work, we investigated the association of the urban exposome with differences in DNA methylation level in blood from childhood to adulthood.

In children aged 4–6 years, our results indicated a predominant response to monthly NO_2_ exposure across all analyses. When examining the same children at ages 8–10, we observed that methylation differences were primarily associated with urbanicity. Interestingly, comparing the results between these two life stages revealed that ranking DMRs by the number of included CpGs, they annotated to the same loci, but in association to different exposures. For example, in children aged 4–6 years, the DMRs annotated to the *HOXA5* gene included the highest number of CpGs in association to MSAVI, and another DMR, with a smaller number of CpGs, in response to monthly NO_2_ exposure. In children aged 8–10 years, the same *HOXA5*-mapped DMR was instead associated with annual PM2.5 exposure. Notably, the association with MSAVI indicated an average increase in methylation, whereas both monthly NO_2_ and annual PM2.5 indicated a decrease one. *HOXA5* is part of transcription factors called homoeobox genes, and it may regulate gene expression, morphogenesis, and differentiation.^39^ A previous study has shown the involvement of *HOXA5* in predicting COPD and asthma in adulthood based on cord blood DNA methylation measured in newborns.^40^ Other studies found changes in *HOXA5* methylation in relation to circulating triglycerides level and the adipose tissue, thus leading to metabolic condition.^41^^,^^42^ In addition, DMR located to *HOXA5* was found associated with long-term PM2.5.^43^ However, almost all the studies have been conducted in adults and cord blood, while findings in children are still limited. This shared methylation pattern in children may be attributed to the fact that the same children were analysed at different ages, suggesting either that some methylation signals remain stable over the four-year period, or it could reflect changes in habits of the children with ageing.

In adolescents, a more consistent signal was observed in response to monthly PM2.5 exposure across the analyses. As in children, a DMR localised near the *GNAS* gene was also identified in this group. However, in general, the results appear more analogous to those observed in adults. This observation aligns with our data, considering that the NTR cohort, which contributed the largest number of participants, includes individuals starting from 18 years old. Particularly, *PEG10* and *MEST* genes were mapped to DMRs in both adolescents and adults. In adolescents, monthly and annual PM2.5 was associated with *MEST* and *PEG10*. Additionally, *L3MBTL1* was mapped to multiple DMRs in response to both monthly and annual PM2.5 exposure, as well as monthly NO_2_ exposure. *GNAS, PEG10, MEST* and *L3MBTL1* are all imprinted genes, only the copy from either the mother or the father is expressed.^44^ A previous study conducted in young adults in Guangzhou city, China^45^ has found that constituents of PM2.5, such as transition metals from traffic sources, were associated with DNA methylation differences in imprinted genes, including *GNAS*, *PEG10*, *MEST* and *L3MBTL1*. In summary, they reported that short-term PM2.5 exposure resulted in a decrease in methylation of their candidate genes (including *L3MBTL1*, *PEG10*, *GNAS, MEST*) which we have observed in our results as well (Supplemental Data 1). Specifically, the work highlighted the sensitivity of *L3MBTL1* to PM2.5 constituents. This gene encodes for a protein that regulates gene activity via chromatin modification, which may also be necessary for mitosis.^39^

The imprinted genes *PEG10* and *MEST* were also observed in adults in response to LAN and monthly PM2.5, respectively. In adults the DMR near to *PEG10* gene contains the highest number of CpGs. Overexpression of *PEG10* has been associated with multiple malignancies.^39^ Additionally, a persistent signal was detected for DMRs located near *TSPAN32* and *C11orf21* genes in association with annual PM2.5 and NO_2_, LAN (associated with average decrease in methylation) and MSAVI (associated with average increase in methylation). *TSPAN32* and *C11orf21*, both located in the 11p15.5 imprinted region on chromosome 11, play key roles in haematopoietic regulation and cancer.^39^ Furthermore, a recent publication^46^ demonstrated the multifaced role of *TSPAN32* in immune regulation and cellular metabolism. In summary, adults exhibited a greater number of DNA methylation differences associated with all exposures, compared to other life stages across all analyses. We speculate that this may reflect age-related biological processes, including an increased susceptibility to disease related to harmful environmental exposures. Older adults with pre-existing comorbidities may be more vulnerable to the effects of urban environmental exposures and the cumulative impact of the exposome. Additionally, it is important to consider the large number of adult participants included in this study, which increase the statistical power.

In all life stages, we observed that the DMRs associated with exposures reflecting air pollution, LAN, and urbanicity had the same methylation direction. In contrast, MSAVI, reflecting greenness, always showed the opposite direction. This pattern was further observed in adults’ sensitivity analyses, where MSAVI and annual NO_2_ were associated with the same DMP (cg17205313) but with opposite directions. The excluded cohort had lower air pollution levels, which may partly explain this difference. The only exception was O_3_ (warm season), which went in the opposite direction of urban exposures. O_3_ is a secondary pollutant generated from NOx, and its concentrations can be higher in rural areas than in urban regions, especially during the warmer season.^47^ In addition, prior work^48^^,^^49^ indicates that short- and long-term exposures have distinct associations with blood DNA methylation depending on developmental stage, which may explain the limited overlap we observed between monthly and annual exposure windows.

Interpreting the biological significance of DNA methylation can be challenging. Our analysis of CpGs from the DMRs revealed mainly significantly enriched pathways exclusively in children aged 4–6 years and in adults. Children are more vulnerable to air pollution than adults due to their smaller lungs, faster breathing rates, and developing organs and immune systems.^50^ In children aged 4–6 years, the resulting pathways suggested an impact on the immune system and perhaps autoimmune diseases in response to monthly NO_2_ exposure. The underlying biological mechanisms include oxidative stress and inflammation induced by airway damage as a consequence of air pollution.^51^ This was also observed in adults, with the addition of one enriched pathway in PD-L1 expression and the PD-1 checkpoint in cancer. This pathway is a key regulator in immune system balance, preventing overactivation and it can be exploited by cancer cells to escape immune detection.^52^

To summarise, in this study, we explored the association of the urban exposome with DNA methylation across the life course. We analysed methylation data from multiple cohorts across Europe at different ages, with harmonised exposure data modelled within EXPANSE identically for each cohort. Our results suggested that urban environmental factors leave DNA methylation marks in younger children and adults. Children exhibited DNA methylation differences principally in association with monthly NO_2_ exposure, whereas adults showed methylation signals across nearly all exposures, with a pronounced effect with urbanicity.

However, although exposures were analysed individually, disentangling specific exposure-related DNA methylation signatures in an urban environment remains challenging, as some DMRs were associated with multiple exposures. One of our key findings was the opposing direction of methylation differences in common DMRs when comparing urban exposome-related exposures with MSAVI, an indicator of greenness. This suggests that urban environmental factors and greenness may have contrasting effects on DNA methylation. This study provides novel insights into DNA methylation changes in response to the urban exposome, and suggests epigenetics as a promising tool to map environmental influence in both children and adults.^53^ To the best of our knowledge, most previous studies have focused on single exposures at a specific life stage, and evidence spanning different life stages remains scarce. Our findings contribute to a better understanding of how environmental factors can shape DNA methylation. By integrating multiple domains of the urban exposome across age groups within a single-exposure approach, this study helps address this research gap and highlights the challenges of identifying methylation signatures associated with specific exposures. Nevertheless, several limitations should be considered. The cross-sectional design cannot establish causal associations between exposures and DNA methylation pattern. Additionally, urbanicity, defined based on population density, may reflect a mixture of physical-chemical exposures and level of greenness, making difficult to interpret it as a single exposure. Future studies on cumulative exposure assessments and longitudinal designs will be essential to fully capture the dynamic interplay between environment and the methylome. Moreover, our study did not account for other urban exposome factors (e.g., social context), which may also influence DNA methylation. Finally, the adulthood life stage covers a broad age range, therefore we cannot draw an inference regarding a vulnerable time window.

## Contributors

Megi Vogli: Conceptualisation, Data curation, Formal analysis, Methodology, Project administration, Visualisation, Writing–review & editing, Writing–original draft. Ayoung Jeong: Conceptualisation, Data curation, Formal analysis, Methodology, Writing–review & editing. Zhebin Yu: Data curation, Formal analysis, Writing–review & editing. Judith M. Vonk: Data curation, Methodology, Formal analysis, Writing–review & editing. Dorina Ibi: Data curation, Formal analysis, Writing–review & editing. Jaanika Kronberg: Data curation, Formal analysis, Writing–review & editing. Petr Gregor: Data curation, Formal analysis, Writing–review & editing. Lisa Maier: Data curation, Formal analysis, Writing–review & editing, Miriam Leskien: Formal analysis, Writing–review & editing. Marta Cirach: Data curation, Writing–review & editing. Payam Dadvand: Data curation, Writing–review & editing. Ondřej Mikeš: Data curation, Writing–review & editing. Olena Gruzieva: Data curation, Writing–review & editing. Ulrike Gehring: Data curation, Project administration, Funding acquisition, Writing–review & editing. Kathrin Wolf: Data curation, Methodology, Writing–review & editing. Melanie Waldenberger: Data curation, Methodology, Writing–review & editing. Medea Imboden: Conceptualisation, Project administration, Writing–review & editing. Pavel Čupr: Data curation, Funding acquisition, Writing–review & editing. Kees de Hoogh: Data curation, Writing–review & editing. Gerard H. Koppelman: Data curation, Methodology, Writing–review & editing. Erik Melén: Data curation, Project administration, Funding acquisition, Writing—review & editing. Regina Pickford: Investigation, Project administration, Supervision, Writing–review & editing. Elisabeth Thiering: Data curation, Methodology, Writing–review & editing. Marie Standl: Data curation, Investigation, Methodology, Project administration, Resources, Writing–review & editing. Estonian Biobank Research Team: Data acquisition, Data curation. Writing–review & editing. Jana Klánová: Data acquisition, Resources, Funding acquisition, Writing–review & editing. Jelle Vlaanderen: Conceptualisation, Data acquisition; Methodology; Funding acquisition, Project administration, Writing–review & editing. Roel Vermeulen: Conceptualisation, Data acquisition; Methodology; Funding acquisition, Project administration, Writing–review & editing. Nicole Probst-Hensch: Conceptualisation, Data acquisition; Methodology; Project administration; Writing–review & editing. Annette Peters: Conceptualisation, Data acquisition; Investigation, Methodology; Project administration; Resources, Funding acquisition, Writing–review & editing. The EXPANSE Consortium contributed to the overall study design, coordination, data harmonisation, and exposure assessment across participating cohorts. All biological and health data were already available within the individual cohorts prior to the project.

All authors who conducted the formal analyses had access to the all the underlying data and verified their accuracy. All authors read and approved the final version of the manuscript.

## Data sharing statement

Methylation data from each cohort study are not publicly available due to local ethics committee requirements and data protection regulations. Access to individual-level data requires approval from the respective cohort's data access committee. Summary statistics from each contributing study and the METAL meta-analysis files generated for this work are available from the corresponding author upon reasonable request.

## Declaration of interests

Gerard H. Koppelman reports grants paid to his institution from the Netherlands Lung Foundation, OMBION-CPBT Growth Funds the Netherlands, the EU Horizon 2020 programme (Respire XL), TEVA the Netherlands, ZON-MW (VICI-grant), and Vertex; consulting fees paid to his institution from AZ and PURE IMS; payments for lectures and presentations paid to his institution from Boehringer-Ingelheim, Sanofi, and AZ; and serving as the unpaid chair and founder of the exquAIro foundation. Other authors declare that they have no known competing financial interests or personal relationships that could have appeared to influence the work reported in this paper.

PIAMA. Supported by The Netherlands Organisation for Health Research and Development; The Netherlands Organisation for Scientific Research; The Lung Foundation of the Netherlands (grant number AF 45.1.14.001 supported the blood DNA methylation assays at age 16 years); The Netherlands Ministry of Spatial Planning, Housing, and the Environment; and The Netherlands Ministry of Health, Welfare, and Sport. Blood DNA-methylation analysis at age 4 and 8 was supported by the MeDALL study (EU FP7-CP-IP; Project No: 261357).

BAMSE. Funded by the Swedish Research Council (grant no. 2020-01886, 2022-06340, 2024-02345), the Swedish Research Council for Health, Working Life and Welfare (FORTE grant no.2017-01146, no.2023-01213), the Swedish Heart-Lung Foundation, Karolinska Institute (no. 2022-01807) and Region Stockholm (ALF project for cohort and database maintenance).

LISA. Initially supported by the Federal Ministry for Education, Science, Research and Technology and additionally by Helmholtz Zentrum Munich (former GSF), Helmholtz Centre for Environmental Research–UFZ, Leipzig, Research Institute at Marien-Hospital Wesel, Pediatric Practice, Bad Honnef. Follow-ups at 4, 6, 10 and 15 years were funded by the respective budgets of participating partners (Helmholtz Zentrum Munich, Helmholtz Centre for Environmental Research–UFZ, Leipzig, Research Institute at Marien-Hospital Wesel, Pediatric Practice, Bad Honnef, IUF—Leibniz-Research Institute for Environmental Medicine at the University of Düsseldorf) and by grant from the Federal Ministry for Environment (IUF Düsseldorf, FKZ 20462296). Additional support came from the ALLERGEN ERC grant (grant agreement number 949906).

CELSPAC: YA. Supported by RECETOX Research Infrastructure (no. LM2023069) financed by the Ministry of Education, Youth and Sports, and the Operational Programme Research, Development and Education (the CETOCOEN EXCELLENCE project No. CZ.02.1.01/0.0/0.0/17_043/0009632 and Cetocoen Plus CZ.02.1.01/0.0/0.0/15_003/0000469). This work has been supported from project the Horizon Europe program No 101096888 (DISCERN). Laboratory work was supported by the project BBMRI.cz (LM2023033). Additional funding came from the EU Horizon 2020 program under grant agreement No 857560 (CETOCOEN Excellence) and OP JAK project AGEING-CZ (no. CZ.02.01.01/00/23_025/0008743), co-funded by EU.

NTR. Supported by the Netherlands Organisation for Scientific Research (NWO): Biobanking and Biomolecular Research Infrastructure (BBMRI-NL, NWO 184.033.111) and the BBRMI-NL-financed BIOS Consortium (NWO 184.021.007), NOW grants 480–15-001/674; 480–04-004; 400–05-717; the European Science Council (ERC Advanced, 230374, Genetics of Mental Illness); and NIMH grant 1RC2 MH089995 (Developmental trajectories of psychopathology). JvD was supported by NWO Large Scale infrastructures, X-omics (184.034.019).

Estonian Biobank. Research was conducted using the Estonian Centre of Genomics/Roadmap II funded by the Estonian Research Council (project number TT17).

SAPALDIA. Supported by the Swiss National Science Foundation (grants no 33CS30-177506/1, 33CS30-148470/1&2, 33CSCO-134276/1, 33CSCO-108796, 324730_135673, 3247BO-104283, 3247BO-104288, 3247BO-104284, 3247-065896, 3100-059302, 3200-052720, 3200-042532, 4026-028099, PMPDP3_129021/1, PMPDP3_141671/1), the Federal Office for the Environment, the Federal Office of Public Health, the Federal Office of Roads and Transport, the canton's government of Aargau, Basel-Stadt, Basel-Land, Geneva, Luzern, Ticino, Valais, and Zürich, the Swiss Lung League, the canton's Lung League of Basel Stadt/Basel Landschaft, Geneva, Ticino, Valais, Graubünden and Zurich, Stiftung ehemals Bündner Heilstätten, SUVA, Freiwillige Akademische Gesellschaft, UBS Wealth Foundation, Talecris Biotherapeutics GmbH, Abbott Diagnostics, Klinik Barmelweid, Hirslanden Klinik Aarau, European Commission 018996 (GABRIEL), Wellcome Trust WT 084703 MA, Exposomics EC FP7 grant (Grant agreement No: 308610).

KORA. Initiated and financed by the Helmholtz Zentrum München–German Research Centre for Environmental Health, which is funded by the German Federal Ministry of Education and Research (BMBF) and by the State of Bavaria.

## Appendix A Supplementary data

Supplementary Material

EXPANSE consortium
