# Supplementary material for: The impact of environmental exposures on DNA methylation in the EXPANSE project
Source: eBioMedicine. 2025 Dec 19;123:106084. doi: 10.1016/j.ebiom.2025.106084 (PMC12794040; doi:10.1016/j.ebiom.2025.106084)
Supplement: Supplementary Material [file mmc5.docx]

Supplementary material for:

“**The impact of environmental exposures on DNA methylation across the life course in the EXPANSE project”**

Table of contents:

1. Genes Official Full Name
2. Supplementary Methods

2.1 Description of the participating mature birth cohorts

2.2 Description of the participating adult cohorts

3. Supplementary results

3.1 Descriptive statistics of exposure variables

3.2 Meta-analysis of epigenome-wide association studies

3.2.1 CELSPAC: Young Adults (YA) cohort

3.2.2 Summary tables of meta-analysis results meeting the suggestive significance threshold

3.2.3 Integrative Expression Quantitative Trait Methylation (eQTM) Analysis

3.2.4 Intersection analysis by Age Group for the different exposures

3.2.5 Sensitivity analysis in the adults age group: exclusion of the Estonian Biobank

3.3 DMR Results:

3.3.1 Comparison with ipDMR results

3.4 Pathway enrichment analysis

4. References

1. **Genes Official Full Name (1)**

| **Gene symbol** | **Genes Official Full Name** |
| --- | --- |
| TTC39C | tetratricopeptide repeat domain 39C |
| C2CD4C | C2 calcium dependent domain containing 4C |
| STX16 | syntaxin 16 |
| UBR7 | ubiquitin protein ligase E3 component n-recognin 7 |
| SLC10A7 | solute carrier family 10 member 7 |
| BAZ2A | bromodomain adjacent to zinc finger domain 2A |
| PPP2R2B | protein phosphatase 2 regulatory subunit beta |
| AGPAT2 | 1-acylglycerol-3-phosphate O-acyltransferase 2 |
| HOXA-AS3 | HOXA cluster antisense RNA 3 |
| HOXA3 | homeobox A3 |
| HOXA5 | homeobox A5 |
| ZBTB22 | zinc finger and BTB domain containing 22 |
| CALCA | calcitonin related polypeptide alpha |
| CALCB | calcitonin related polypeptide beta |
| MEST | mesoderm specific transcript |
| PEG10 | paternally expressed 10 |
| SLC16A3 | solute carrier family 16 member 3 |
| GALNT2 | polypeptide N-acetylgalactosaminyltransferase 2 |
| RUNX3 | RUNX family transcription factor 3 |
| HLA-E | major histocompatibility complex, class I, E |
| STOX2 | storkhead box 2 |
| SSH1 | slingshot protein phosphatase 1 |
| SLC44A2 | solute carrier family 44 member 2 (CTL2 blood group) |
| L3MBTL1 | L3MBTL histone methyl-lysine binding protein 1 |
| DIABLO | diablo IAP-binding mitochondrial protein |
| ZGPAT | zinc finger CCCH-type and G-patch domain containing |
| LIME1 | Lck interacting transmembrane adaptor 1 |
| BRD1 | bromodomain containing 1 |
| TSPAN32 | tetraspanin 32 |
| C11orf21 | chromosome 11 open reading frame 21 |

1. **Supplementary Methods**
   1. **Description of the participating mature birth cohorts**

**PIAMA**

**Cohort description**

PIAMA is a population-based prospective birth cohort study with detailed descriptions published previously (3, 4). In brief, pregnant women were recruited from communities in different regions of the Netherlands in 1996–1997, and their children (N = 3963) were followed up by repeated questionnaire surveys (parental-completed at age 3 months and then annually until age 8, from age 11 onwards every 3 years parent- and -participant completed, and at age 20 participant completed only) including questions about health, demographic factors and risk factors for asthma and respiratory health. The institutional review boards of the participating institutes approved the study protocol and written informed consent was obtained from the parents or legal guardians of all participants. Medical exams including collection of blood were performed in sub-populations at ages 4, 8, 12, and 16 years.

**Methylation data**

Peripheral blood samples were collected from all consenting cohort participants and DNA was extracted using the QIAamp blood kit. At age 4 and 8, DNA methylation was measured in selected samples based on a paired case-control design, with cases being defined by the presence of one allergic disease. At age 16, DNA methylation was measured in all available samples. DNA methylation data at age 4 and 8 years were obtained using the Illumina 450 K array as part of the MeDALL study (5). DNA methylation data form whole blood at age 16 years was obtained in an independent experiment, using the Illumina 450 K array, with the same QC protocol as described below (5). The study was approved by the Ethical Committees of the participating centers and written informed consent was obtained of the participating children and their legal guardians.

The methylation data were pre-processed in R with the Bioconductor package Minfi1, using the original IDAT files extracted from the HiScanSQ scanner. Samples that did not provide significant methylation signals in more than 10% of probes (detection P=0.01) were excluded from further analysis. Samples were also excluded in cases of low staining efficiency, low single base extension efficiency, low stripping efficiency of DNA from probes after single base extension, poor hybridization performance, poor bisulphite conversion and high negative control probe staining. Further, we used the 65 SNP probes to check for concordances between paired DNA samples from the same individual and assessed the methylation distribution of the X-chromosome to verify gender. Paired samples with Pearson correlation coefficients <0.9 were regarded as sample mix-ups and were excluded from the study. In probe filtering, we excluded probes on sex chromosomes, probes that mapped on multi-loci, the 65 random SNPs assay and probes that contained SNPs at the target CpGs with a minor allele frequency >10%. The allele frequencies of a list of SNPs were obtained from 1000 Genomes, release 20110521 for the CEU population. Finally, to correct Type I and Type II bias, we implemented “DASEN”3 to perform signal correction and normalization. After quality control, 439,306 autosomal probes remained.

**Funding and acknowledgement**

The PIAMA study is supported by The Netherlands Organization for Health Research and Development; The Netherlands Organization for Scientific Research; The Lung Foundation of the Netherlands (grant number AF 45.1.14.001 supported the blood DNA methylation assays at age 16 years); The Netherlands Ministry of Spatial Planning, Housing, and the Environment; and The Netherlands Ministry of Health, Welfare, and Sport. The blood DNA-methylation analysis at age 4 and 8 was supported by the MeDALL study (Mechanisms of the Development of ALLergy; EU FP7‐CP‐IP; Project No: 261357).

We thank all the children and their parents for their cooperation. We also thank all the field workers and laboratory personnel involved for their efforts, and Marjan Tewis and Marjan Kerkhof for data management.

**Ethical approval**

Medical Ethics Committee (METC) approval for PIAMA for the start of the project and the relevant follow-ups:

Start Project:

- Rotterdam, MEC (Medisch Ethische Commisie Erasmus Universiteit Rotterdam/Academische Ziekenhuizen Rotterdam) 132.636/1994/39, 13 juni 1994 and 137.326/1994/130, Febr 16th 1995.

- Groningen, MEC (Medisch Ethische Commisie Academisch ziekenhuis Groningen) 94 / 08 / 92, August 26th, 1994.

- Utrecht/Bilthoven, MEC-TNO (Medisch Ethische Commisie -Toegepast Natuurwetenschappelijk Onderzoek) 95/50, Febr 28th 1996.

Age 4 years

Utrecht, CCMO (Centrale Commissie Mensgebonden Onderzoek) P000777C, Sept 25th, 2000.

Age 8 years

Utrecht, CCMO (Centrale Commissie Mensgebonden Onderzoek) P04.0071C, August 5th, 2004 (Utrecht, METC-protocol number 04 - 101 / K, July 27th, 2004; Rotterdam, P04.0071C/MEC 2004-152, July 1st, 2004; Groningen, P04.0071C/ M 4.019912, June 28th, 2004).

Age 16 years

Utrecht, METC (Medisch Ethische ToetsingsCommissie) protocol number 12-019/K, May 25th, 2012.

Amendement 1, July 12th, 2012; Amendement 2, September 20th, 2012.

Groningen, METC (Medisch Ethische ToetsingsCommissie) protocol number 12-019/K, Amendement, August 16th, 2012.

**BAMSE**

**Cohort description**

BAMSE (Children, Allergy, Milieu, Stockholm, Epidemiology in Swedish) is a prospective population-based cohort study of children recruited at birth and followed during childhood. Details of the study design, inclusion criteria, enrolment and data collection are described elsewhere (6-8). In short, 4,089 children born between 1994 and 1996 in four municipalities of Stockholm County were enrolled. At baseline, when the infant was approximately 2 months of age, parents completed a questionnaire that assessed residential characteristics, as well as socioeconomic and lifestyle factors. When children were 1, 2, 4, 8, 16 the parents completed questionnaires focusing on children’s symptoms related to wheezing and allergic diseases, as well as various exposures. The survey response rates were 96%, 94%, 91% and 84%, respectively. Furthermore, blood was obtained at ages 4, 8 and 16. The baseline and follow-up studies were approved by the Regional Ethical Review Board, Karolinska Institutet, Stockholm, Sweden, ethical approval number is 2010/1474-31/3. The parents of all participating children provided written informed consent.

**Methylation data**

For this methylation study, we used data from the 4-, 8- and 16-year follow-up. At 4 years, epigenome-wide DNA methylation was measured in 256 Caucasian children, at 8 years methylation was measured in 472 Caucasian children and at 16 years, methylation was measured in 269 Caucasian children. 500 ng DNA per sample underwent bisulfite conversion using the EZ-96 DNA Methylation kit (Zymo Research Corporation, Irvine, USA). Samples were plated onto 96-well plates in randomized order. Samples were processed with the Illumina Infinium HumanMethylation450 BeadChip (Illumina Inc., San Diego, USA). Quality control of analysed samples was performed using standardized criteria. At 4 years blood methylation data were produced in the Genome Analysis Facility of the University Medical Center Groningen (UMCG) in Holland as part of the MeDALL (Mechanisms of the Development of Allergy) project. DNA methylation data were pre-processed by using the minfi R from the original idat files (9). Samples that did not provide significant methylation signals in more than 10% of probes (detection P>0.01) were excluded from further analysis. Samples were also excluded in cases of low staining efficiency, low single base extension efficiency, low stripping efficiency of DNA from probes after single base extension, poor hybridization performance, poor bisulphite conversion and high negative control probe staining. For 8 and 16 years, samples were excluded in case of sample call rate <99%, colour balance >3, low staining efficiency, poor extension efficiency, poor hybridization performance, low stripping efficiency after extension and poor bisulfite conversion. We also applied multidimensional scaling (MDS) plot to evaluate gender outliers based on chromosome X data, that produced two separated clusters for male and female. Samples that did not belong to the distinct cluster were removed. Furthermore, we applied median intensity plot for methylated and unmethylated intensity by using the minfi R package (samples below the 10.5 cutoff were excluded). Applying these criteria resulted in exclusion of 8 (8-year DNA) and 2 (16-year DNA) samples, respectively. Probes with a single nucleotide polymorphism in the single base extension site with a frequency of > 10 % at 4 years and of >5% at 8 and 16 years were excluded, as were probes with non-optimal binding (non-mapping or mapping multiple times to either the normal or the bisulphite-converted genome), and the probed belonging to chr X and chr Y, resulting in the exclusion of 46,206, 46,799 and 47,654 probes (4-,8- and 16-year DNA), leaving a total of 439,306, 438,713 and 437,858 probes, respectively, in the analyses. Furthermore, we implemented “DASEN” recommended from wateRmelon package to do signal correction and normalization. For cell type correction, We used the Reinius-based Houseman method (10, 11) with the estimateCellCounts function in the Minfi package in R to estimate relative proportions of six white blood cell subtypes (CD4+ T-lymphocytes, CD8+ T-lymphocytes, NK (natural killer) cells, B-lymphocytes, monocytes and granulocytes). Batch correction was attained including the significant (permutation p-value< 10-4) principal components derived from the 613 negative control probes presented in 450K arrays. After 10.000 permutations 5 PCs were retained and in additional one batch was also accounted for in the models, based on the bisulfite treatment. The beta-values were batch corrected incorporating these 5 PCs and calculating the residuals of the linear model at 4 years. The covariate batch was also accounted for in the models, based on the bisulfite treatment date at 8 years. At 16 years, the empirical Bayes method via ComBat was applied for batch correction based on sample plate and sentrix position using the sva package in R.

**Funding and acknowledgement**

The BAMSE study received funding from the Swedish Research Council (grant no. 2020-01886, 2022-06340, 2024-02345), the Swedish Research Council for Health, Working Life and Welfare (FORTE grant no.2017-01146, no.2023-01213), the Swedish Heart-Lung Foundation, Karolinska Institute (no. 2022-01807) and Region Stockholm (ALF project for cohort and database maintenance). We thank the children and parents participating in the BAMSE cohort and all staff involved in the study through the years.

**LISA**

**Cohort description**

The influence of Life-style factors on the development of the Immune System and Allergies in East and West Germany (LISA) study is a population-based birth cohort study. A total of 3,094 healthy, full-term neonates were recruited between 1997 and 1999 in Munich, Leipzig, Wesel and Bad Honnef. The participants were not pre-selected based on family history of allergic diseases and detailed descriptions of the LISA study have been published elsewhere (12). Approval was given by the local Ethics Committees and written consent from participant’s families was obtained. A total of 234 and 226 participants from the 6- and 10-year follow-up with available DNA methylation and exposure data were included in this study. The ethics committee is Bavarian Board of Physicians, numbers are 07098 for 10-year follow-up and 03166 for 6-year follow-up.

**Methlyation data**

Blood samples were collected at the age of 6 and 10 years. DNAm was measured in blood using the MethylationEPIC BeadChip (Illumina, Inc., San Diego, CA). Details are described elsewhere (13).

**Funding & Acknowledgement**

The LISA study was mainly supported by grants from the Federal Ministry for Education, Science, Research and Technology and in addition from Helmholtz Zentrum Munich (former GSF), Helmholtz Centre for Environmental Research - UFZ, Leipzig, Research Institute at Marien-Hospital Wesel, Pediatric Practice, Bad Honnef for the first 2 years. The 4 year, 6 year, 10 year and 15 year follow-up examinations of the LISA study were covered from the respective budgets of the involved partners (Helmholtz Zentrum Munich (former GSF), Helmholtz Centre for Environmental Research - UFZ, Leipzig, Research Institute at Marien-Hospital Wesel, Pediatric Practice, Bad Honnef, IUF – Leibniz-Research Institute for Environmental Medicine at the University of Düsseldorf) and in addition by a grant from the Federal Ministry for Environment (IUF Düsseldorf, FKZ 20462296). Further, this research has received funding from ALLERGEN ERC grant (grant agreement number 949906).

The authors thank all families for participation in the studies and the LISA study teams for their excellent work.

- 1. **Description of the participating adult cohorts**

**CELSPAC: YA**

**Cohort description**

The Central European Longitudinal Studies of Parents and Children: Young Adults (CELSPAC: YA) study which is an ongoing follow-up re-examination of the Czech part of the ELSPAC birth cohort (European Longitudinal Study of Pregnancy and Childhood) that was initiated in 1991–1992 in the Czech Republic. Detailed information about the ELSPAC-CZ study is provided in (14). The CELSPAC: YA study longitudinally collected a broad spectrum of data including lifestyle and health questionnaires, biological samples, and their chemical analysis. Our work examined CELSPAC: YA participants that had available all input data for the analysis (EWAS analysis on saliva samples), i.e. 278 participants from a total of 3 sub cohorts: STOMA (average age 14.6), VULDE (average age 23.8) and HBA (average age 29.5) (15, 16).

**Methylation data**

DNA methylation in all saliva samples (CELSPAC: YA) was assessed using the Ilumina EPIC Platform. Briefly, raw Illumina microarray data were processed using R package ChAMP (16).
Beta mixture quantile normalization (BMIQ) method was used to adjust the beta-values of type II design probes into a statistical distribution characteristic of type I probes. The analysis of epigenetic changes using epigenome-wide association studies (EWAS) in saliva samples can be applicable for assessment of potential relationship with external exposure. Saliva can be used as an alternative to blood in DNA methylation analysis for cohort studies (17).

**Funding and acknowledgement**

Authors thank RECETOX Research Infrastructure (No LM2023069) financed by the Ministry of Education, Youth and Sports, and the Operational Programme Research, Development and Education (the CETOCOEN EXCELLENCE project No. CZ.02.1.01/0.0/0.0/17_043/0009632 and Cetocoen Plus CZ.02.1.01/0.0/0.0/15_003/0000469) for supportive background. This work has been supported from project the Horizon Europe program No 101096888 (DISCERN). The laboratory part of this work was supported by the project BBMRI.cz (LM2023033). This work was supported from the European Union’s Horizon 2020 research and innovation program under grant agreement No 857560 (CETOCOEN Excellence). This publication reflects only the author's view, and the European Commission is not responsible for any use that may be made of the information it contains. This work was also supported from OP JAK project AGEING-CZ (no. CZ.02.01.01/00/23_025/0008743), co-funded by EU. We thank Ondřej Mikeš for his help with the preparation of exposure geocoding and exposure assessment part. We also thank Jana Klánová (PI), Klára Marečková (data selected sub-cohorts), STOMA research team led by Lydie Izakovicova Holla, the Population Studies Research Infrastructure, Lenka Andrýsková and her team, Pavel Piler, Ludmila Šebejová, and laboratory personnel involved on the CELSPAC: YA cohorts. We are also grateful to all participants who took part in this study, and the whole ELSPAC-CZ team led by Lubomir Kukla, which includes interviewers, computer and laboratory technicians, clerical workers, research scientists, volunteers, managers and nurses.

**Ethical approval**

The CELSPAC: YA study (Central European Longitudinal Studies of Parents and Children: Young Adults) was approved by the ELSPAC Ethics Committee, Masaryk University, Brno, the Czech Republic (No. ELSPAC/EK/2/2019, date 13/03/2019). All data and samples are available for secondary use in further research and development purposes. The VULDE study (Biomarkers and Underlying Mechanisms of Vulnerability to Depression) was approved by the ELSPAC Ethics Committee, Masaryk University, Brno, the Czech Republic (No. ELSPAC/EK/2/2014, date 17/10/2014). The HBA study (Biological aging of young adults) was approved by the ELSPAC Ethics Committee, Masaryk University, Brno, the Czech Republic (No. ELSPAC/EK/2/2020, date 08/06/2020).

**NTR**

**Cohort description**

The Netherlands Twin Register is a population-based cohort of over 200,000 people from across the Netherlands. It consists of twin-families, i.e. twins, their parents, spouses and siblings aged between 0 and 99 years at recruitment and started around 1987 with new–born twins and adolescent and adult twins. Full details have been reported previously (18). For the current paper, we analyzed DNA methylation measured in whole blood collected in the NTR-Biobank study (19-21). Good quality whole blood DNA methylation data (Illumina 450k array) were available for 3087 samples from 3055 individuals, including monozygotic and dizygotic twins, parents of twins, siblings of twins and spouses of twins. The current analysis included individuals with complete DNA and complete information on covariates (N= 2981 samples).

Informed consent was obtained from all participants. The study was approved by the Central Ethics Committee on Research Involving Human Subjects of the VU University Medical Centre, Amsterdam, an Institutional Review Board certified by the U.S. Office of Human Research Protections (IRB number IRB00002991 under Federal-wide Assurance- FWA00017598; IRB/institute codes, NTR 003-181, approval date 04-12-2003 (survey 7); 2008-244, approval date 01-12-2008 (survey 8); 2003-180, approval date 23-12-2003 (biobank)).

**Methylation data**

DNA methylation was assessed with the Infinium HumanMethylation450 BeadChip Kit (Illumina, San Diego, CA, USA) by the Human Genotyping facility (HugeF) of ErasmusMC, the Netherlands (<http://www.glimdna.org/>) as part of the Biobank-based Integrative Omics Study (BIOS) consortium (5). DNA methylation measurements have been described previously (21, 22). A number of sample- and probe-level quality checks and sample identity checks were performed, as described in detail previously (21). In short, sample-level QC was performed using MethylAid (23). Probes were set to be missing in a sample if they had an intensity value of exactly zero, or a detection p > .01, or a bead count of < 3. Probes failing in >5% of samples based on the above criteria were excluded from all samples. Additional exclusions included sex chromosomes, SNP-containing probes, and ambiguous mapping probes (24, 25). The data were normalized using functional normalization, resulting in 411,169 final methylation sites after QC.

In NTR, generalized estimation equation (GEE) models fitted with the R package geepack (26) were used to test the association between PM2.5 and DNA methylation. The following settings were used: Gaussian link function (for continuous data), 100 iterations, and the “exchangeable” option to account for the correlation structure within families.

**Funding and acknowledgement**

We warmly thank all twin families of the Netherlands Twin Register who make this research possible. DNA methylation data were generated at the Human Genomics Facility (HuGe-F) at ErasmusMC Rotterdam. Gene expression data were generated at the Rutgers University and DNA Repository.

We acknowledge funding from the Netherlands Organization for Scientific Research (NWO): Biobanking and Biomolecular Research Infrastructure (BBMRI-NL, NWO 184.033.111) and the BBRMI-NL-financed BIOS Consortium (NWO 184.021.007), NWO; 480–15-001/674; 480–04-004; 400–05-717, the European Science Council (ERC) Genetics of Mental Illness (ERC Advanced, 230374); Developmental trajectories of psychopathology (NIMH 1RC2 MH089995). JvD was supported by NWO Large Scale infrastructures, X-omics (184.034.019).

**Estonian Biobank**

**Cohort description**

The Estonian Biobank is a volunteer-based biobank in Estonia, covering the whole country (27, 28). Currently, the biobank consists of over 210,000 participants, who have all signed a broad informed consent for at joining to allow the use of their sample and data in further research. Information from the national registries is regularly linked with Estonian Biobank, allowing diagnoses from both primary care and hospitals, death registry, cancer registry and other sources to be used to enrich the Estonian Biobank data. All Estonian Biobank participants are genotyped, and subsets have various omics measurements, such as metabolomics, transcriptomics, epigenomics. The activities of the EstBB are regulated by the Human Genes Research Act, which was adopted in 2000 specifically for the operations of the EstBB. All participants have signed an informed consent form during recruitment. Individual level data analysis in the EstBB was carried out under ethical approvals 1.1-12/3435 (08.12.2020), 1.1-12/1021 (13.04.2021), 1.1-12/1021 (14.12.2021), 1.1-1/3452 (20.10. 2022), 1.1-12/1086 (13.03.2023), 1.1-12/4367 (07.12.2023) from the Estonian Committee on Bioethics and Human Research (Estonian Ministry of Social Affairs), using data according to release application 6-7/GI/10844 from the Estonian Biobank.

**Methylation data**

Methylation was measured with Illumina HumanMethylation450 BeadChip. The methylation dataset used is a subsample of CTG project consisting of random population-based controls (not selected based on any phenotype). The final dataset consisted of 304 samples, 473864 CpG sites (CpG sites on X and Y chromosome are excluded). Proportion of cell types (CD8T, CD4T, NK, Bcell, Mono, Gran) was estimated using Houseman method implemented in minfi (estimateCellCounts function). Normalization of the DNA methylation data was done using the CPACOR (incorporating Control Probe Adjustment and reduction of global CORrelation by (29) method. Beta values were converted to M values.

**Funding and acknowledgement**

The methylation dataset has been created as part of the CTG project led by Prof. Lili Milani. We would like to thank Dr. Silva Kasela for QC and normalisation of the methylation dataset.

The research was conducted using the Estonian Center of Genomics/Roadmap II funded by the Estonian Research Council (project number TT17). Data analysis of EstBB was carried out in part in the High-Performance Computing Center of University of Tartu.

**SAPALDIA**

**Cohort description**

The SAPALDIA study was initiated in 1991 recruiting 9651 population-representative subjects of age 18-60 years (30, 31). Three follow-ups have been completed: 8047 out of the 9651 subjects participated in SAPALDIA2 in 2001/2002 and 6088 in SAPALDIA3 in 2010/2011. Data and blood samples collected in SAPALDIA3 are used in the current study. All participants provided prior written informed consent. The study protocol of SAPALDIA was approved by the Swiss Academy of Medical Sciences and the regional committees for each study center.

**Methylation data**

Genome-wide DNA methylation was measured in the peripheral blood samples using Illumina Infinium HumanMethylation450 BeadChip (Illumina, San Diego, CA, USA) following manufacturer’s protocol (32-34). DNA methylation data were processed using R package “minfi” (35). Samples with sex mismatch were excluded. Background and dye-bias correction was performed using Noob (normal-exponential out-of-band) procedure (36). Beta-values were computed as the ratio of methylated intensity over total intensity with offset 100. Beta-values with detection p-value > 10^-16^ were set to missing. Probes with call rate < 0.95 were excluded. No samples were excluded at the predefined call rate cutoff < 0.95. Illumina probe design bias was corrected by applying beta-mixture quantile normalization (BMIQ) (37). We only used autosomal chromosome probes in this study.

**Funding and acknowledgement**

SAPALDIA was supported by the Swiss National Science Foundation (grants no 33CS30-177506/1, 33CS30-148470/1&2, 33CSCO-134276/1, 33CSCO-108796, , 324730_135673, 3247BO-104283, 3247BO-104288, 3247BO-104284, 3247-065896, 3100-059302, 3200-052720, 3200-042532, 4026-028099, PMPDP3_129021/1, PMPDP3_141671/1), the Federal Office for the Environment, the Federal Office of Public Health, the Federal Office of Roads and Transport, the canton's government of Aargau, Basel-Stadt, Basel-Land, Geneva, Luzern, Ticino, Valais, and Zürich, the Swiss Lung League, the canton's Lung League of Basel Stadt/ Basel Landschaft, Geneva, Ticino, Valais, Graubünden and Zurich, Stiftung ehemals Bündner Heilstätten, SUVA, Freiwillige Akademische Gesellschaft, UBS Wealth Foundation, Talecris Biotherapeutics GmbH, Abbott Diagnostics, Klinik Barmelweid, Hirslanden Klinik Aarau, European Commission 018996 (GABRIEL), Wellcome Trust WT 084703MA, Exposomics EC FP7 grant (Grant agreement No: 308610).

The study could not have been done without the help of the study participants, technical and administrative support and the medical teams and field workers at the local study sites.

Study directorate: NM Probst-Hensch (PI; e/g); D Stolz (p), C Schindler (s), N Künzli (e/exp).

Scientific team: JC Barthélémy (c), W Berger (g), R Bettschart (p), A Bircher (a), C Brombach (n), PO Bridevaux (p), L Burdet (p), Felber Dietrich D (e), T Sigrist (p), U Frey (pd), MW Gerbase (p), D Gold (e), E de Groot (c), W Karrer (p), F Kronenberg (g), B Martin (pa), A Mehta (e), D Miedinger (o), M Pons (p), F Roche (c), T Rothe (p), P Schmid-Grendelmeyer (a), A Schmidt-Trucksäss (pa), J Schwartz (e), A Turk (p), A von Eckardstein (cc), E Zemp Stutz (e).

Scientific team at coordinating centers: M Adam (e), I Aguilera (exp), A Beckmeyer-Borowko (e), S Brunner (s), D Carballo (c), S Caviezel (pa), I Curjuric (e), A Di Pascale (s), J Dratva (e), R Ducret (s), E Dupuis Lozeron (s), M Eeftens (exp), I Eze (e), E Fischer (g), M Foraster (e), M Germond (s), L Grize (s), S Hansen (e), A Hensel (s), M Imboden (g), A Ineichen (exp), A Jeong (g), D Keidel (s), A Kumar (g), N Maire (s), A Mehta (e), R Meier (exp), E Schaffner (s), T Schikowski (e), M Tsai (exp)

(a) allergology, (c) cardiology, (cc) clinical chemistry, (e) epidemiology, (exp) exposure, (g) genetic and molecular biology, (m) meteorology, (n) nutrition, (o) occupational health, (p) pneumology, (pa) physical activity, (pd) pediatrics, (s) statistics

**Ethical approval**

All procedures in this cohort study were conducted in accordance with the World Medical Association’s Declaration of Helsinki and Declaration of Taipei. Written informed consent was obtained from the study participants prior to health examination and blood sample collection. The study protocols of this multi-centric long-term study with baseline and follow-up assessments were approved by Swiss national overarching ethics committees and by regional cantonal ethics committees for each time point of data collection. The SAPALDIA cohort study was approved by the ethics committee of the medical faculty of the University of Lausanne, Switzerland, for the baseline examination (SAPALDIA1) in 1989; and by the Supra-regional Ethics Committee for Clinical Research (UREK approval N° 123/00) of the Swiss Academy of Medical Sciences for the second examination (SAPALDIA2) in September 2001; and given the multi-centric design of the long-term cohort, by multiple cantonal ethics committees for the third examination (SAPALDIA3) in 2009 (ethics committee of the Department of Health and Social Affairs of Aargau, approval N° 2009/056, ethics committee of both Basel, approval N° 219/09, cantonal ethics committee of Zurich, approval N° 52/09, departmental ethics committee for Internal Medicine and Community Medicine of Geneva, approval N° 09-174, cantonal commission for Medical Ethics of Valais, approval N°033/09, cantonal Commission for Ethics in Human Research of Vaud, approval N° 200/09, cantonal Ethics Committee of Ticino, approval N° CE2276). The fourth examination (SAPALDIA4) in 2015-2018 the lead ethics commission was the Commission for Ethics of Northwestern and Central Region of Switzerland (2015-237 and PB_2016-02348); the regional study centers were approved by the Ethics committees of the cantons of Geneva, Ticino, and Vaud under the lead of the Commission for Ethics of Northwestern and Central Region of Switzerland. The fifth examination (SAPALDIA5) in conducted during the years 2019-2022 was approved by the lead ethics commission Commission for Ethics of Northwestern and Central Region of Switzerland (2019-01849).

**KORA**

**Cohort description**

KORA (Cooperative Health Research in the Region of Augsburg) is a regional research platform for population-based surveys and subsequent follow-up studies in the fields of epidemiology, health economics, and health care research (https://www.helmholtz-munich.de/en/epi/cohort/kora). The KORA study was initiated and financed by the Helmholtz Zentrum München – German Research Center for Environmental Health, which is funded by the German Federal Ministry of Education and Research (BMBF) and by the State of Bavaria. Data collection in the KORA study is done in cooperation with the University Hospital of Augsburg. The data in these analyses are taken from the KORA F4 which is a follow-up examination of KORA S4. The KORA S4 study comprises 4,261 participants (25–74 years) who were randomly selected during 1999 and 2001 from population registries in the city of Augsburg and the two adjacent administrative districts. In the first follow-up examination (F4) 3,080 participants (72%) were re-examined after seven years. Methylation measurements were available in 1799 participants of KORA F4.

**Methylation data**

The methylation data were generated in one batch using the Illumina HumanMethylation 450 BeadChip. The preprocessing of the methylation data was conducted following the general outline of the CPACOR processing pipeline for quality control by using minfi2 package (38). The data were processed using R (v4.3.0).

Preprocessing in brief:

- Sex prediction: no individuals with a predicted sex different from the sex given at the time of the interview.

- Quality control on raw intensities: 1 individual failed the QC (cut-off 9) and was removed.

- Detection p-value filter: Observations whose detection p-values were greater than 0.01 were set to missing.

- Sample call rate filter: Samples with greater than 5% missing values (testing the autosomes only) were removed. This led to the exclusion of 72 individuals.

- CpG call rate filter: CpGs with greater than 10% missing values on the autosomes were removed (N= 14541).

- CpG probe exclusion: Manifest HM450.hg19.manifest.pop.tsv.gz was used, based on (39). This yields 59186 CpGs to exclude.

- Quantile normalization: it was performed separately on the signal intensities divided into the 6 probe types: type II red, type II green, type I green unmethylated, type I green methylated, type I red unmethylated, type I red methylated (38). The quantile normalized intensities were then used to generate methylation beta values, a measure from 0 to 1 indicating what percent of the cells were methylated at this locus. QN was performed using the R package limma v3.56.2 (40).

- Cell type heterogeneity: the estimates of white blood cell counts were conducted using the R package EpiDISH. We obtain estimates for 12 cell types ("Baso", "Bmem", "Bnv", "CD4Tmem", "CD4Tnv", "CD8Tmem", "CDT8nv", "Eos", "Mono", "Neu", "NK", "Treg").

- Technical covariates: We calculated the principal components (PCs) of all the non-negative control probes, as per the CPACOR pipeline, to account for technical effects.

- Sample count summary:

1799 individuals were measured. A total of 72 were removed due to quality control: these all failed the detection rate threshold, and 1 additionally failed the median intensity step. This leaves 1727 individuals passing quality control. Beta-values where then transformed in M-values (41) for the analyses.

**Funding & Acknowledgement**

The KORA study was initiated and financed by the Helmholtz Zentrum München – German Research Center for Environmental Health, which is funded by the German Federal Ministry of Education and Research (BMBF) and by the State of Bavaria. Data collection in the KORA study is done in cooperation with the University Hospital of Augsburg. The ethics committee number for the KORA F4 follow-up study is No. 06068. We thank all participants for their long-term commitment to the KORA study, the staff for data collection and research data management and the members of the KORA Study Group (https://www.helmholtz-munich.de/en/epi/cohort/kora) who are responsible for the design and conduct of the study.

1. **Supplementary Results**
   1. **Descriptive statistics of exposure variables**

**Table S1:** Descriptive statistics of the different exposures for each cohort, grouped by life-stage.

| **Cohort** | **Exposure** | **n** | **Miss.** | **mean** | **sd** | **min** | **Q0.25** | **Q0.5** | **Q0.75** | **max** | **IQR** |
| --- | --- | --- | --- | --- | --- | --- | --- | --- | --- | --- | --- |
| KORA F4 | PM2.5_annual | 1722 |  | 13.7 | 1.8 | 9.5 | 12.0 | 14.0 | 15.4 | 18.1 | 3.4 |
|  | NO_2__annual | 1722 |  | 27.5 | 9.1 | 9.2 | 20.2 | 26.2 | 33.5 | 56.0 | 13.4 |
|  | PM2.5_monthly | 1722 |  | 13.9 | 4.2 | 3.2 | 11.1 | 13.4 | 16.9 | 27.1 | 5.8 |
|  | NO_2__monthly | 1722 |  | 27.0 | 9.6 | 2.3 | 19.4 | 26.6 | 33.8 | 54.1 | 14.5 |
|  | O_3_ | 1722 |  | 87.8 | 3.4 | 78.1 | 85.5 | 87.8 | 89.8 | 98.7 | 4.3 |
|  | LAN | 1722 |  | 45.2 | 15.6 | 8.0 | 35.0 | 53.0 | 58.0 | 59.0 | 23.0 |
|  | MSAVI | 1722 |  | 0.7 | 0.1 | 0.4 | 0.6 | 0.7 | 0.7 | 0.8 | 0.1 |
|  | Urbanicity | 1722 |  | 25.4 | 17.8 | 0.1 | 9.3 | 22.7 | 41.0 | 70.4 | 31.8 |
| Sapaldia | PM2.5_annual | 969 |  | 14.3 | 4.2 | 3.6 | 12.2 | 14.7 | 17.2 | 24.2 | 5.0 |
|  | NO_2__annual | 969 |  | 23.4 | 6.7 | 8.8 | 18.5 | 22.2 | 27.3 | 68.3 | 8.8 |
|  | PM2.5_monthly | 969 |  | 15.8 | 6.9 | 2.4 | 10.9 | 14.1 | 18.8 | 43.5 | 7.9 |
|  | NO_2__monthly | 969 |  | 24.2 | 11.1 | 1.8 | 15.3 | 22.2 | 32.2 | 66.2 | 16.8 |
|  | O_3_ | 969 |  | 91.6 | 5.1 | 76.0 | 87.5 | 92.3 | 94.7 | 110.8 | 7.2 |
|  | LAN | 969 |  | 48.5 | 9.4 | 9.0 | 43.0 | 50.0 | 57.0 | 59.0 | 14.0 |
|  | MSAVI | 969 |  | 0.7 | 0.1 | 0.3 | 0.6 | 0.7 | 0.8 | 0.9 | 0.1 |
|  | Urbanicity | 969 |  | 27.6 | 31.3 | 0.2 | 7.9 | 17.6 | 29.7 | 172.9 | 21.7 |
| Estonian Biobank | PM2.5_annual | 303 |  | 7.8 | 1.2 | 5.7 | 6.9 | 7.6 | 8.7 | 11.6 | 1.7 |
|  | NO_2__annual | 303 |  | 8.6 | 5.5 | 0.0 | 4.1 | 7.7 | 11.6 | 26.2 | 7.5 |
|  | PM2.5_monthly | 303 |  | 7.9 | 3.5 | 3.2 | 5.7 | 6.9 | 9.1 | 24.2 | 3.4 |
|  | NO_2__monthly | 303 |  | 9.5 | 6.6 | 0.0 | 4.5 | 9.3 | 13.5 | 34.5 | 9.1 |
|  | O_3_ | 303 |  | 73.7 | 3.0 | 62.6 | 71.8 | 73.8 | 75.8 | 82.2 | 3.9 |
|  | LAN | 303 |  | 43.7 | 18.0 | 8.0 | 30.8 | 52.0 | 59.0 | 59.0 | 28.3 |
|  | MSAVI | 303 |  | 0.7 | 0.1 | 0.4 | 0.7 | 0.7 | 0.8 | 0.9 | 0.1 |
|  | Urbanicity | 303 |  | 15.0 | 19.7 | 0.0 | 0.5 | 7.4 | 20.5 | 103.7 | 20.0 |
| NTR | PM2.5_annual | 2891 |  | 16.4 | 1.5 | 4.1 | 15.6 | 16.4 | 17.3 | 20.9 | 1.7 |
|  | NO_2__annual | 2891 |  | 28.2 | 6.6 | 10.6 | 23.7 | 27.8 | 32.7 | 49.8 | 9.0 |
|  | NO_2__monthly | 2891 |  | 29.4 | 8.7 | 3.2 | 23.0 | 29.2 | 35.4 | 58.6 | 12.4 |
|  | O_3_ | 2891 |  | 74.0 | 6.2 | 55.3 | 70.0 | 73.5 | 77.4 | 88.2 | 7.4 |
|  | LAN | 2891 |  | 50.3 | 11.4 | 10.0 | 46.0 | 55.0 | 58.0 | 59.0 | 12.0 |
|  | MSAVI | 2891 |  | 0.6 | 0.1 | 0.0 | 0.6 | 0.7 | 0.7 | 0.9 | 0.1 |
|  | Urbanicity | 2891 |  | 31.9 | 23.3 | 0.1 | 14.4 | 26.6 | 44.1 | 128.6 | 29.7 |
| PIAMA  (adolescents) | PM2.5_annual | 611 |  | 13.2 | 1.4 | 9.5 | 12.3 | 13.4 | 14.4 | 16.0 | 2.1 |
|  | NO_2__annual | 611 |  | 23.6 | 5.6 | 9.2 | 19.5 | 23.9 | 27.6 | 41.3 | 8.2 |
|  | PM2.5_monthly | 611 |  | 12.9 | 3.8 | 6.8 | 10.3 | 11.6 | 15.3 | 24.0 | 4.9 |
|  | NO_2__monthly | 611 |  | 22.4 | 6.4 | 4.6 | 18.0 | 22.4 | 27.2 | 37.8 | 9.2 |
|  | O_3_ | 611 |  | 76.7 | 1.8 | 69.1 | 75.9 | 76.9 | 77.9 | 80.7 | 2.1 |
|  | LAN | 611 |  | 47.6 | 12.5 | 9.0 | 45.0 | 53.0 | 56.0 | 59.0 | 11.0 |
|  | MSAVI | 610 | 1 | 0.7 | 0.1 | 0.5 | 0.7 | 0.7 | 0.8 | 0.9 | 0.1 |
|  | Urbanicity | 611 |  | 25.2 | 16.5 | 0.1 | 11.5 | 22.1 | 37.2 | 88.7 | 25.7 |
| BAMSE  (adolescents) | PM2.5_annual | 267 |  | 6.5 | 1.1 | 4.0 | 5.9 | 6.6 | 7.4 | 8.8 | 1.5 |
|  | NO_2__annual | 267 |  | 16.2 | 5.7 | 5.2 | 12.4 | 14.6 | 18.1 | 39.9 | 5.7 |
|  | PM2.5_monthly | 267 |  | 6.5 | 2.0 | 0.7 | 5.4 | 6.5 | 7.5 | 11.6 | 2.1 |
|  | NO_2__monthly | 267 |  | 19.0 | 6.9 | 5.8 | 13.7 | 17.9 | 23.9 | 45.1 | 10.2 |
|  | O_3_ | 267 |  | 74.1 | 3.1 | 62.1 | 73.3 | 75.0 | 75.8 | 79.0 | 2.6 |
|  | LAN | 267 |  | 59.7 | 7.5 | 12.0 | 61.0 | 62.0 | 62.0 | 62.0 | 1.0 |
|  | MSAVI | 267 |  | 0.6 | 0.1 | 0.3 | 0.6 | 0.7 | 0.7 | 0.8 | 0.1 |
|  | Urbanicity | 267 |  | 47.3 | 35.3 | 0.3 | 19.6 | 27.4 | 54.9 | 216.6 | 35.3 |
| LISA (10 years) | PM2.5_annual | 226 |  | 12.8 | 1.5 | 8.9 | 11.6 | 13.3 | 13.8 | 16.7 | 2.2 |
|  | NO_2__annual | 226 |  | 28.7 | 9.9 | 8.9 | 21.7 | 27.4 | 34.5 | 58.6 | 12.7 |
|  | PM2.5_monthly | 226 |  | 12.5 | 3.3 | 7.8 | 10.7 | 12.0 | 13.2 | 39.0 | 2.5 |
|  | NO_2__monthly | 226 |  | 26.8 | 10.1 | 6.0 | 19.7 | 26.6 | 33.8 | 53.2 | 14.1 |
|  | O_3_ | 226 |  | 84.0 | 3.7 | 73.9 | 81.9 | 84.3 | 86.5 | 92.5 | 4.7 |
|  | LAN | 226 |  | 48.3 | 13.7 | 10.0 | 44.0 | 55.0 | 58.0 | 59.0 | 14.0 |
|  | MSAVI | 226 |  | 0.7 | 0.1 | 0.3 | 0.6 | 0.7 | 0.7 | 0.8 | 0.1 |
|  | Urbanicity | 226 |  | 37.0 | 27.2 | 0.5 | 13.8 | 28.8 | 62.4 | 104.0 | 48.6 |
| PIAMA (8 years) | PM2.5_annual | 205 |  | 15.6 | 1.6 | 12.8 | 13.9 | 15.8 | 16.9 | 18.1 | 3.0 |
|  | NO_2__annual | 205 |  | 27.0 | 5.6 | 14.5 | 23.8 | 27.2 | 31.0 | 41.0 | 7.1 |
|  | NO2_monthly | 205 |  | 31.0 | 9.0 | 11.6 | 26.0 | 30.7 | 37.0 | 54.0 | 11.0 |
|  | O_3_ | 205 |  | 72.1 | 4.0 | 62.8 | 68.8 | 72.0 | 75.0 | 85.4 | 6.2 |
|  | LAN | 205 |  | 50.9 | 11.4 | 10.0 | 48.5 | 56.0 | 58.0 | 59.0 | 9.5 |
|  | MSAVI | 205 |  | 0.7 | 0.1 | 0.5 | 0.6 | 0.7 | 0.7 | 0.8 | 0.1 |
|  | Urbanicity | 205 |  | 27.8 | 16.6 | 0.8 | 14.2 | 25.1 | 38.3 | 92.0 | 24.1 |
| BAMSE EpiGene | PM2.5_annual | 375 |  | 9.7 | 1.0 | 6.8 | 9.1 | 9.8 | 10.4 | 12.2 | 1.3 |
|  | NO_2__annual | 375 |  | 19.0 | 6.1 | 5.5 | 14.7 | 17.9 | 22.6 | 34.1 | 7.9 |
|  | NO_2__monthly | 264 | 111 | 21.0 | 7.1 | 2.9 | 16.3 | 20.1 | 25.1 | 41.8 | 8.8 |
|  | O_3_ | 375 |  | 78.0 | 5.5 | 62.5 | 74.8 | 78.6 | 82.2 | 87.1 | 7.4 |
|  | LAN | 375 |  | 58.3 | 7.3 | 10.0 | 59.0 | 60.0 | 61.0 | 61.0 | 2.0 |
|  | MSAVI | 375 |  | 0.6 | 0.1 | 0.3 | 0.6 | 0.7 | 0.7 | 0.9 | 0.1 |
|  | Urbanicity | 255 | 120 | 48.8 | 50.4 | 0.2 | 19.6 | 28.8 | 57.9 | 222.8 | 38.3 |
| BAMSE MeDALL | PM2.5_annual | 264 |  | 9.8 | 0.9 | 4.1 | 9.3 | 9.9 | 10.4 | 12.3 | 1.1 |
|  | NO_2__annual | 264 |  | 19.0 | 6.3 | 5.9 | 14.5 | 16.9 | 22.3 | 40.4 | 7.8 |
|  | NO_2__monthly | 264 |  | 20.9 | 7.0 | 3.7 | 16.1 | 19.9 | 24.2 | 41.7 | 8.1 |
|  | O_3_ | 264 |  | 78.3 | 5.8 | 62.1 | 74.0 | 79.4 | 83.0 | 86.9 | 8.9 |
|  | LAN | 264 |  | 58.8 | 5.0 | 10.0 | 59.0 | 60.0 | 61.0 | 61.0 | 2.0 |
|  | MSAVI | 264 |  | 0.6 | 0.1 | 0.3 | 0.6 | 0.7 | 0.7 | 0.8 | 0.1 |
|  | Urbanicity | 264 |  | 49.8 | 53.3 | 0.8 | 19.7 | 25.8 | 56.1 | 216.6 | 36.4 |
| LISA 6years | PM2.5_annual | 234 |  | 15.0 | 1.4 | 10.9 | 14.1 | 15.5 | 16.0 | 17.8 | 1.9 |
|  | NO_2__annual | 234 |  | 28.8 | 9.6 | 10.5 | 21.6 | 27.9 | 35.0 | 53.3 | 13.4 |
|  | NO_2__monthly | 234 |  | 28.7 | 10.4 | 6.4 | 21.5 | 28.8 | 35.3 | 55.5 | 13.8 |
|  | O_3_ | 234 |  | 87.1 | 4.3 | 74.4 | 84.3 | 87.3 | 90.2 | 96.9 | 5.8 |
|  | LAN | 234 |  | 49.2 | 13.4 | 10.0 | 47.2 | 56.0 | 58.0 | 59.0 | 10.8 |
|  | MSAVI | 234 |  | 0.6 | 0.1 | 0.3 | 0.6 | 0.7 | 0.7 | 0.8 | 0.1 |
|  | Urbanicity | 234 |  | 38.8 | 27.0 | 0.5 | 14.0 | 31.4 | 64.7 | 104.0 | 50.7 |
| PIAMA  (4 years) | PM2.5_annual | 228 |  | 15.4 | 1.3 | 12.3 | 14.5 | 15.6 | 16.4 | 17.9 | 1.9 |
|  | NO_2__annual | 228 |  | 27.5 | 5.4 | 15.1 | 24.6 | 27.9 | 31.3 | 40.8 | 6.7 |
|  | NO_2__monthly | 228 |  | 32.5 | 8.9 | 12.7 | 25.3 | 32.9 | 39.3 | 51.6 | 14.0 |
|  | O_3_ | 228 |  | 71.3 | 3.5 | 60.6 | 69.0 | 71.5 | 74.0 | 79.4 | 5.0 |
|  | LAN | 224 | 4 | 48.6 | 13.1 | 9.0 | 42.0 | 54.0 | 58.0 | 61.0 | 16.0 |
|  | MSAVI | 228 |  | 0.7 | 0.1 | 0.5 | 0.6 | 0.7 | 0.7 | 0.8 | 0.1 |
|  | Urbanicity | 228 |  | 28.2 | 16.4 | 0.8 | 14.7 | 25.4 | 40.0 | 65.4 | 25.3 |
| BAMSE (4 years) | PM2.5_annual | 255 |  | 10.3 | 1.6 | 7.7 | 8.7 | 10.1 | 11.8 | 113.4 | 3.1 |
|  | NO_2__annual | 255 |  | 19.0 | 6.1 | 5.2 | 14.3 | 17.1 | 24.2 | 38.9 | 9.9 |
|  | O_3_ | 255 |  | 71.1 | 5.0 | 56.9 | 67.7 | 72.7 | 74.8 | 78.7 | 7.1 |
|  | LAN | 255 |  | 60.9 | 4.0 | 18.0 | 61.0 | 62.0 | 62.0 | 62.0 | 1.0 |
|  | MSAVI | 255 |  | 0.6 | 0.1 | 0.3 | 0.5 | 0.6 | 0.6 | 0.8 | 0.1 |
|  | Urbanicity | 255 |  | 48.1 | 36.2 | 0.8 | 19.8 | 25.8 | 56.0 | 212.0 | 36.2 |

PM2.5: particulate matter with aerodynamic diameter < 2.5 μm; NO_2_: nitrogen dioxide; O_3_: ozone; LAN: light at night; MSAVI: modified soil adjusted index. Abbreviations: n: number of observations; Miss.: missing values; sd: standard deviation; min: minimum; Q0.25: 25th percentile; Q0.5: 50th percentile (median); Q0.75: 75th percentile; max: maximum; IQR: interquartile range.

**Figure S1:** The map of Europe (A) illustrates the geographic location of each cohort, while the adjacent bar plot (B) shows the average age per cohort. The distribution of exposure levels across cohorts is represented by histogram (C): mean values are reported on the y axis with confidence interval bars included.


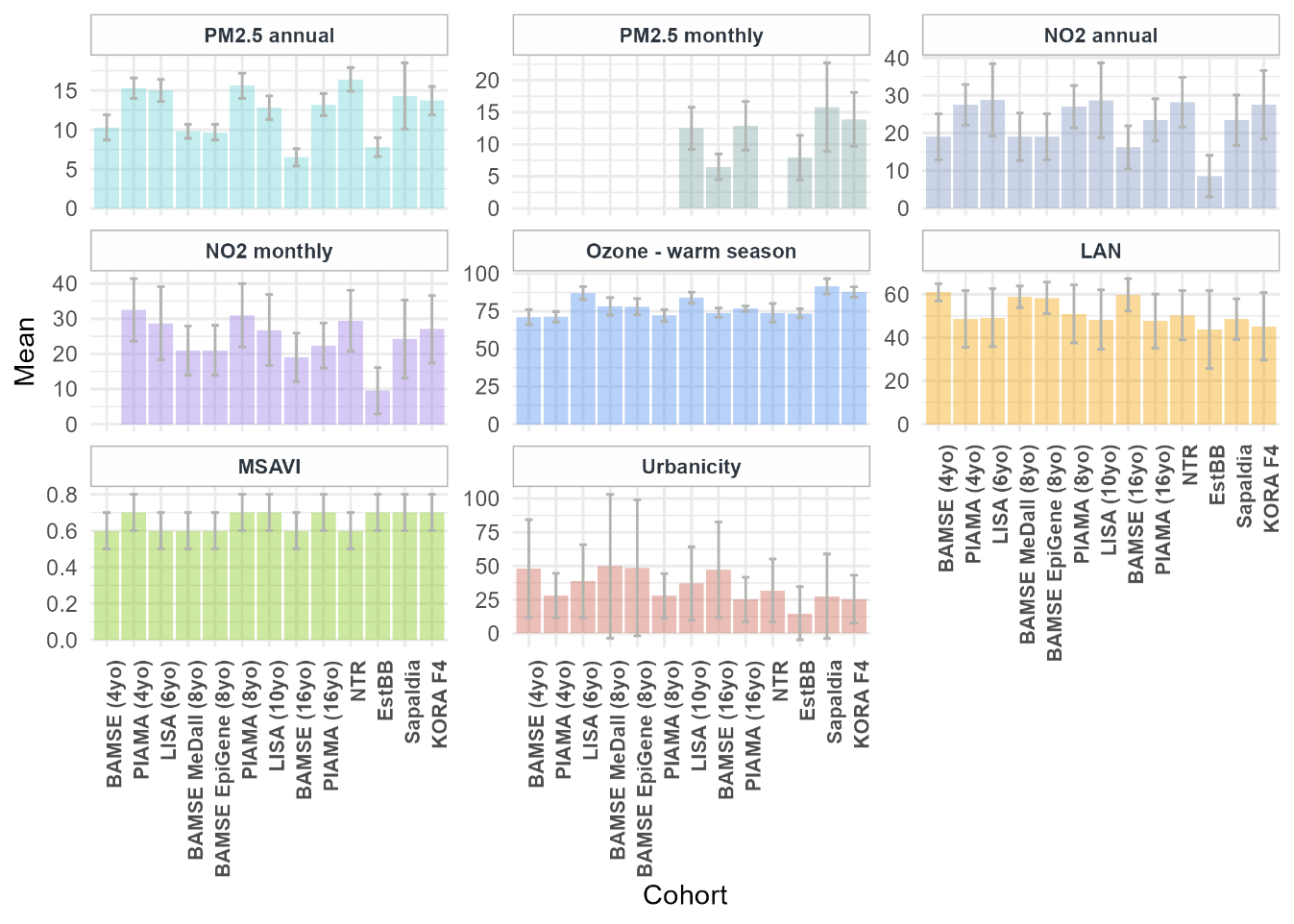

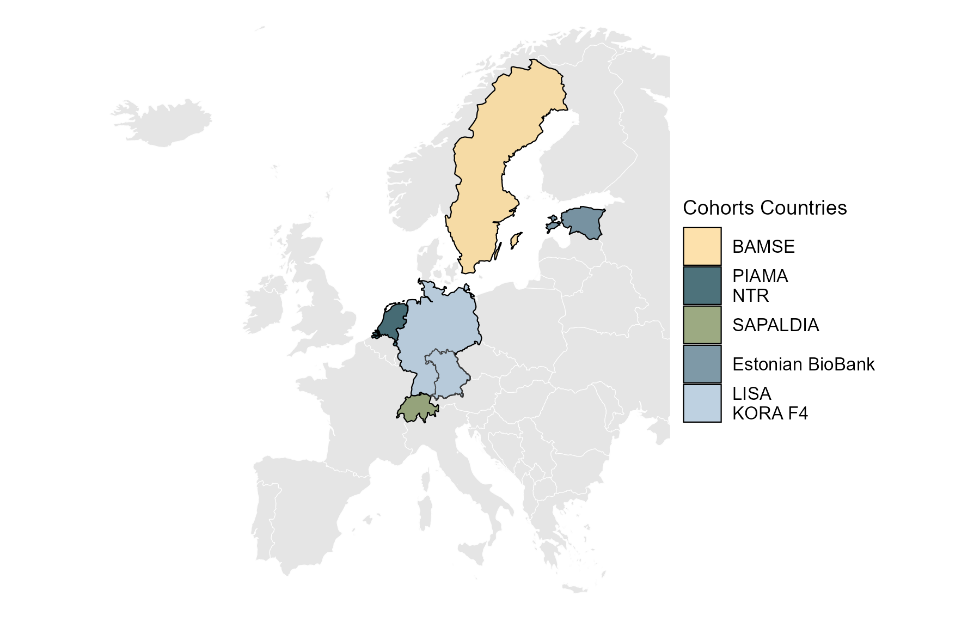


●

●


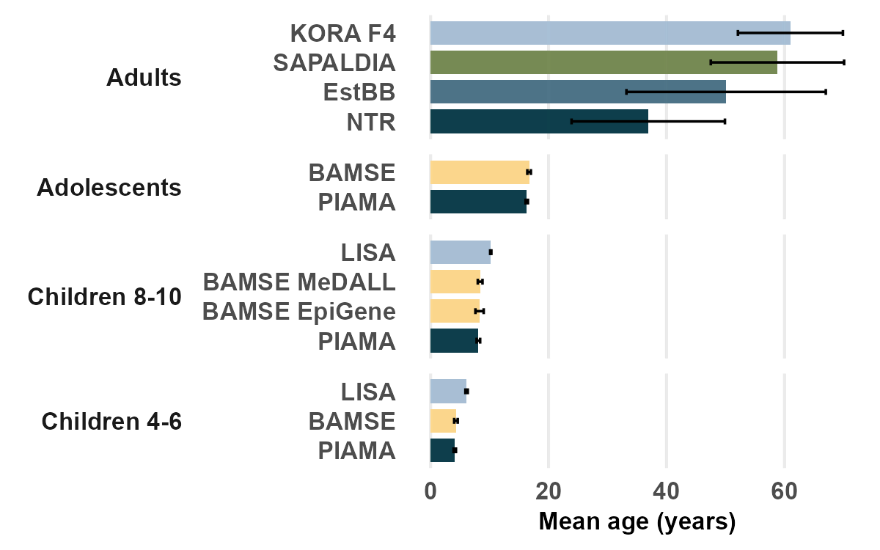


**A**

**B**

**C**

- 1. **Meta-analysis of epigenome-wide association studies**

**Table S2:** Genomic inflation factor and the number of common probes included in the meta-analysis.

| **Life-stage** | **Cohorts** | **PM2.5_annual** | | **PM2.5_monthly** | | **NO_2__annual** | | **NO_2__monthly** | | **O_3_** | | **LAN** | | **MSAVI** | | **Urbanicity** | |
| --- | --- | --- | --- | --- | --- | --- | --- | --- | --- | --- | --- | --- | --- | --- | --- | --- | --- |
|  |  | **λ** | **N probe** | **λ** | **N probe** | **λ** | **N probe** | **λ** | **N probe** | **λ** | **N probe** | **λ** | **N probe** | **λ** | **N probe** | **λ** | **N probe** |
| Adult | KORAF4 | 0.908 | 374185 | 0.905 | 377279 | 0.909 | 374516 | 0.918 | 374066 | 0.907 | 374566 | 0.904 | 374707 | 0.906 | 373935 | 0.908 | 374437 |
|  | SAPALDIA | 1.004 |  | 1.001 |  | 1.000 |  | 1.000 |  | 0.996 |  | 0.999 |  | 1.004 |  | 1.005 |  |
|  | EstBB | 0.977 |  | 0.98 |  | 0.984 |  | 0.99 |  | 0.973 |  | 0.986 |  | 0.979 |  | 0.992 |  |
|  | NTR | 1.006 |  |  |  | 1.006 |  | 0.996 |  | 1.005 |  | 0.993 |  | 1.007 |  | 0.985 |  |
| Adolescents | PIAMA 16y | 1.021 | 433341 | 1.021 | 433299 | 1.013 | 433385 | 1.025 | 433516 | 1.02 | 433407 | 1.011 | 433264 | 1.006 | 433231 | 1.023 | 433354 |
|  | BAMSE 16 y | 1.017 |  | 1.013 |  | 1.021 |  | 1.014 |  | 1.031 |  | 1.000 |  | 0.998 |  | 1.019 |  |
| Children (8-10 y) | LISA 10y | 0.994 | 360394 | 1.129 | - | 1.033 | 360603 | 1.107 | 360477 | 1.053 | 360304 | 0.98 | 360519 | 1.023 | 360511 | 0.951 | 360370 |
|  | PIAMA 8y | 1.016 |  |  |  | 1.041 |  | 0.993 |  | 1.023 |  | 1.019 |  | 1.014 |  | 1.032 |  |
|  | BAMSE 8 y Epigene | 1.024 |  |  |  | 1.015 |  | 1.017 |  | 1.007 |  | 1.000 |  | 1.009 |  | 1.018 |  |
|  | BAMSE 8y MeDall | 1.041 |  |  |  | 1.02 |  | 1.097 |  | 1.023 |  | 0.986 |  | 0.991 |  | 1.015 |  |
| Children (4-6 y) | LISA 6y | 0.938 | 360931 |  | - | 0.922 | 361558 | 0.917 | 361804 | 1.039 | 361583 | 0.903 | 361011 | 1.07 | 361391 | 1.292* | 436827 |
|  | PIAMA 4y | 1.001 |  |  |  | 0.982 |  | 1.011 |  | 1.006 |  | 1.004 |  | 1.018 |  | 1.009 |  |
|  | BAMSE 4y | 1.008 |  |  |  | 1.032 |  | - |  | 1.021 |  | 1.056 |  | 0.968 |  | 1.000 |  |

PM2.5: particulate matter with aerodynamic diameter < 2.5 μm; NO_2_: nitrogen dioxide; O_3_: ozone; LAN: light at night; MSAVI: modified soil adjusted index.

*Studies excluded from the meta-analysis due to the genomic inflation factor exceeding the threshold.

**3.2.1 Sensitivity analysis in the adults age group: exclusion of the Estonian Biobank**

**Table S3:** Summary of meta-analysis results for different exposures in the adults age group, excluding the Estonian Biobank.

| **Exposure** | **MarkerName** | **β** | **StdErr** | **pvalue** | **FDR** | **Dir.** | **I^2^** | **chr** | **Relation to CpG Island** | **Mapped Gene** | **Gene Region** |
| --- | --- | --- | --- | --- | --- | --- | --- | --- | --- | --- | --- |
| PM2.5mon. | cg10112003 | -0.006 | 0.0011 | 2.16E-07 | 0.081 | -- | 0 | 3 | Island | ACTL6A | TSS200 |
| NO2ann. | cg17205313 | -0.002 | 0.0004 | 1.38E-07 | 0.052 | --- | 0 | 18 | OpenSea | TTC39C | 5'UTR;1stExon |
| MSAVI | cg17205313 | 0.167 | 0.0298 | 2.02E-08 | 0.008 | +++ | 3.9 | 18 | OpenSea | TTC39C | 5'UTR;1stExon |

β= coefficient estimate. StdErr = standard error. FDR = False Discovery Rate 10% (threshold). Dir. = Direction, summary of the effect direction for each study; CHR= chromosome number; Relation to CpG Island: the location of the CpG relative to the CpG island (Shore = 0-2 kb from island; Shelf = 2-4 kb from island; N = upstream (5’) of CpG island; S = downstream (3’) of CpG island); Gene region: (TSS200 = 0-200 bases upstream of the transcriptional start site (TSS); 5'UTR = Within the 5' untranslated region, between the TSS and the ATG start site; 1stExon = within the first exon of the transcript; Body = Between the ATG and stop codon - irrespective of the presence of introns, exons, TSS, or promoters; 3'UTR = Between the stop codon and poly A signa); Mapped gene = reference genome the Illumina Infinium HumanMethylation450 BeadChip manifest file (Illumina, Inc., San Diego, CA).

**3.2.2 CELSPAC: YA cohort.**

The CELSPAC: YA cohort includes saliva samples from young adults. The table below shows the descriptive statistics of the study participants of each sub-cohort, including the exposure variables (Table S3).

**Table S4:** Descriptive statistic of CELSPAC: YA sub-cohorts, including participants data and exposure variable.

| **sub cohort** | **N** | **Age Mean±sd** | **Female %** | **Smoker %** | **Exposure** | **n** | **Miss.** | **mean** | **sd** | **min** | **Q0.25** | **Q0.5** | **Q0.75** | **max** | **IQR** |
| --- | --- | --- | --- | --- | --- | --- | --- | --- | --- | --- | --- | --- | --- | --- | --- |
| HBA | 260 | 29.5 ± 0.6 | 48.5 | 20.8 | PM2.5ann. | 254 | 6 (2.31%) | 15.4 | 1.2 | 11.0 | 14.6 | 15.6 | 16.1 | 20.7 | 1.5 |
|  |  |  |  |  | NO_2_ann. | 254 | 6 (2.31 %) | 19.3 | 5.6 | 8.4 | 14.9 | 19.2 | 23.0 | 34.9 | 8.1 |
|  |  |  |  |  | PM2.5mon. | 254 | 6 (2.31 %) | 14.2 | 5.2 | 7.3 | 9.7 | 12.7 | 17.5 | 31.5 | 7.8 |
|  |  |  |  |  | NO_2_mon. | 254 | 6 (2.31 %) | 18.7 | 6.3 | 4.4 | 13.9 | 18.6 | 23.1 | 35.5 | 9.2 |
|  |  |  |  |  | O_3_ | 254 | 6 (2.31 %) | 92.0 | 1.9 | 83.3 | 91.2 | 91.9 | 93.2 | 96.4 | 2.1 |
|  |  |  |  |  | LAN | 248 | 12 (4.62 %) | 53.1 | 15.2 | 8.0 | 52.0 | 61.0 | 62.0 | 63.0 | 10.0 |
|  |  |  |  |  | MSAVI | 254 | 6 (2.31 %) | 0.6 | 0.1 | 0.4 | 0.6 | 0.6 | 0.7 | 0.8 | 0.1 |
|  |  |  |  |  | Urbanicity | 254 | 7 (2.31 %) | 33.9 | 17.5 | 0.0 | 21.2 | 34.4 | 49.0 | 76.3 | 27.8 |
| STOMA | 46 | 14.6 ± 0.9 | 47.8 | 0 | PM2.5ann. | 46 | 0 | 25.3 | 2.3 | 19.5 | 23.4 | 25.9 | 27.3 | 28.7 | 3.9 |
|  |  |  |  |  | NO_2_ann. | 46 | 0 | 24.1 | 5.7 | 13.5 | 19.8 | 24.0 | 28.0 | 38.8 | 8.2 |
|  |  |  |  |  | PM2.5mon. | 46 | 0 | 27.7 | 13.8 | 10.9 | 18.5 | 22.2 | 33.4 | 64.7 | 14.9 |
|  |  |  |  |  | NO_2_mont. | 46 | 0 | 27.3 | 8.7 | 11.8 | 21.7 | 24.6 | 34.4 | 50.8 | 12.7 |
|  |  |  |  |  | O_3_ | 46 | 0 | 87.8 | 4.3 | 77.4 | 85.4 | 87.8 | 90.6 | 97.5 | 5.1 |
|  |  |  |  |  | LAN | 46 | 0 | 52.9 | 10.3 | 11.0 | 54.0 | 57.0 | 57.0 | 58.0 | 3.0 |
|  |  |  |  |  | MSAVI | 46 | 0 | 0.6 | 0.1 | 0.4 | 0.5 | 0.6 | 0.6 | 0.8 | 0.1 |
|  |  |  |  |  | Urbanicity | 46 | 0 | 33.5 | 16.4 | 4.6 | 22.0 | 33.4 | 46.4 | 61.3 | 24.4 |
| VULDE | 89 | 23.8 ± 0.4 | 47.2 | 33.7 | PM2.5ann. | 89 | 0 | 19.5 | 1.5 | 15.4 | 18.8 | 19.9 | 20.7 | 21.9 | 1.9 |
|  |  |  |  |  | NO_2_ann. | 89 | 0 | 21.5 | 4.3 | 10.9 | 18.6 | 22.0 | 23.9 | 33.1 | 5.3 |
|  |  |  |  |  | PM2.5mon. | 89 | 0 | 14.6 | 4.9 | 10.3 | 11.7 | 12.3 | 17.4 | 30.9 | 5.7 |
|  |  |  |  |  | NO_2_mon. | 89 | 0 | 18.4 | 4.8 | 7.4 | 15.0 | 18.5 | 21.5 | 27.5 | 6.6 |
|  |  |  |  |  | O_3_ | 89 | 0 | 83.1 | 2.4 | 77.5 | 81.5 | 82.4 | 85.1 | 89.1 | 3.6 |
|  |  |  |  |  | LAN | 89 | 0 | 52.0 | 10.7 | 10.0 | 53.0 | 56.0 | 57.0 | 58.0 | 4.0 |
|  |  |  |  |  | MSAVI | 89 | 0 | 0.6 | 0.1 | 0.3 | 0.6 | 0.6 | 0.7 | 0.8 | 0.1 |
|  |  |  |  |  | Urbanicity | 89 | 0 | 30.9 | 15.9 | 1.1 | 19.8 | 31.9 | 41.7 | 61.2 | 21.8 |

PM2.5: particulate matter with aerodynamic diameter < 2.5 μm; NO_2_: nitrogen dioxide; O_3_: ozone; LAN: light at night; MSAVI: modified soil adjusted index. Abbreviations: n: number of observations; Miss.: missing values; sd: standard deviation; min: minimum; Q0.25: 25th percentile; Q0.5: 50th percentile (median); Q0.75: 75th percentile; max: maximum; IQR: interquartile range.

**Table S5:** Genomic inflation factor (ʎ) after bacon correction. Results with ʎ > 1.3 and < 0.8 were considered inflated (highlighted in red) and not included in in further analysis.

| **Sub-cohort** | **PM2.5ann.** | **PM2.5mon.** | **NO_2_ann.** | **NO_2_mont.** | **O_3_** | **LAN** | **MSAVI** | **Urbanicity** |
| --- | --- | --- | --- | --- | --- | --- | --- | --- |
| VULDE | 0.97 | 1.09 | 1.09 | 1.02 | 0.97 | 0.99 | 1.02 | 1.01 |
| STOMA | 1.02 | 1.05 | 1.19 | 1.19 | 1.01 | 1.02 | 1.71 | 1.28 |
| HBA | 1.05 | 1.00 | 1.00 | 2.63 | 0.91 | 1.031 | 0.98 | 1.05 |

The results of the meta-analysis (conducted on cohorts with blood samples) were compared with those obtained in the CELSPAC: YA sub-cohorts to evaluate the consistency of effect direction in saliva samples. The EWAS results from the CELSPAC: YA sub-cohorts were incorporated in the forest plots previously generated for the blood-based cohorts for comparison purposes.

Results from the STOMA analyses for MSAVI and urbanicity, as well as monthly NO_2_ from the HBA sub-cohort, were excluded due to high genomic inflation factor (Table S4). The CpG site Cg17205313 (MSAVI) was not observed in VULDE results, nor was cg06651605 (PM2.5 annual), likely did not pass the quality control criteria. In VULDE, MSAVI was significantly associate with Cg16005697, consistent with SAPALDIA study, and with cg23346227, which was not observed in the other adult cohorts. It is important to consider that the sample size of CELSPAC sub-cohorts is relatively small compared to the other studies, which may influence the statistical power.


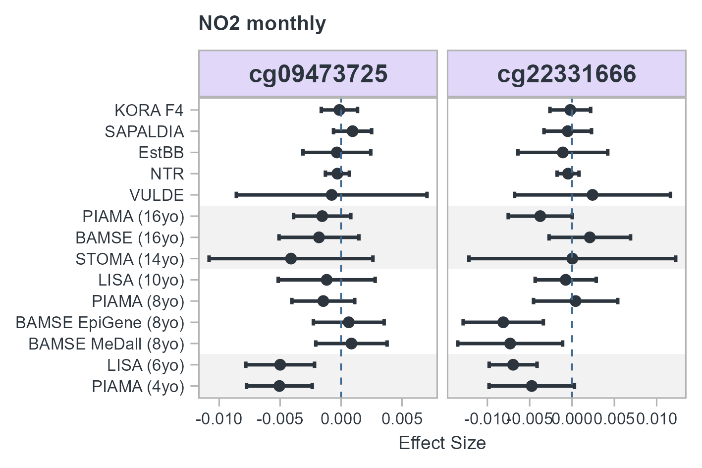

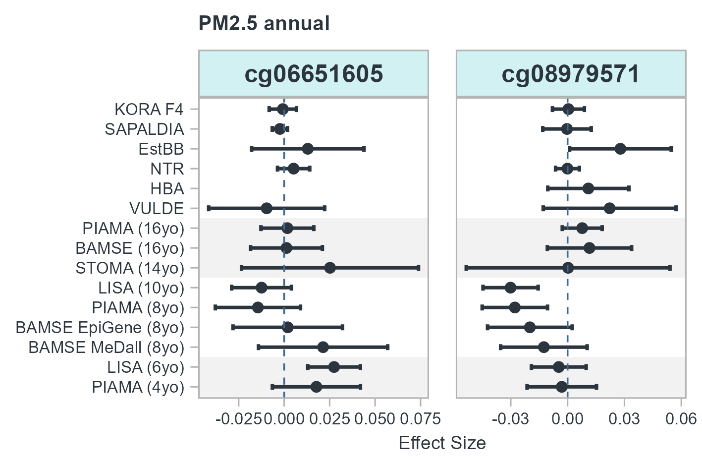

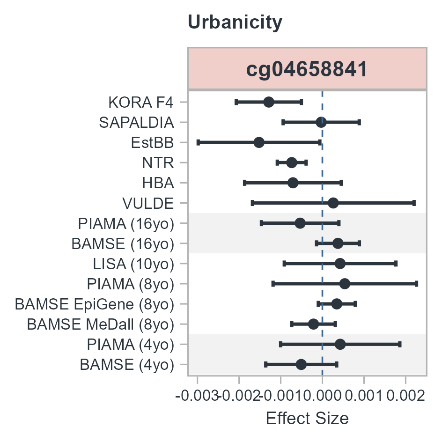

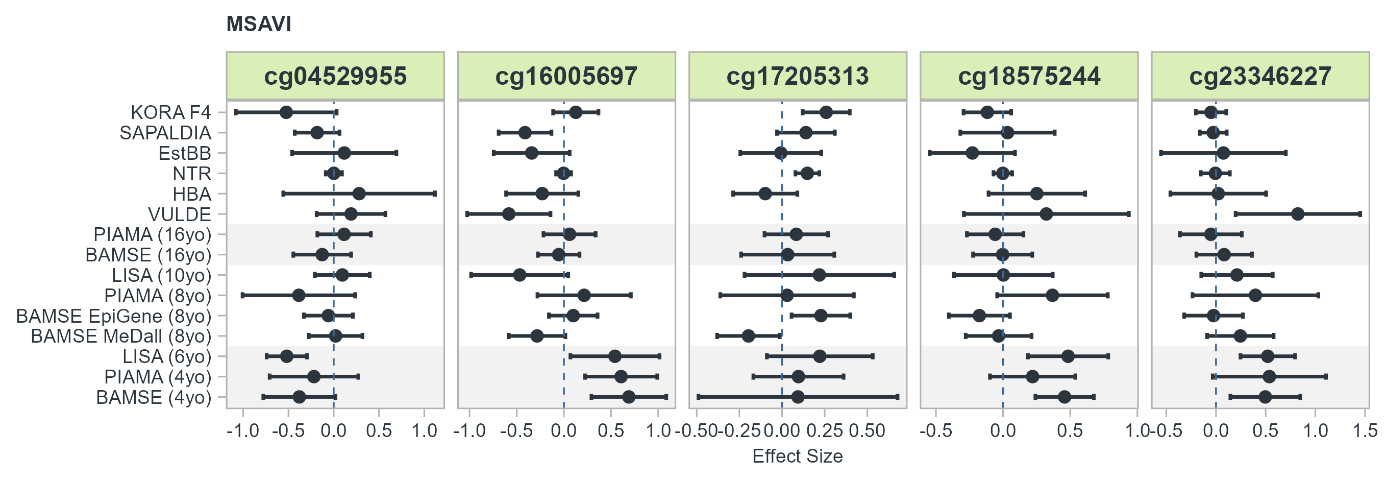


**Figure S2:** Effect estimates (CI) for the significant DMPs identified in the meta-analysis, presented across all cohorts, including CELSPAC results YA sub-cohorts from saliva samples. Results are grouped by exposure and ordered by the cohorts' average age.

**3.2.3** **Summary tables of meta-analysis results meeting the suggestive significance threshold**

**Table S6:** Children 4-6 years: meta-analysis results of the different exposures, meeting the suggestive significance threshold (p-value < 1 × 10⁻⁵). CpGs with I^2^ > 70% are not shown in the table.

| **Exposures** | **MarkerName** | **Effect** | **StdErr** | **pvalue** | **Direction** | **I^2^** | **FDR** | **RefGene** | **chr** | **pos** |
| --- | --- | --- | --- | --- | --- | --- | --- | --- | --- | --- |
| **PM2.5 Annual** | cg06651605 | 0.029 | 0.006 | 2.44E-07 | +++ | 0 | 0.09 |  | 5 | 178209759 |
|  | cg07292773 | -0.040 | 0.009 | 9.01E-06 | --- | 0 | 0.31 |  | 6 | 156718177 |
|  | cg21252280 | 0.027 | 0.006 | 2.86E-06 | +++ | 19.5 | 0.26 | SLC4A11 | 20 | 3209343 |
|  | cg23159704 | 0.039 | 0.009 | 7.36E-06 | +++ | 0 | 0.31 |  | 20 | 62570397 |
|  | cg04611466 | 0.034 | 0.008 | 4.51E-06 | +++ | 22 | 0.31 |  | 5 | 3092821 |
|  | cg10027509 | 0.033 | 0.007 | 2.86E-06 | +++ | 0 | 0.26 | LRSAM1; RPL12 | 9 | 130213144 |
|  | cg06562029 | 0.026 | 0.006 | 8.03E-06 | +++ | 44 | 0.31 | CPEB3 | 10 | 94000570 |
|  | cg00562180 | -0.038 | 0.009 | 8.44E-06 | --- | 0 | 0.31 |  | 16 | 89638519 |
|  | cg25892761 | -0.027 | 0.006 | 8.29E-06 | --- | 0 | 0.31 | RNF213 | 17 | 78318750 |
|  | cg03062881 | -0.029 | 0.006 | 9.43E-07 | --- | 49.9 | 0.17 | PCNT | 21 | 47844012 |
| **NO_2_ Annual** | cg20428720 | 0.008 | 0.002 | 1.51E-06 | -++ | 62.9 | 0.55 | - | 5 | 3093070 |
|  | cg00301033 | 0.005 | 0.001 | 3.15E-06 | +++ | 0 | 0.57 | TTC17 | 11 | 43390036 |
| **NO_2_ Monthly** | cg09473725 | -0.005 | 0.001 | 4.27E-07 | -- | 0 | 0.08 | CD247 | 1 | 167485873 |
|  | cg22331666 | -0.006 | 0.001 | 3.22E-07 | -- | 0 | 0.08 | STX16 | 20 | 57226202 |
|  | cg09178529 | -0.005 | 0.001 | 1.23E-06 | -- | 0 | 0.11 | SPEN; FLJ37453 | 1 | 16174350 |
|  | cg15970890 | 0.005 | 0.001 | 1.14E-06 | ++ | 0 | 0.11 |  | 10 | 17246596 |
|  | cg26381352 | -0.006 | 0.001 | 5.85E-06 | -- | 0 | 0.27 | B3GALT4 | 6 | 33244799 |
|  | cg05511815 | -0.005 | 0.001 | 9.19E-06 | -- | 21 | 0.27 | RANBP3 | 19 | 5978281 |
|  | cg14595786 | -0.006 | 0.001 | 3.98E-06 | -- | 0 | 0.27 | SIGLEC9 | 19 | 51626986 |
|  | cg24348114 | -0.005 | 0.001 | 6.64E-06 | -- | 0 | 0.27 | USF2;LSR | 19 | 35758855 |
| **O3  (warm season)** | cg08302912 | 0.013 | 0.003 | 1.58E-06 | +++ | 1 | 0.44 |  | 6 | 166228349 |
|  | cg02438545 | -0.007 | 0.002 | 2.44E-06 | --- | 6.1 | 0.44 | CDC14B | 9 | 99380444 |
|  | cg15002204 | -0.014 | 0.003 | 5.55E-06 | --- | 55.2 | 0.60 | CENPQ; MUT | 6 | 49429719 |
|  | cg01690062 | -0.012 | 0.003 | 7.78E-06 | --- | 0 | 0.60 |  | 14 | 61125450 |
|  | cg04605932 | -0.012 | 0.003 | 8.31E-06 | --- | 0 | 0.60 | ZNF20 | 19 | 12251213 |
| **LAN** | cg16784566 | 0.003 | 0.001 | 5.60E-06 | -++ | 0 | 0.15 | LRPAP1 | 4 | 3514627 |
|  | cg18123043 | 0.003 | 0.001 | 3.04E-06 | +-+ | 64.7 | 0.15 |  | 4 | 1022462 |
|  | cg00499201 | 0.005 | 0.001 | 4.26E-06 | -++ | 0 | 0.15 |  | 7 | 152600590 |
|  | cg02583740 | 0.004 | 0.001 | 2.13E-06 | +++ | 0 | 0.15 | PRKAR1B | 7 | 612613 |
|  | cg00150989 | 0.003 | 0.001 | 2.45E-06 | +++ | 0 | 0.15 | QRICH2 | 17 | 74303935 |
|  | cg09252214 | 0.003 | 0.001 | 4.60E-06 | +++ | 0 | 0.15 | ZNF574 | 19 | 42584732 |
|  | cg20428720 | 0.004 | 0.001 | 4.93E-06 | -++ | 0 | 0.15 |  | 5 | 3093070 |
|  | cg02409878 | -0.004 | 0.001 | 5.02E-06 | +-- | 17.3 | 0.15 | OSR2 | 8 | 99960498 |
|  | cg05468346 | 0.004 | 0.001 | 3.05E-06 | -++ | 0 | 0.15 | CCDC15 | 11 | 124823826 |
|  | cg22973087 | -0.004 | 0.001 | 5.71E-06 | --- | 0 | 0.15 | LIN7C; BDNFOS | 11 | 27528587 |
|  | cg07021447 | -0.003 | 0.001 | 5.96E-06 | --- | 0 | 0.15 | ACTN1 | 14 | 69418951 |
|  | cg09867343 | -0.004 | 0.001 | 1.21E-06 | --- | 0 | 0.15 | LOC652276 | 16 | 2664939 |
|  | cg15805553 | -0.004 | 0.001 | 2.31E-06 | +-- | 0 | 0.15 | HEATR6 | 17 | 58156359 |
|  | cg00094858 | -0.004 | 0.001 | 4.82E-06 | --- | 62.1 | 0.15 | PPFIA3; C19orf73 | 19 | 49623089 |
|  | cg07444473 | -0.004 | 0.001 | 6.77E-06 | +-- | 49.4 | 0.16 | FLJ32063 | 2 | 200332380 |
|  | cg11097954 | 0.004 | 0.001 | 7.92E-06 | -++ | 27 | 0.18 |  | 14 | 22564251 |
|  | cg17474222 | 0.003 | 0.001 | 9.41E-06 | -++ | 0 | 0.20 | KIAA1543 | 19 | 7680053 |
| **MSAVI** | cg16005697 | 0.620 | 0.121 | 3.34E-07 | +++ | 0 | 0.05 |  | 11 | 24101781 |
|  | cg18575244 | 0.409 | 0.079 | 1.88E-07 | +++ | 0 | 0.05 | UBR7 | 14 | 93693535 |
|  | cg23346227 | 0.515 | 0.103 | 6.11E-07 | +++ | 0 | 0.06 | BAZ2A | 12 | 57024964 |
|  | cg04529955 | -0.449 | 0.092 | 1.07E-06 | --- | 0 | 0.08 | SLC10A7 | 4 | 147442738 |
|  | cg07262244 | -0.495 | 0.104 | 1.84E-06 | --- | 66.8 | 0.11 | LIMS2 | 2 | 128433489 |
|  | cg15401317 | 0.456 | 0.099 | 3.66E-06 | +++ | 0 | 0.19 | NARS2 | 11 | 78180825 |
|  | cg04112181 | 0.532 | 0.117 | 5.18E-06 | +++ | 0 | 0.23 |  | 6 | 168680286 |
|  | cg18634538 | -0.409 | 0.091 | 7.56E-06 | --- | 0 | 0.27 |  | 14 | 77065980 |
|  | cg26832639 | 0.576 | 0.129 | 8.27E-06 | +++ | 0 | 0.27 | KLK5 | 19 | 51457480 |
|  | cg12079381 | -0.461 | 0.103 | 8.06E-06 | --- | 0 | 0.27 | GRIK1 | 21 | 31310920 |
| **Urbanicity** | cg12587766 | 0.001 | 0.000 | 1.79E-06 | ++ | 31.1 | 0.23 | LIFR | 5 | 38556435 |
|  | cg18178010 | 0.001 | 0.000 | 1.45E-06 | ++ | 0 | 0.78 | TNXB | 6 | 32055629 |
|  | cg00366818 | 0.001 | 0.000 | 9.54E-07 | ++ | 0 | 0.79 | ARMC4 | 10 | 28287879 |
|  | cg22595443 | 0.001 | 0.000 | 1.51E-06 | ++ | 0 | 0.61 |  | 10 | 14510114 |
|  | cg26373179 | 0.002 | 0.000 | 7.95E-07 | ++ | 0 | 0.38 | LYPD5 | 19 | 44306615 |
|  | cg07416315 | -0.001 | 0.000 | 2.67E-06 | -+ | 59.7 | 0.12 | FAM19A5 | 22 | 48897200 |
|  | cg10451253 | 0.001 | 0.000 | 5.55E-06 | +- | 68.5 | 0.07 | TMEM132E; C17orf102 | 17 | 32907705 |
|  | cg05098432 | -0.001 | 0.000 | 5.65E-06 | -- | 0 | 0.84 | PLCE1 | 10 | 95848734 |
|  | cg18127003 | 0.002 | 0.000 | 8.44E-06 | ++ | 0 | 0.98 | GATA4 | 8 | 11614472 |
|  | cg10777679 | -0.001 | 0.000 | 7.14E-06 | -- | 21.9 | 0.26 | VRK1 | 14 | 97263710 |
|  | cg19800427 | -0.001 | 0.000 | 9.79E-06 | -- | 0 | 0.38 | ASZ1 | 7 | 117067978 |
|  | cg16538606 | -0.002 | 0.000 | 9.91E-06 | -- | 0 | 0.92 |  | 14 | 64111826 |

FDR* = False Discovery Rate 10% (threshold); StdErr = standard error; Description of the annotation columns: CHR = chromosome number and pos = position of the methylated site on the DNA, provided by Illumina; UCSC_RefGen = reference genome (e.g., hg19 or hg38), used for probe alignment and genomic feature annotation.

**Table S7:** Children 8-10 years: meta-analysis results of the different exposures, meeting the suggestive significance threshold (p-value < 1 × 10⁻⁵). CpGs with I^2^ > 70% are not shown in the table.

| **Exposures** | **MarkerName** | **Effect** | **StdErr** | **pvalue** | **Direction** | **I^2^** | **FDR** | **RefGene** | **CHR** | **pos** |
| --- | --- | --- | --- | --- | --- | --- | --- | --- | --- | --- |
| **PM2.5 Annual** | cg08979571 | -0.025 | 0.005 | 8.33E-08 | ---- | 0 | 0.03 | C2CD4C | 19 | 408233 |
|  | cg09373025 | -0.027 | 0.005 | 6.29E-07 | ---- | 0 | 0.11 | UCKL1 | 20 | 62571664 |
|  | cg13238990 | -0.029 | 0.006 | 4.03E-06 | ---- | 0 | 0.25 | NLGN1 | 3 | 173115252 |
|  | cg04783977 | -0.025 | 0.005 | 3.11E-06 | ---- | 7.5 | 0.25 | UPK1A | 19 | 36157533 |
|  | cg20617669 | -0.023 | 0.005 | 5.78E-06 | ---- | 0 | 0.28 | C1orf86 | 1 | 2138442 |
|  | cg06075233 | -0.027 | 0.006 | 6.24E-06 | ---- | 31.8 | 0.28 | MAP1S | 19 | 17838392 |
|  | cg09865015 | -0.036 | 0.008 | 7.48E-06 | ---- | 0 | 0.30 | LOC100130872;  LOC100130872-SPON2 | 4 | 1202509 |
| **NO_2_ Annual** | - | - | - | - | - | - | - | - | - | - |
| **NO_2_ Monthly** | cg21221883 | 0.004 | 0.001 | 9.78E-06 | ++++ | 0 | 0.81 |  | 1 | 75246543 |
|  | cg05517697 | -0.004 | 0.001 | 2.29E-06 | -+-- | 62.4 | 0.81 | RNF44 | 5 | 175955524 |
| **O_3_ (warm season)** | cg27367235 | -0.009 | 0.002 | 1.16E-06 | ---- | 0 | 0.42 | ZFPM1 | 16 | 88568705 |
|  | cg09102835 | 0.008 | 0.002 | 3.92E-06 | ++++ | 0 | 0.47 | CDK12 | 17 | 37617702 |
|  | cg12043747 | 0.011 | 0.002 | 3.58E-06 | ++++ | 0 | 0.47 | SERPINH1 | 11 | 75272845 |
|  | cg09775260 | -0.006 | 0.001 | 9.18E-06 | ---+ | 7.5 | 0.60 |  | 12 | 115106932 |
| **LAN** | cg05304531 | -0.005 | 0.001 | 8.78E-07 | ---- | 53.7 | 0.26 | DHDDS; HMGN2 | 1 | 26797576 |
|  | cg10625266 | 0.006 | 0.001 | 1.63E-06 | ++++ | 8.7 | 0.26 | SH3GLB1 | 1 | 87169752 |
|  | cg06706029 | -0.004 | 0.001 | 2.14E-06 | -+-- | 0 | 0.26 |  | 16 | 89981752 |
|  | cg25104512 | -0.005 | 0.001 | 6.45E-06 | ---- | 7.4 | 0.32 | ALS2CL | 3 | 46735454 |
|  | cg06296800 | 0.004 | 0.001 | 9.73E-06 | ++++ | 0 | 0.32 | HGFAC | 4 | 3447665 |
|  | cg25970230 | -0.004 | 0.001 | 7.10E-06 | ++-- | 11.5 | 0.32 | POU4F2 | 4 | 147558544 |
|  | cg13658186 | -0.005 | 0.001 | 7.57E-06 | -+-- | 48.5 | 0.32 |  | 14 | 105116585 |
|  | cg21811853 | -0.003 | 0.001 | 7.34E-06 | -+-- | 0 | 0.32 |  | 1 | 25224078 |
|  | cg00242950 | 0.005 | 0.001 | 7.84E-06 | +-++ | 12.8 | 0.32 |  | 3 | 112899162 |
|  | cg27618305 | 0.004 | 0.001 | 8.69E-06 | +-++ | 48.5 | 0.32 | PKD1L1 | 7 | 47867025 |
|  | cg01157143 | -0.004 | 0.001 | 8.91E-06 | +--- | 0 | 0.32 | NAV2 | 11 | 19478542 |
| **MSAVI** | cg10453230 | 0.316 | 0.070 | 6.34E-06 | ++-+ | 23.6 | 0.51 |  | 5 | 174148198 |
|  | cg19885914 | -0.320 | 0.072 | 9.62E-06 | ---- | 0 | 0.51 | TNXB | 6 | 32036530 |
|  | cg08774231 | -0.298 | 0.063 | 2.20E-06 | ---- | 0 | 0.51 | PPP2R2B | 5 | 146258181 |
|  | cg06536578 | 0.254 | 0.057 | 9.55E-06 | ++++ | 0 | 0.51 | JPH4 | 14 | 24048555 |
|  | cg12055302 | -0.299 | 0.066 | 5.59E-06 | ---- | 0 | 0.51 | TTC7B | 14 | 91224978 |
| **Urbanicity** | cg19548763 | 0.0009 | 0.0002 | 1.31E-06 | ++++ | 0 | 0.15 | C18orf | 18 | 13638936 |
|  | cg05989460 | 0.0007 | 0.0001 | 1.64E-06 | ++-+ | 0 | 0.15 | TLE4 | 9 | 82187938 |
|  | cg24031606 | 0.0008 | 0.0002 | 1.82E-06 | ++-+ | 7.8 | 0.15 | CDC25A | 3 | 48229318 |
|  | cg09775260 | 0.0007 | 0.0001 | 2.33E-06 | ++-+ | 42.4 | 0.15 |  | 12 | 115106932 |
|  | cg25233308 | -0.001 | 0.0002 | 2.68E-06 | ---- | 0 | 0.15 | NPAS4 | 11 | 66192121 |
|  | cg11563860 | 0.0008 | 0.0002 | 2.76E-06 | ++-+ | 0 | 0.15 | CNTN4 | 3 | 2141937 |
|  | cg09375034 | 0.0009 | 0.0002 | 2.82E-06 | ++++ | 28.9 | 0.15 | TUBAL3 | 10 | 5437414 |
|  | cg08774231 | 0.0007 | 0.0002 | 3.71E-06 | +++- | 0 | 0.17 | PPP2R2B | 5 | 146258181 |
|  | cg03408433 | 0.0008 | 0.0002 | 5.57E-06 | ++-+ | 53.7 | 0.22 | CMKLR1 | 12 | 108733370 |
|  | cg20908789 | 0.0007 | 0.0002 | 9.71E-06 | ++-+ | 0 | 0.31 |  | 8 | 11551964 |

FDR* = False Discovery Rate 10% (threshold); StdErr = standard error; Description of the annotation columns: CHR = chromosome number and pos = position of the methylated site on the DNA, provided by Illumina; UCSC_RefGen = reference genome (e.g., hg19 or hg38), used for probe alignment and genomic feature annotation.

**Table S8:** Adolescents: meta-analysis results of the different exposures, meeting the suggestive significance threshold (p-value < 1 × 10⁻⁵). CpGs with I^2^ > 70% are not shown in the table.

| **Exposures** | **MarkerName** | **Effect** | **StdErr** | **pvalue** | **Direction** | **I^2^** | **FDR** | **RefGene** | **CHR** | **pos** |
| --- | --- | --- | --- | --- | --- | --- | --- | --- | --- | --- |
| **PM2.5 Annual** | cg07159286 | 0.028 | 0.006 | 1.13E-06 | ++ | 0 | 0.49 | ZNF268 | 12 | 133779169 |
|  | cg16295162 | -0.023 | 0.005 | 2.43E-06 | -- | 0 | 0.50 | PARD6G | 18 | 78005487 |
|  | cg25116237 | 0.022 | 0.005 | 4.62E-06 | ++ | 7.1 | 0.50 | NOTUM | 17 | 79913431 |
|  | cg00458295 | 0.027 | 0.006 | 7.67E-06 | ++ | 0 | 0.50 | ACOXL | 2 | 111593263 |
|  | cg24517252 | -0.024 | 0.005 | 7.86E-06 | -- | 55.1 | 0.50 | PGBD2 | 1 | 249200781 |
|  | cg13384150 | 0.022 | 0.005 | 9.05E-06 | ++ | 0 | 0.50 | NBPF1 | 1 | 16939173 |
|  | cg03028337 | 0.022 | 0.005 | 9.48E-06 | ++ | 0 | 0.50 | CCDC85A | 2 | 56410087 |
|  | cg16546309 | -0.026 | 0.006 | 9.66E-06 | -- | 17 | 0.50 |  | 1 | 244014035 |
| **PM2.5 Monthly** | cg24927135 | -0.011 | 0.002 | 3.78E-06 | -- | 0 | 0.88 | RPS27L | 15 | 63449340 |
|  | cg08468599 | 0.010 | 0.002 | 5.78E-06 | ++ | 0 | 0.88 | SKI | 1 | 2182126 |
|  | cg05046597 | -0.010 | 0.002 | 8.87E-06 | -- | 0 | 0.88 | ZNF398; ZNF425 | 7 | 148823741 |
| **NO_2_ Annual** | cg10091102 | 0.006 | 0.001 | 8.16E-06 | ++ | 0 | 0.70 |  | 12 | 14134922 |
| **NO_2_ Monthly** | cg04154502 | 0.006 | 0.001 | 3.31E-06 | ++ | 0 | 0.82 | ZFR2 | 19 | 3831293 |
|  | cg19889307 | -0.005 | 0.001 | 7.59E-06 | -- | 0 | 0.82 | ADK; AP3M1 | 10 | 75911429 |
|  | cg01354455 | 0.004 | 0.001 | 7.64E-06 | ++ | 62.4 | 0.82 |  | 10 | 3870178 |
|  | cg07878171 | -0.005 | 0.001 | 7.90E-06 | -- | 36.2 | 0.82 | TRAPPC9 | 8 | 141109731 |
| **O3  (warm season)** | cg01668514 | 0.018 | 0.004 | 1.09E-06 | ++ | 0 | 0.47 | GFRA3 | 5 | 137592965 |
|  | cg14452650 | -0.016 | 0.004 | 5.02E-06 | -- | 69 | 0.47 | EML4; EML4 | 2 | 42396274 |
|  | cg16475721 | 0.015 | 0.003 | 7.56E-06 | ++ | 0 | 0.47 |  | 7 | 31232678 |
|  | cg21136499 | 0.017 | 0.004 | 7.66E-06 | ++ | 0 | 0.47 | LFNG | 7 | 2551978 |
|  | cg16864198 | 0.014 | 0.003 | 8.06E-06 | ++ | 0 | 0.47 |  | 12 | 26340863 |
|  | cg15055773 | -0.012 | 0.003 | 8.19E-06 | -- | 27 | 0.47 |  | 7 | 107647750 |
| **LAN** | cg12610087 | -0.002 | 0.001 | 2.23E-06 | -- | 45.7 | 0.48 | LOC285954 | 7 | 41745737 |
|  | cg14012925 | 0.003 | 0.001 | 3.93E-06 | -+ | 18.7 | 0.57 | CUX1 | 7 | 101556907 |
|  | cg25890092 | 0.002 | 0.001 | 7.97E-06 | -+ | 52.6 | 0.67 | CD7 | 17 | 80273322 |
|  | cg14602530 | 0.002 | 0.001 | 8.33E-06 | ++ | 45.3 | 0.67 |  | 4 | 4859772 |
| **MSAVI** | cg18988889 | 0.289 | 0.062 | 2.72E-06 | ++ | 62.4 | 0.52 |  | 7 | 2500004 |
|  | cg10762935 | -0.371 | 0.080 | 3.59E-06 | -- | 65.1 | 0.52 |  | 11 | 63441346 |
|  | cg11699515 | 0.297 | 0.067 | 8.57E-06 | ++ | 0 | 0.62 | ECHS1 | 10 | 135176625 |
| **Urbanicity** | cg17359076 | 0.001 | 0.000 | 1.78E-06 | ++ | 0 | 0.58 | TUBA1C | 12 | 49658802 |
|  | cg21500064 | 0.001 | 0.000 | 5.33E-06 | ++ | 0 | 0.58 | SULF2 | 20 | 46415859 |
|  | cg17691543 | -0.001 | 0.000 | 6.59E-06 | -- | 34.5 | 0.58 | GMPPB | 3 | 49759312 |
|  | cg01947224 | -0.001 | 0.000 | 6.65E-06 | -- | 0 | 0.58 | ZNF416 | 19 | 58089885 |

FDR* = False Discovery Rate 10% (threshold); StdErr = standard error; Description of the annotation columns: CHR = chromosome number and pos = position of the methylated site on the DNA, provided by Illumina; UCSC_RefGen = reference genome (e.g., hg19 or hg38), used for probe alignment and genomic feature annotation.

**Table S9:** Adults: meta-analysis results of the different exposures, meeting the suggestive significance threshold (p-value < 1 × 10⁻⁵). CpGs with I^2^ > 70% are not shown in the table.

| **Exposures** | **MarkerName** | **Effect** | **StdErr** | **pvalue** | **Direction** | **I^2^** | **FDR** | **RefGene** | **CHR** | **pos** |
| --- | --- | --- | --- | --- | --- | --- | --- | --- | --- | --- |
| **PM2.5 Annual** | cg04175049 | 0.009 | 0.002 | 3.71E-06 | +-++ | 0 | 0.69 | SBF1 | 22 | 50894039 |
|  | cg17085748 | -0.010 | 0.002 | 9.67E-06 | ---- | 12.2 | 0.92 | MXD1 | 2 | 70142105 |
| **PM2.5 Monthly** | cg20711803 | -0.005 | 0.001 | 2.67E-07 | --- | 0 | 0.10 | MXI1 | 10 | 111970722 |
|  | cg24658930 | 0.004 | 0.001 | 1.46E-06 | -++ | 0 | 0.22 | RHOBTB3 | 5 | 95066845 |
|  | cg04564000 | -0.007 | 0.002 | 1.76E-06 | --- | 0 | 0.22 | ZBTB20 | 3 | 114343068 |
|  | cg10112003 | -0.005 | 0.001 | 2.51E-06 | -+- | 61.1 | 0.24 | ACTL6A | 3 | 179280652 |
|  | cg06094111 | -0.004 | 0.001 | 5.78E-06 | --- | 37.4 | 0.44 | CNPY2 | 12 | 56709459 |
|  | cg14431072 | -0.008 | 0.002 | 8.20E-06 | --- | 0 | 0.52 | TNXB | 6 | 32027666 |
| **NO_2_ Annual** | cg17205313 | -0.002 | 0.000 | 5.67E-07 | -+-- | 2.3 | 0.18 | TTC39C | 18 | 21572748 |
|  | cg11840035 | -0.003 | 0.001 | 9.73E-07 | ---- | 0 | 0.18 |  | 1 | 113685087 |
|  | cg07707498 | -0.003 | 0.001 | 2.49E-06 | ---- | 0 | 0.26 | C16orf87 | 16 | 46865555 |
|  | cg04051365 | 0.002 | 0.000 | 2.73E-06 | +++- | 52 | 0.26 |  | 3 | 193586394 |
|  | cg11201177 | 0.003 | 0.001 | 4.44E-06 | ++++ | 0 | 0.33 |  | 11 | 2961805 |
|  | cg04658841 | -0.002 | 0.001 | 8.39E-06 | ---+ | 17.1 | 0.46 |  | 16 | 85478651 |
|  | cg02291533 | -0.003 | 0.001 | 9.18E-06 | ---- | 53.1 | 0.46 | LRFN3 | 19 | 36427210 |
| **NO_2_ Monthly** | cg10105699 | -0.002 | 0.000 | 7.60E-06 | ---- | 0 | 1.00 |  | 2 | 37869895 |
| **O3  (warm Season)** | cg02998028 | -0.004 | 0.001 | 1.03E-06 | ---- | 0 | 0.39 | ACMSD | 2 | 135621153 |
| **LAN** | cg27596275 | 0.001 | 0.000 | 3.45E-06 | ++++ | 29 | 0.81 | TMEM218 | 11 | 124971707 |
| **MSAVI** | cg17205313 | 0.157 | 0.029 | 5.53E-08 | +-++ | 25.7 | 0.02 | TTC39C | 18 | 21572748 |
|  | cg11149163 | -0.175 | 0.038 | 3.58E-06 | ---- | 0 | 0.51 |  | 5 | 2387826 |
|  | cg14464245 | -0.100 | 0.022 | 4.53E-06 | ---- | 0 | 0.51 | AGPAT2 | 9 | 139580482 |
|  | cg00251358 | 0.164 | 0.036 | 5.45E-06 | ++++ | 25.2 | 0.51 |  | 5 | 81931501 |
| **Urbanicity** | cg04658841 | -0.001 | 0.000 | 2.37E-07 | ---- | 43 | 0.09 |  | 16 | 85478651 |
|  | cg06791867 | 0.001 | 0.000 | 5.91E-07 | ++++ | 0 | 0.11 | TSPAN18 | 11 | 44880944 |
|  | cg16603012 | 0.001 | 0.000 | 2.25E-06 | ++++ | 26.5 | 0.24 | APRT | 16 | 88879593 |
|  | cg26132647 | 0.000 | 0.000 | 4.20E-06 | +-++ | 0 | 0.24 |  | 3 | 50298315 |
|  | cg17138684 | 0.001 | 0.000 | 4.26E-06 | +++- | 20.8 | 0.24 | TSPAN3 | 15 | 77363627 |
|  | cg18897025 | 0.001 | 0.000 | 4.85E-06 | ++++ | 0 | 0.24 | TSLP | 5 | 110413671 |
|  | cg09457245 | 0.001 | 0.000 | 5.26E-06 | ++++ | 0 | 0.24 | ZNF385A | 12 | 54778750 |
|  | cg12471156 | 0.001 | 0.000 | 5.37E-06 | ++++ | 0 | 0.24 | RBM16 | 6 | 155054259 |
|  | cg14464245 | 0.000 | 0.000 | 6.14E-06 | +-++ | 0 | 0.24 | AGPAT2 | 9 | 139580482 |
|  | cg20686403 | 0.000 | 0.000 | 6.33E-06 | ++++ | 2.6 | 0.24 |  | 22 | 38438206 |
|  | cg07786657 | -0.001 | 0.000 | 7.84E-06 | ---- | 33.6 | 0.27 | CD247 | 1 | 167487633 |

FDR* = False Discovery Rate 10% (threshold); StdErr = standard error; Description of the annotation columns: CHR = chromosome number and pos = position of the methylated site on the DNA, provided by Illumina; UCSC_RefGen = reference genome (e.g., hg19 or hg38), used for probe alignment and genomic feature annotation.

**Table S10:** List of excluded CpG sites that initially met the suggestive significance threshold but were removed due to high heterogeneity (I² > 70%).

|  | **Exposure** | **MarkerNa.** | **Effect** | **StdErr** | **pvalue** | **FDR** | **Dir.** | **I^2^** | **HetPVal** | **RefGene** |
| --- | --- | --- | --- | --- | --- | --- | --- | --- | --- | --- |
| Adults | PM2.5ann. | cg03530740 | 0.009 | 0.002 | 3.07E-06 | 6.94E-01 | +-+- | 80.9 | 0.001 | TCF3 |
| Adolescents | NO_2_mon. | cg07900968 | -0.005 | 0.001 | 9.77E-06 | 8.17E-01 | -- | 73.1 | 0.054 | ZFP82 |
|  | O_3_ | cg07359183 | 0.003 | 0.001 | 3.09E-07 | 1.34E-01 | ++ | 81.5 | 0.020 | PLXNA2 |
|  | MSAVI | cg11986861 | -0.325 | 0.057 | 1.04E-08 | 4.52E-03 | -- | 88.5 | 0.003 | ZNRF3 |
|  | Urbanicity | cg13843611 | 0.001 | 0.000 | 4.73E-06 | 5.77E-01 | ++ | 71.7 | 0.060 | RNF186 |
| Children 8-10 | PM2.5ann. | cg09013975 | -0.023 | 0.005 | 2.21E-06 | 2.52E-01 | +--- | 75.8 | 0.006 | ATP2A3 |
|  | PM2.5ann. | cg02664787 | 0.028 | 0.006 | 4.19E-06 | 2.52E-01 | -+++ | 80.7 | 0.001 | OPCML |
|  | NO2mon. | cg05517697 | -0.004 | 0.001 | 2.29E-06 | 8.07E-01 | -+-- | 62.4 | 0.047 | RNF44 |
|  | MSAVI | cg18223579 | 0.302 | 0.066 | 4.24E-06 | 5.13E-01 | +++- | 78.7 | 0.003 | ESRRG |
| Childre 4-6 | NO_2_mon. | cg21281951 | -0.006 | 0.001 | 6.31E-06 | 2.75E-01 | -- | 79.6 | 0.027 | HIST1H3G;HIST1H2BI |
|  | MSAVI | cg23882164 | 0.425 | 0.084 | 4.08E-07 | 4.91E-02 | +-+ | 87.1 | 0.000 | - |
|  | Urbanicity | cg04740258 | -0.001 | 0.000 | 6.03E-06 | 2.92E-01 | -+ | 75.6 | 0.043 | - |
|  | Urbanicity | cg14938313 | 0.001 | 0.000 | 7.97E-06 | 2.96E-01 | +- | 83.2 | 0.015 | ANO1 |

FDR* = False Discovery Rate 10% (threshold); StdErr = standard error; HetPVal = Cochran’s Q test results; MarkerNa. = MarkerName; Dir. = Direction; UCSC_RefGen = reference genome (e.g., hg19 or hg38), used for probe alignment and genomic feature annotation.

**3.2.4 Intersection analysis by Age Group for the different exposures**


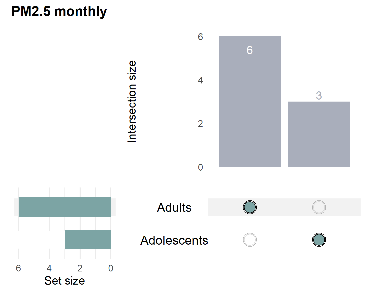

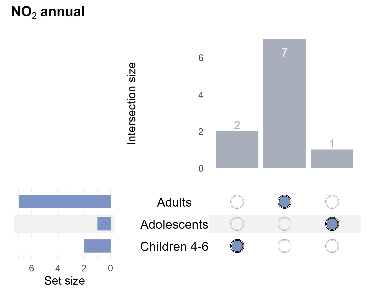

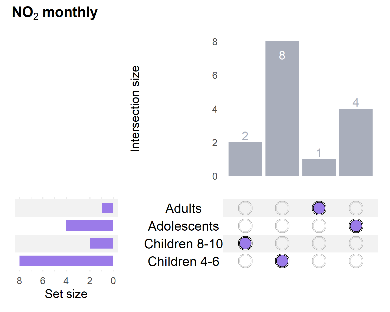

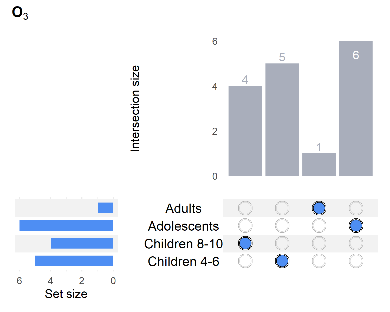

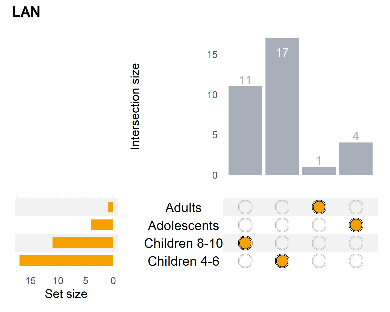

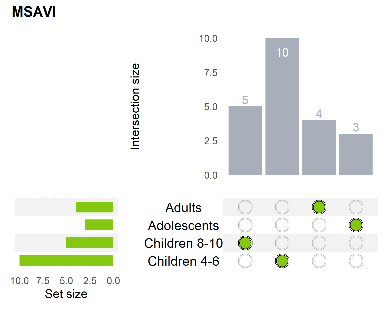

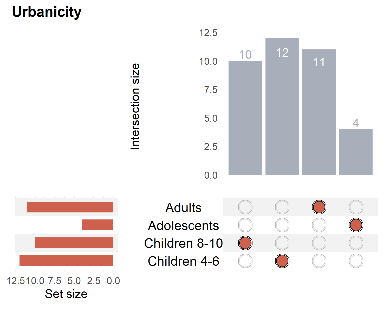

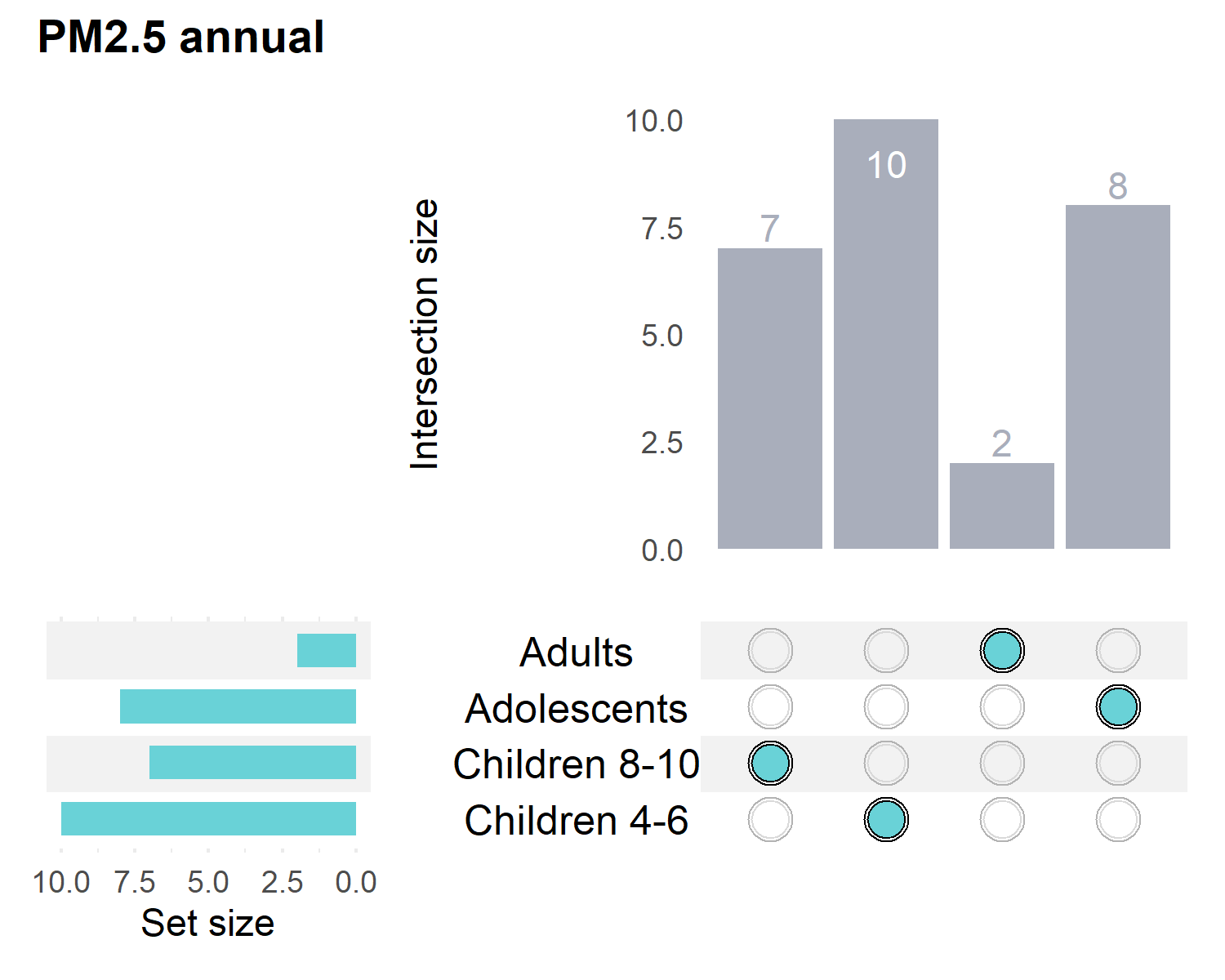


**Figure S3:** Intersection analysis of CpG sites meeting the suggestive significance threshold (p < 1×10⁻⁵) for each environmental exposure, stratified by age group. Bars are color-coded by exposure. The UpSet plot displays intersections and unique sets of significant CpG sites across age groups; no intersections between age groups were observed.

**3.2.5 Integrative Expression Quantitative Trait Methylation (eQTM) Analysis**

**Table S11:** Summary results table of eQTM integration analysis conducted on CpGs that have met a suggestive significance threshold across the different Age-group for the different exposures.

| **Age group** | **Exposure** | **MarkerName** | **C/T** | **HGNC** | **Beta (SE)** | **PValue** | **FDR** |
| --- | --- | --- | --- | --- | --- | --- | --- |
| Adults | Urbanicity | cg07786657 | cis | CD247 | 0.0859561 (0.0391686);0.0345469 (0.0401694);0.1528324 (0.0389124);0.0182159 (0.0741126) | 8.47E-05 | 0.017643 |
| Adolescent | PM2.5 ann. | cg13384150 | cis | MST1L | -0.0221803 (0.0393044);-0.0515519 (0.0401399);-0.1536881 (0.0389072);-0.2729267 (0.0713108) | 1.66e-05 | 0.004349 |
| Adolescent | Urbanicity | cg01947224 | cis | ZNF154 | -0.2524656 (0.0380405);-0.1597784 (0.039677);-0.2335111 (0.0382864);-0.333878 (0.0698714) | 7.64E-26 | 0 |
| Adolescent | Urbanicity | cg01947224 | cis | ZNF304 | -0.1953915 (0.0385563);-0.1412719 (0.0397903);-0.235128 (0.0382711);-0.3211063 (0.0701995) | 7.17E-21 | 0 |
| Adolescent | Urbanicity | cg01947224 | cis | ZNF671 | -0.1723636 (0.0387257);-0.1706411 (0.0396039);-0.2089426 (0.0385059);-0.2671311 (0.0714312) | 9.59E-19 | 0 |
| Adolescent | Urbanicity | cg01947224 | cis | ZIK1 | -0.1023545 (0.0391076);-0.1510946 (0.0397319);-0.2292674 (0.0383261);-0.227916 (0.072174) | 1.48E-14 | 0 |
| Adolescent | Urbanicity | cg01947224 | cis | ZNF211 | -0.0980995 (0.0391244);-0.1163916 (0.0399202);-0.244293 (0.038182);-0.2132611 (0.0724197) | 2.64E-13 | 0 |
| Adolescent | Urbanicity | cg01947224 | cis | ZNF772 | -0.1595336 (0.0388105);-0.1137508 (0.0399325);-0.1630308 (0.0388482);-0.1911863 (0.0727576) | 5.11E-12 | 0 |
| Adolescent | Urbanicity | cg01947224 | cis | ZNF547 | -0.156552 (0.0388293);-0.1188933 (0.0399083);-0.1480114 (0.0389413);-0.2275967 (0.0721796) | 6.64E-12 | 0 |
| Adolescent | Urbanicity | cg01947224 | cis | ZNF134 | -0.1300202 (0.0389803);-0.1013457 (0.0399864);-0.1618789 (0.0388556);-0.2535829 (0.0717021) | 6.10E-11 | 0 |
| Adolescent | Urbanicity | cg01947224 | cis | ZNF551 | -0.1098094 (0.0390763);-0.1151771 (0.0399259);-0.135735 (0.0390106);-0.1879498 (0.0728039) | 6.61E-09 | 0 |
| Adolescent | Urbanicity | cg01947224 | cis | ZNF419 | -0.1417182 (0.0389173);-0.1004628 (0.03999);-0.1398309 (0.0389881);-0.1052346 (0.0737133) | 7.72E-09 | 0 |
| Adolescent | Urbanicity | cg01947224 | cis | ZNF549 | -0.0936586 (0.0391412);-0.0601564 (0.0401206);-0.1587864 (0.0388754);-0.2555385 (0.0716639) | 5.46E-08 | 9.46E-06 |
| Adolescent | Urbanicity | cg01947224 | cis | ZNF530 | -0.11081 (0.0390719);-0.0576954 (0.0401264);-0.1666922 (0.0388241);-0.1481614 (0.0733068) | 1.03E-07 | 3.62E-05 |
| Adolescent | Urbanicity | cg01947224 | cis | ZNF773 | -0.0994287 (0.0391192);-0.0511997 (0.0401407);-0.1539845 (0.0389053);-0.1021432 (0.0737372) | 2.83E-06 | 0.000914 |
| Adolescent | Urbanicity | cg01947224 | cis | ZNF416 | -0.0938429 (0.0391406);-0.0388148 (0.0401631);-0.1154913 (0.0391115);-0.1372329 (0.0734236) | 5.50E-05 | 0.012169 |
| Child. 8-10 | PM2.5 ann. | cg09373025 | cis | UCKL1 | -0.0748927 (0.0392036);-0.1422984 (0.0397844);-0.068538 (0.0392824);0.0274275 (0.074097) | 0.000115 | 0.022627 |
| Child. 4-6 | PM2.5 ann. | cg23159704 | cis | UCKL1 | -0.1540878 (0.0388445);-0.1642008 (0.0396478);-0.1264392 (0.039059);-0.2910604 (0.0709157) | 1.47E-13 | 0 |
| Child. 4-6 | PM2.5 ann. | cg03062881 | cis | PCNT | -0.1948383 (0.0385606);-0.1330798 (0.0398359);-0.0794067 (0.0392506);-0.2258528 (0.0722097) | 3.68E-11 | 0 |
| Child. 4-6 | PM2.5 ann. | cg03062881 | cis | PRMT2 | -0.2001849 (0.0385183);0.0171789 (0.0401875);-0.1219788 (0.0390809);-0.0027888 (0.0741246) | 1.29E-05 | 0.003549 |

C/T = Cis / Trans; HGNC = official gene symbol according to the HUGO Gene Nomenclature Committee (HGNC); Beta (SE) = Beta (effect size) and SE calculated in the individual dataset; FDR* = False Discovery Rate 10% (threshold); StdErr = standard error. Note: FDR values equal to 0 reflect extremely small adjusted p-values below R’s numerical precision and should be interpreted as FDR < 1×10⁻¹⁶.

- 1. **DMRs Results**


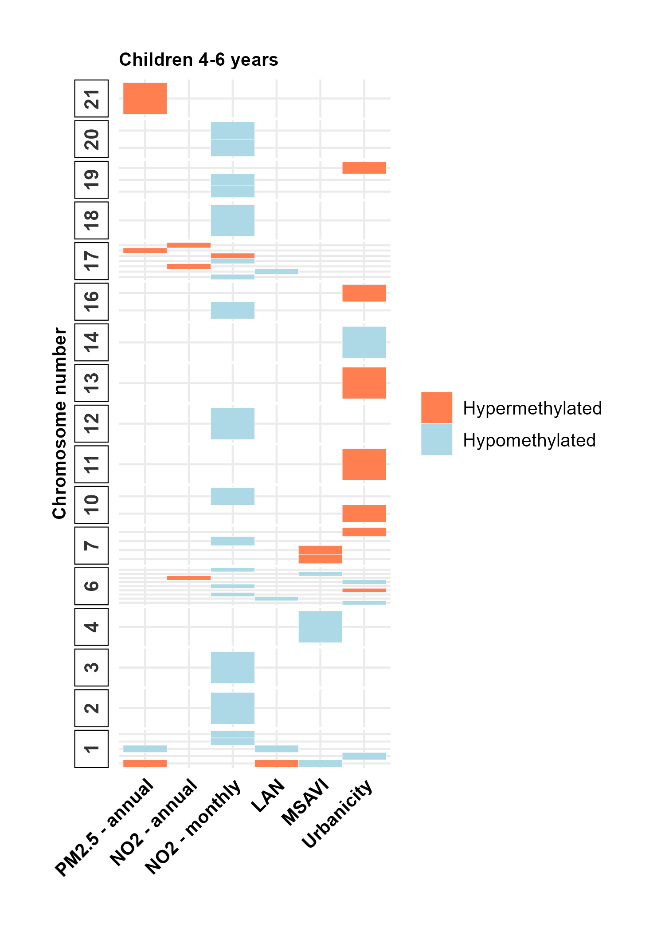

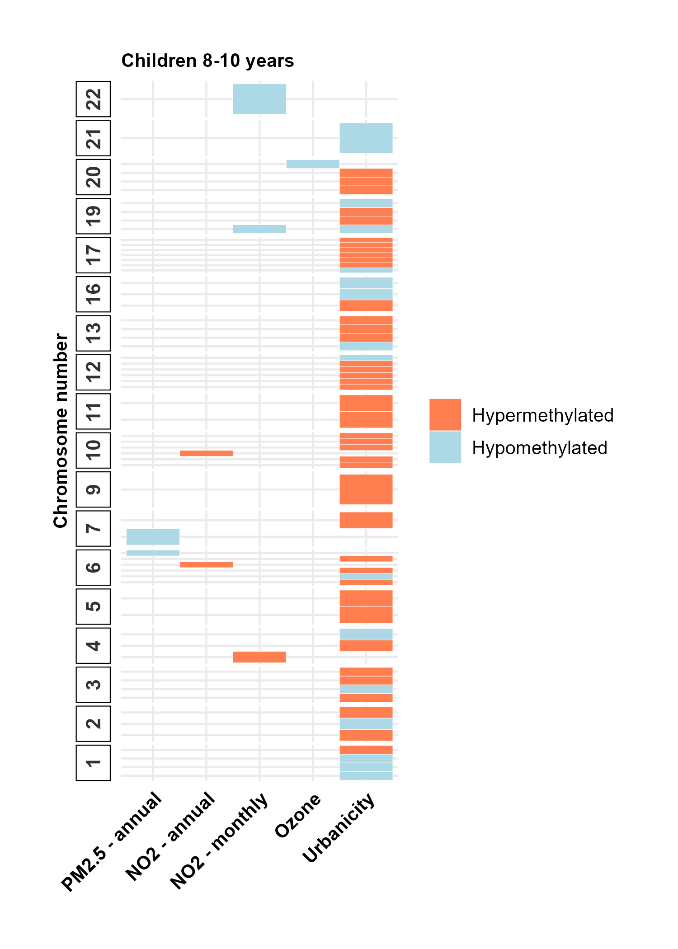

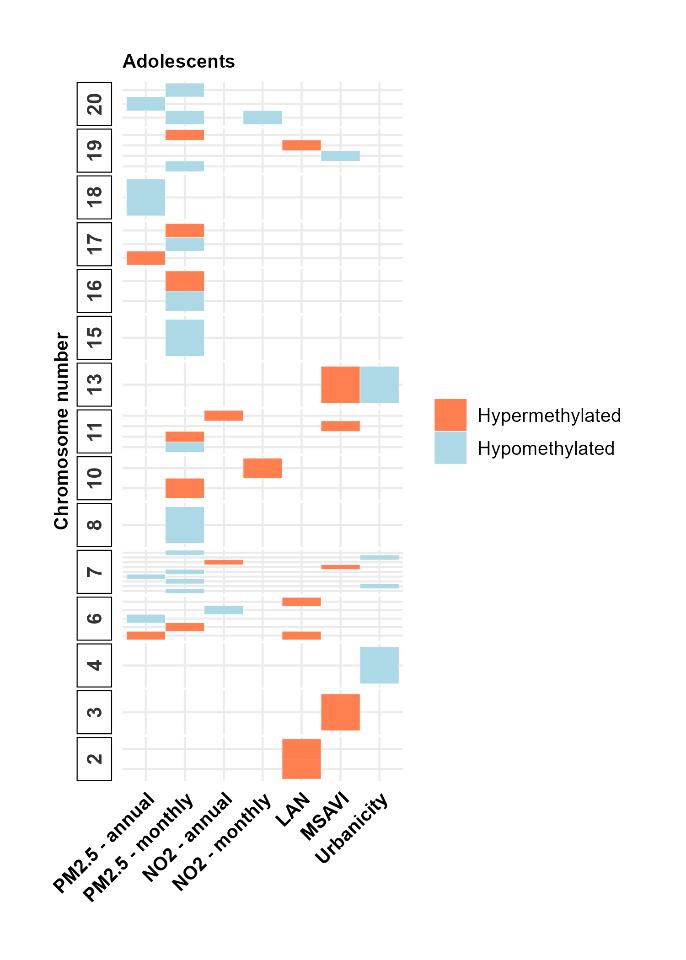

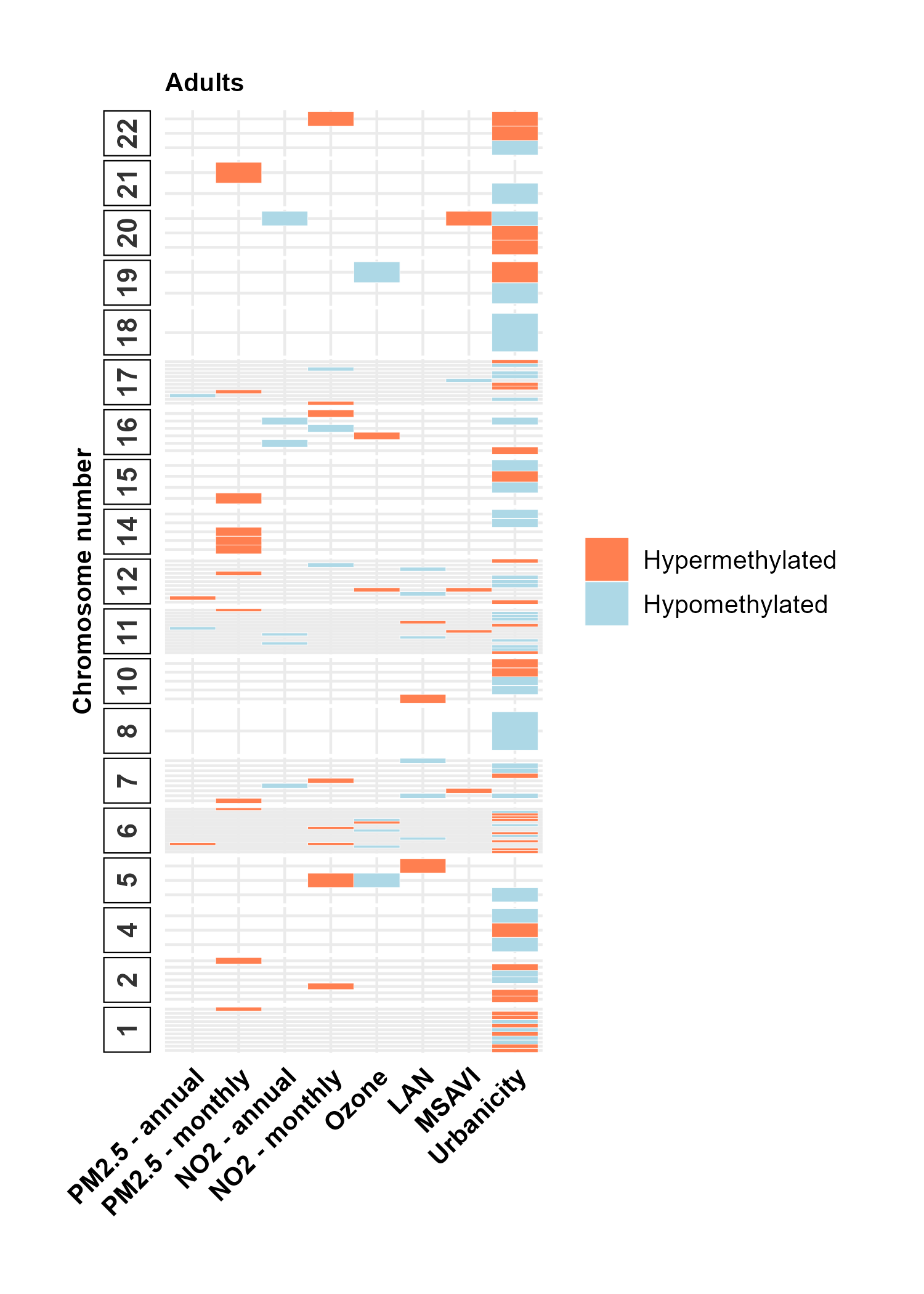


**Figure S4:** Heatmaps grouped by life stage, displaying regions with a positive mean differential (indicating higher methylation levels) in red and those with a negative mean differential (indicating lower methylation levels) in blue. Results are organized by chromosome, and all included DMRs meet the criterion of a minimum smoothed FDR of < 0.05.

- - 1. **Comparison with ipDMR results**

The ipDMR analysis identified fewer DMRs per each age group, with a lower number of CpGs included in the DMRs compared to the DMRcate results. Despite these differences, consistent findings were observed for children 4-6 years and adults. In children aged 4-6 years, annual PM2.5 exposure was associated to a DMR located on chromosome 17 and mapped to the *SLC16A3* gene. Similarly, DMRcate identified associations for both annual PM2.5 and NO_2_ with the same DMR. Additionally, both methods identified a DMR mapped to *GALNT2* in association with monthly NO_2_ exposure. However, while with ipDMR association was observed only with monthly NO_2_, with DMRcate we observed association also with annual PM2.5 and LAN (on the same position). Lastly, exposure to monthly NO_2_ was consistently associated to a DMR on chromosome 1 mapped to *RUNX3*, in both methods. For adults, overlapping results were also observed between the two approaches. LAN was associated to a DMR on chromosome 6 mapped to *HLA-E* in the ipDMR analysis, whereas DMRcate linked it to urbanicity. Additionally, urbanicity was consistently associated to DMRs mapped to *STOX2,* on chromosome 4, and to *SSH1* on chromosome 12, across both methods. Further information is available in *S2_Tables_ipDMR*.

**3.4 Pathway enrichment analysis**

**Figure S5:** Results of Gene Ontology (Biological Process) enrichment analysis performed with Enrichr (2). Analyses were based on genes mapped to CpGs included in significant DMRs, which were linked to transcripts via blood cis-eQTMs from the BIOS QTL browser prior to enrichment analysis. Only significant pathways (FDR < 0.05, including at least two genes per pathway) are presented and stratified by age group. Dot colors indicate the corresponding exposures, while dot size reflects the number of overlapping genes.


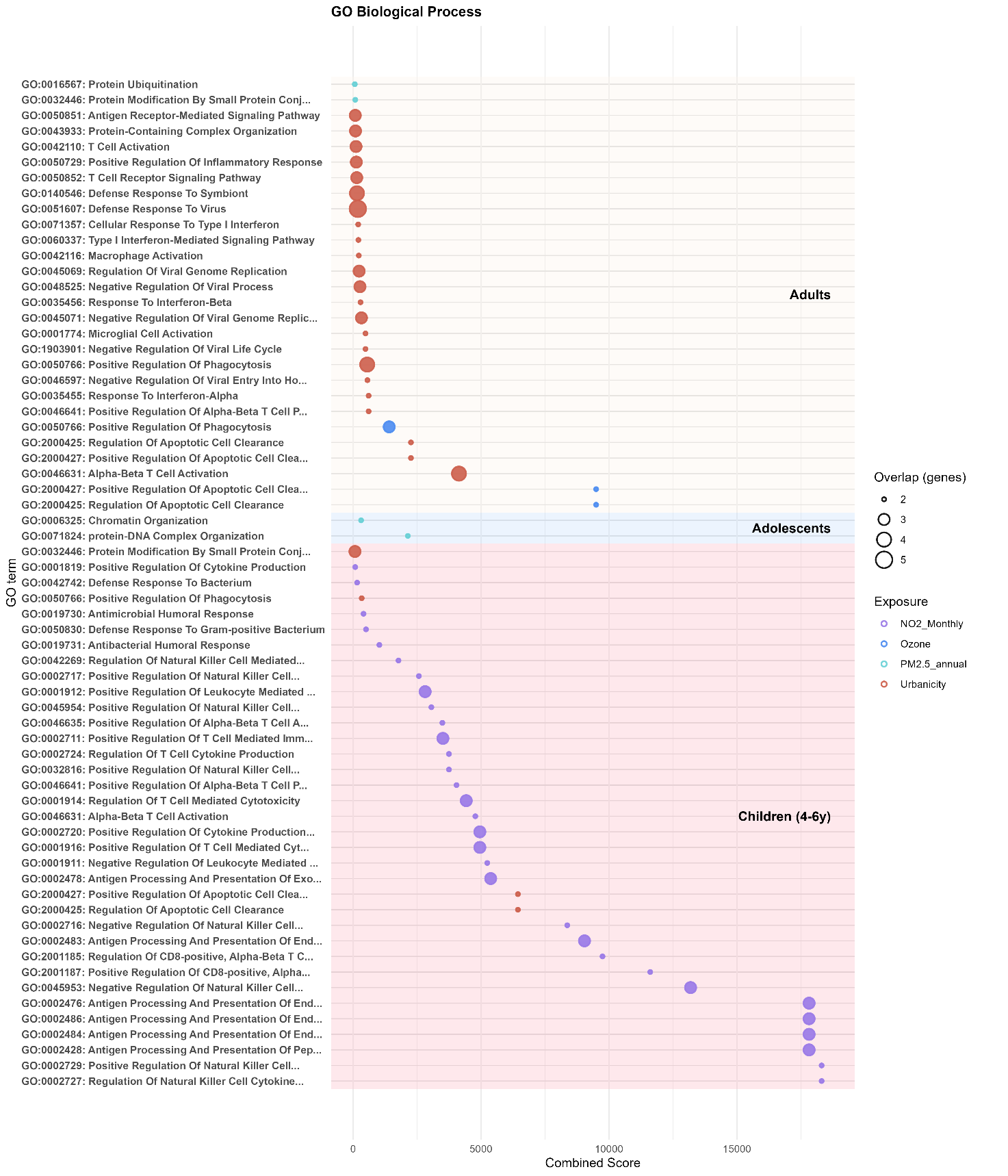

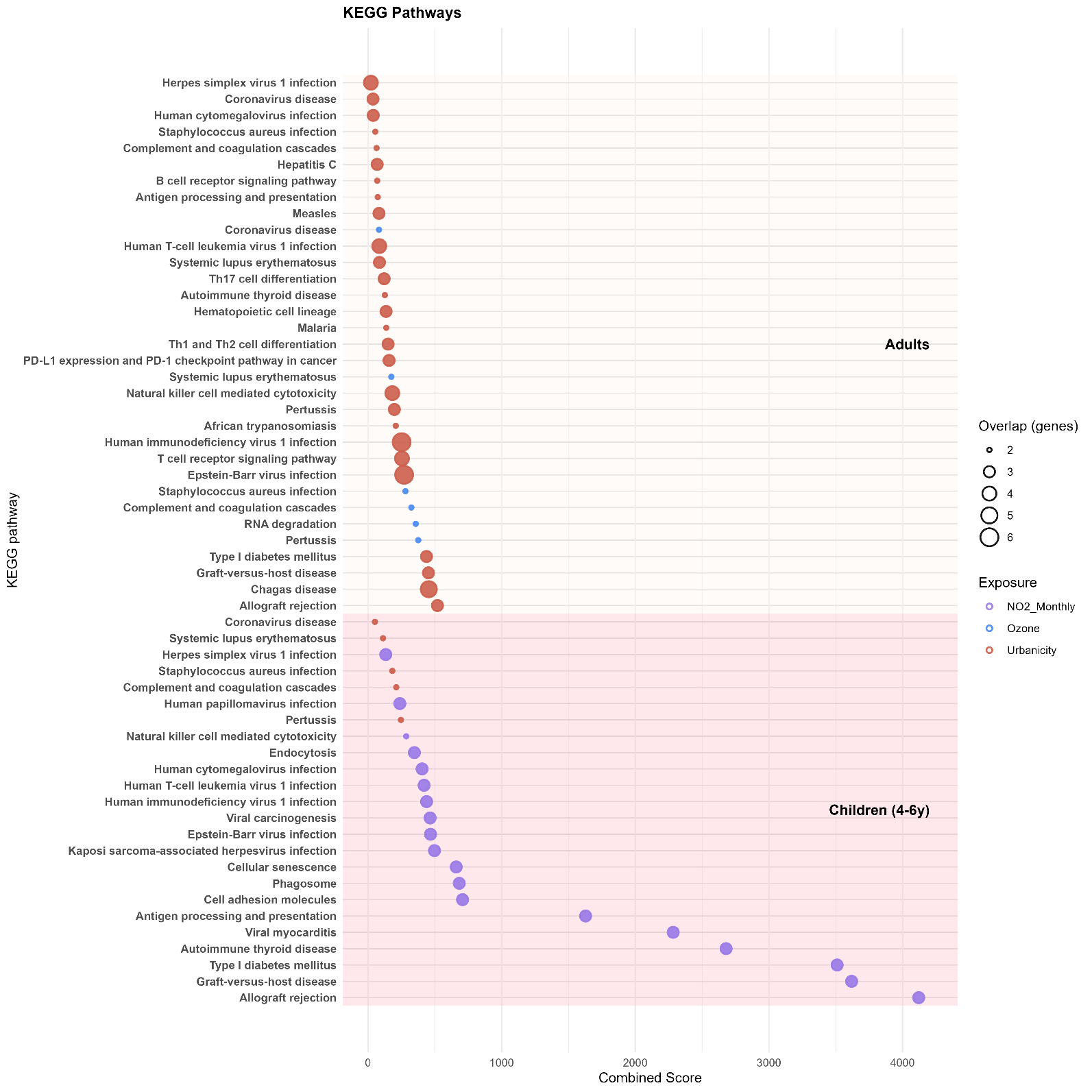


**Figure S6:** Results of KEGG pathway enrichment analysis performed with Enrichr (2). Analyses were based on genes mapped to CpGs included in significant DMRs, which were linked to transcripts via blood cis-eQTMs from the BIOS QTL browser prior to enrichment analysis. Only significant pathways (FDR < 0.05, including at least two genes per pathway) are presented and stratified by age group. Dot colors indicate the corresponding exposures, while dot size reflects the number of overlapping genes.

1. **References**

1. Sayers EW, Beck J, Bolton EE, Brister JR, Chan J, Comeau DC, et al. Database resources of the National Center for Biotechnology Information. Nucleic Acids Res. 2024;52(D1):D33-d43.

2. Kuleshov MV, Jones MR, Rouillard AD, Fernandez NF, Duan Q, Wang Z, et al. Enrichr: a comprehensive gene set enrichment analysis web server 2016 update. Nucleic Acids Research. 2016;44(W1):W90-W7.

3. Brunekreef B, Smit J, De Jongste J, Neijens H, Gerritsen J, Postma D, et al. The Prevention and Incidence of Asthma and Mite Allergy (PIAMA) birth cohort study: Design and first results. Pediatric Allergy and Immunology. 2002;13(s15):55-60.

4. Wijga AH, Kerkhof M, Gehring U, de Jongste JC, Postma DS, Aalberse RC, et al. Cohort profile: the prevention and incidence of asthma and mite allergy (PIAMA) birth cohort. Int J Epidemiol. 2014;43(2):527-35.

5. Xu C-J, Gruzieva O, Qi C, Esplugues A, Gehring U, Bergström A, et al. Shared DNA methylation signatures in childhood allergy: The MeDALL study. Journal of Allergy and Clinical Immunology. 2021;147(3):1031-40.

6. Wickman M, Kull I, Pershagen G, Nordvall SL. The BAMSE Project: presentation of a prospective longitudinal birth cohort study. Pediatric Allergy and Immunology. 2002;13(s15):11-3.

7. Chen YA, Lemire M, Choufani S, Butcher DT, Grafodatskaya D, Zanke BW, et al. Discovery of cross-reactive probes and polymorphic CpGs in the Illumina Infinium HumanMethylation450 microarray. Epigenetics. 2013;8(2):203-9.

8. Xu C-J, Bonder MJ, Söderhäll C, Bustamante M, Baïz N, Gehring U, et al. The emerging landscape of dynamic DNA methylation in early childhood. BMC Genomics. 2017;18(1).

9. Pidsley R, Y Wong CC, Volta M, Lunnon K, Mill J, Schalkwyk LC. A data-driven approach to preprocessing Illumina 450K methylation array data. BMC Genomics. 2013;14(1):293.

10. Reinius LE, Acevedo N, Joerink M, Pershagen G, Dahlén S-E, Greco D, et al. Differential DNA Methylation in Purified Human Blood Cells: Implications for Cell Lineage and Studies on Disease Susceptibility. PLoS ONE. 2012;7(7):e41361.

11. Houseman EA, Accomando WP, Koestler DC, Christensen BC, Marsit CJ, Nelson HH, et al. DNA methylation arrays as surrogate measures of cell mixture distribution. BMC Bioinformatics. 2012;13(1):86.

12. Heinrich J, Bolte G, Hölscher B, Douwes J, Lehmann I, Fahlbusch B, et al. Allergens and endotoxin on mothers' mattresses and total immunoglobulin E in cord blood of neonates. European Respiratory Journal. 2002;20(3):617-23.

13. Kilanowski A, Chen J, Everson T, Thiering E, Wilson R, Gladish N, et al. Methylation risk scores for childhood aeroallergen sensitization: results from the LISA birth cohort. Allergy. 2022;77(9):2803-17.

14. Piler P, Kandrnal V, Kukla L, Andrýsková L, Švancara J, Jarkovský J, et al. Cohort Profile: The European Longitudinal Study of Pregnancy and Childhood (ELSPAC) in the Czech Republic. International Journal of Epidemiology. 2016:dyw091.

15. Marečková K, Pačínková A, Klasnja A, Shin J, Andrýsková L, Stano-Kozubík K, et al. Epigenetic clock as a correlate of anxiety. Neuroimage Clin. 2020;28:102458.

16. Mareckova K, Mendes-Silva AP, Jáni M, Pacinkova A, Piler P, Gonçalves VF, et al. Mitochondrial DNA variants and their impact on epigenetic and biological aging in young adulthood. Translational Psychiatry. 2025;15(1).

17. Murata Y, Fujii A, Kanata S, Fujikawa S, Ikegame T, Nakachi Y, et al. Evaluation of the usefulness of saliva for DNA methylation analysis in cohort studies. Neuropsychopharmacology Reports. 2019;39(4):301-5.

18. Ligthart L, Van Beijsterveldt CEM, Kevenaar ST, De Zeeuw E, Van Bergen E, Bruins S, et al. The Netherlands Twin Register: Longitudinal Research Based on Twin and Twin-Family Designs. Twin Research and Human Genetics. 2019;22(6):623-36.

19. Willemsen G, De Geus EJC, Bartels M, Van Beijsterveldt CEMT, Brooks AI, Estourgie-Van Burk GF, et al. The Netherlands Twin Register Biobank: A Resource for Genetic Epidemiological Studies. Twin Research and Human Genetics. 2010;13(3):231-45.

20. Boomsma DI, Willemsen G, Sullivan PF, Heutink P, Meijer P, Sondervan D, et al. Genome-wide association of major depression: description of samples for the GAIN Major Depressive Disorder Study: NTR and NESDA biobank projects. European Journal of Human Genetics. 2008;16(3):335-42.

21. Van Dongen J, Nivard MG, Willemsen G, Hottenga J-J, Helmer Q, Dolan CV, et al. Genetic and environmental influences interact with age and sex in shaping the human methylome. Nature Communications. 2016;7(1):11115.

22. Bonder MJ, Luijk R, Zhernakova DV, Moed M, Deelen P, Vermaat M, et al. Disease variants alter transcription factor levels and methylation of their binding sites. Nature Genetics. 2017;49(1):131-8.

23. Van Iterson M, Tobi EW, Slieker RC, Den Hollander W, Luijk R, Slagboom PE, et al. MethylAid: visual and interactive quality control of large Illumina 450k datasets. Bioinformatics. 2014;30(23):3435-7.

24. Francioli LC, Menelaou, A., Pulit, S. L., van Dijk, F., Palamara, P. F., Elbers, C. C. Whole-genome sequence variation, population structure and demographic history of the Dutch population. Nature Genetics. 2014;46(8):818-25.

25. Chen Y-A, Lemire M, Choufani S, Butcher DT, Grafodatskaya D, Zanke BW, et al. Discovery of cross-reactive probes and polymorphic CpGs in the Illumina Infinium HumanMethylation450 microarray. Epigenetics. 2013;8(2):203-9.

26. Højsgaard S, Halekoh U, Yan J. The R Package geepack for Generalized Estimating Equations. Journal of Statistical Software. 2005;15(2):1 - 11.

27. Leitsalu L, Haller T, Esko T, Tammesoo M-L, Alavere H, Snieder H, et al. Cohort Profile: Estonian Biobank of the Estonian Genome Center, University of Tartu. International Journal of Epidemiology. 2015;44(4):1137-47.

28. Milani L, Alver M, Laur S, Reisberg S, Haller T, Aasmets O, et al. The Estonian Biobank’s journey from biobanking to personalized medicine. Nature Communications. 2025;16(1).

29. Lehne B, Drong AW, Loh M, Zhang W, Scott WR, Tan S-T, et al. A coherent approach for analysis of the Illumina HumanMethylation450 BeadChip improves data quality and performance in epigenome-wide association studies. Genome biology. 2015;16:1-12.

30. Downs SH, Schindler C, Liu LJS, Keidel D, Bayer-Oglesby L, Brutsche MH, et al. Reduced Exposure to PM<sub>10</sub>and Attenuated Age-Related Decline in Lung Function. New England Journal of Medicine. 2007;357(23):2338-47.

31. Adam M, Schikowski T, Carsin AE, Cai Y, Jacquemin B, Sanchez M, et al. Adult lung function and long-term air pollution exposure. ESCAPE: a multicentre cohort study and meta-analysis. European Respiratory Journal. 2015;45(1):38-50.

32. Imboden M, Wielscher M, Rezwan FI, Amaral AFS, Schaffner E, Jeong A, et al. Epigenome-wide association study of lung function level and its change. Eur Respir J. 2019;54(1).

33. Jeong A, Eze IC, Vienneau D, de Hoogh K, Keidel D, Rothe T, et al. Residential greenness-related DNA methylation changes. Environment International. 2022;158:160-4120.

34. Eze IC, Jeong A, Schaffner E, Rezwan FI, Ghantous A, Foraster M, et al. Genome-Wide DNA Methylation in Peripheral Blood and Long-Term Exposure to Source-Specific Transportation Noise and Air Pollution: The SAPALDIA Study. Environ Health Perspect. 2020;128(6):67003.

35. Aryee MJ, Jaffe AE, Corrada-Bravo H, Ladd-Acosta C, Feinberg AP, Hansen KD, et al. Minfi: a flexible and comprehensive Bioconductor package for the analysis of Infinium DNA methylation microarrays. Bioinformatics. 2014;30(10):1363-9.

36. Triche TJ, Weisenberger DJ, Van Den Berg D, Laird PW, Siegmund KD. Low-level processing of Illumina Infinium DNA Methylation BeadArrays. Nucleic Acids Research. 2013;41(7):e90-e.

37. Teschendorff AE, Marabita F, Lechner M, Bartlett T, Tegner J, Gomez-Cabrero D, et al. A beta-mixture quantile normalization method for correcting probe design bias in Illumina Infinium 450 k DNA methylation data. Bioinformatics. 2013;29(2):189-96.

38. Lehne B, Drong AW, Loh M, Zhang W, Scott WR, Tan S-T, et al. A coherent approach for analysis of the Illumina HumanMethylation450 BeadChip improves data quality and performance in epigenome-wide association studies. Genome Biology. 2015;16(1):37.

39. Zhou W, Laird PW, Shen H. Comprehensive characterization, annotation and innovative use of Infinium DNA methylation BeadChip probes. Nucleic Acids Research. 2016:gkw967.

40. Ritchie ME, Phipson B, Wu D, Hu Y, Law CW, Shi W, et al. limma powers differential expression analyses for RNA-sequencing and microarray studies. Nucleic Acids Research. 2015;43(7):e47-e.

41. Du P, Zhang X, Huang C-C, Jafari N, Kibbe WA, Hou L, et al. Comparison of Beta-value and M-value methods for quantifying methylation levels by microarray analysis. BMC Bioinformatics. 2010;11(1):587.
