# Supplementary material for: The impact of environmental exposures on DNA methylation in the EXPANSE project
Source: eBioMedicine. 2025 Dec 19;123:106084. doi: 10.1016/j.ebiom.2025.106084 (PMC12794040; doi:10.1016/j.ebiom.2025.106084)
Supplement: EXPANSE consortium [file mmc6.pdf]

First Names	Surnames	Affiliation
Apolline	Saucy	Barcelona Institute for Global Health (ISGlobal), Universitat Pompeu Fabra, CIBER Epidemiología y Salud
Cathryn	Tonne	Barcelona Institute for Global Health (ISGlobal), Universitat Pompeu Fabra, CIBER Epidemiología y Salud
Cristina	O‘Callaghan	Barcelona Institute for Global Health (ISGlobal), Universitat Pompeu Fabra, CIBER Epidemiología y Salud
Manolis	Kogevinas	Barcelona Institute for Global Health (ISGlobal), Universitat Pompeu Fabra, CIBER Epidemiología y Salud
Mark	Nieuwenhuijsen	Barcelona Institute for Global Health (ISGlobal), Universitat Pompeu Fabra, CIBER Epidemiología y Salud
Marta	Cirach	Barcelona Institute for Global Health (ISGlobal), Universitat Pompeu Fabra, CIBER Epidemiología y Salud
Natalia	Ortega	Barcelona Institute for Global Health (ISGlobal), Universitat Pompeu Fabra, CIBER Epidemiología y Salud
Payam	Dadvand	Barcelona Institute for Global Health (ISGlobal), Universitat Pompeu Fabra, CIBER Epidemiología y Salud
Sergio	Olmos	Barcelona Institute for Global Health (ISGlobal), Universitat Pompeu Fabra, CIBER Epidemiología y Salud
Erik	Melén	Department of Clinical Science and Education Södersjukhuset, Karolinska Institutet
Douglas	Walker	Department of Environmental Medicine and Public Health, Icahn School of Medicine at Mount Sinai, New York, NY
Joline	Beulens	Department of Epidemiology and Data Science, Amsterdam UMC, Vrije Universiteit, Amsterdam, the Netherlands
Maria Gabriela Matias	Pinho	Department of Epidemiology and Data Science, Amsterdam UMC, Vrije Universiteit, Amsterdam, the Netherlands
Joreintje	Mackenbach	Department of Epidemiology and Data Science, Amsterdam UMC, Vrije Universiteit, Amsterdam, the Netherlands
Licia	Iacoviello	1- Department of Epidemiology and Prevention, IRCCS Neuromed, Pozzilli. Italy<br>2- Research Center in Epidemiology and Preventive Medicine (EPIMED). Department of Medicine and Surgery. University of Insubria. Italy
Daniela	Porta	Department of Epidemiology, Lazio region Health Service / ASL Roma 1, Rome, Italy
Federica	Asta	Department of Epidemiology, Lazio region Health Service / ASL Roma 1, Rome, Italy
Martina	Culasso	Department of Epidemiology, Lazio region Health Service / ASL Roma 1, Rome, Italy
Massimo	Stafoggia	1- Department of Epidemiology, Lazio region Health Service / ASL Roma 1, Rome, Italy<br>2 - Institute of Environmental Medicine, Karolinska Institutet, Stockholm, Sweden
Ardine	de Wit	1-Department of Healthcare innovation and Evaluation, Juliuscentre for Healthsciences and Primary Care, University Medical Center Utrecht, Utrecht University, the Netherlands

		2- Centre for Nutrition, Prevention and Healthcare. National Institute of Public Health and the Environment, Bilthoven, the Netherlands
Tabea	Sonnenschein	1- Department of Healthcare innovation and Evaluation, Juliuscentre for Healthsciences and Primary Care, University Medical Center Utrecht, Utrecht University, the Netherlands<br>2- Department of Human Geography and Spatial Planning, Faculty of Geosciences, Utrecht University, the Netherlands<br>3- Department of Human Geography and Spatial Planning, Faculty of Geosciences, Utrecht University, the Netherlands
Simon	Scheider	Department of Human Geography and Spatial Planning, Faculty of Geosciences, Utrecht University, the Netherlands
Karin	Jongsma	Department of Medical Humanities, Julius Center for Health Sciences and Primary Care, University Medical Center Utrecht, Utrecht University, Utrecht, The Netherlands.
Annelien	Bredenoord	Department of Medical Humanities, University Medical Center Utrecht, Julius Center for Health Sciences and Primary Care, University Medical Center Utrecht, Utrecht University, Utrecht, The Netherlands
Caspar	Safarlou	Department of Medical Humanities, University Medical Center Utrecht, Julius Center for Health Sciences and Primary Care, University Medical Center Utrecht, Utrecht University, Utrecht, The Netherlands
Anna	Oudin	Department of Public Health and Clinical Medicine, Sustainable Health, Umeå University, Sweden.
Bertil	Forsberg	Department of Public Health and Clinical Medicine, Sustainable Health, Umeå University, Sweden.
David	Olsson	Department of Public Health and Clinical Medicine, Sustainable Health, Umeå University, Sweden.
Matteo	Bottai	Division of Biostatistics, Karolinska Institutet, Stockholm
Craig	Wheelock	Divsion of Physiological Chemistry2, Department of Medical Biochemistry and Biophysics, Karolinska Institute, Stockholm, Sweden
Jaanika	Kronberg	Estonian Genome Centre, Institute of Genomics, Riia 23b, Tartu, 51010, Estonia
Tarmo	Annilo	Estonian Genome Centre, Institute of Genomics, Riia 23b, Tartu, 51010, Estonia
Tõnu	Esko	Estonian Genome Centre, Institute of Genomics, Riia 23b, Tartu, 51010, Estonia
René	Luigies	Game Solutions Lab
Rob	Tieben	Game Solutions Lab
Anna	Carreras	GenomesForLife-GCAT Lab Group, Germans Trias i Pujol Research Institute
Rafael	de Cid	GenomesForLife-GCAT Lab Group, Germans Trias i Pujol Research Institute
Beatriz	Cortés	GenomesForLife-GCAT Lab Group, Germans Trias i Pujol Research Institute

Mireia	Obon	Bellvitge Biomedical Research Institute, Consortium for Biomedical Research in Epidemiology and Public Health and University of Barcelona, l'Hospitalet
Barbara	Bodinier	Imperial College London
Dragana	Vuckovic	Imperial College London
Dusan	Petrovic	Imperial College London
Jennifer	Quint	1- Imperial College London<br>2- NIHR Imperial Biomedical Research Centre
Marc	Chadeau-Hyam	Imperial College London
Matthew	Whitaker	Imperial College London
Paolo	Vineis	Imperial College London
Sarah	Filippi	Imperial College London
Sonia	Dagninio	Imperial College London
Thomas	Wright	Imperial College London
Verena	Zuber	Imperial College London
Anna	Bergström	1- Institute of Environmental Medicine, Karolinska Institutet, Stockholm, Sweden<br>2- Centre for Occupational and Environmental Medicine, Region Stockholm, Sweden
Göran	Pershagen	1- Institute of Environmental Medicine, Karolinska Institutet, Stockholm, Sweden<br>2- Centre for Occupational and Environmental Medicine, Region Stockholm, Sweden
Olena	Gruzieva	1- Institute of Environmental Medicine, Karolinska Institutet, Stockholm, Sweden<br>2- Centre for Occupational and Environmental Medicine, Region Stockholm, Sweden
Petter	Ljungman	1- Institute of Environmental Medicine, Karolinska Institutet, Stockholm, Sweden<br>2- Department of Cardiology, Danderyd Hospital, Stockholm, Sweden
Shizhen	He	Institute of Environmental Medicine, Karolinska Institutet, Stockholm, Sweden
Hynek	Pikhart	1- Institute of Epidemiology and Health Care, University College London, UK<br>2- RECETOX Centre, Faculty of Science, Masaryk University, Brno, Czech Republic
Martin	Bobak	1- Institute of Epidemiology and Health Care, University College London, UK<br>2- RECETOX Centre, Faculty of Science, Masaryk University, Brno, Czech Republic
Alexandra	Schneider	Institute of Epidemiology, Helmholtz Zentrum München, German Research Center for Evironmental Health, Neuherberg, Germany
Annette	Peters	1- Institute of Epidemiology, Helmholtz Zentrum München, German Research Center for Evironmental Health, Neuherberg, Germany<br>2- Ludwig-Maximilians-Universität, Munich, Germany<br>3- Harvard T.H. Chan School of Public Health, Boston, USA

Kathrin	Wolf	Institute of Epidemiology, Helmholtz Zentrum München, German Research Center for Evironmental Health, Neuherberg, Germany
Marie	Standl	Institute of Epidemiology, Helmholtz Zentrum München, German Research Center for Evironmental Health, Neuherberg, Germany
Regina	Pickford	Institute of Epidemiology, Helmholtz Zentrum München, German Research Center for Evironmental Health, Neuherberg, Germany
Susanne	Breitner	1- Institute of Epidemiology, Helmholtz Zentrum München, German Research Center for Evironmental Health, Neuherberg, Germany<br>2- IBE-Chair of Epidemiology, Ludwig-Maximilians-Universität München, Munich, Germany.
Tianyu	Zhao	Institute of Epidemiology, Helmholtz Zentrum München, German Research Center for Evironmental Health, Neuherberg, Germany
Melanie	Waldenberger	1- Institute of Epidemiology, Helmholtz Zentrum München, German Research Center for Evironmental Health, Neuherberg, Germany<br>2- Institute of Epidemiology, Helmholtz Zentrum München, German Research Center for Environmental Health, D-85764, Neuherberg, Germany<br>3- German Center for Cardiovascular Research (DZHK), Partner Site Munich Heart Alliance, Munich, Germany
Augustin	Scalbert	International Agency for Research on Cancer, Nutrition and Metabolism Section, Lyon
Inge	Huybrechts	International Agency for Research on Cancer, Nutrition and Metabolism Section, Lyon
Marc	Gunter	International Agency for Research on Cancer, Nutrition and Metabolism Section, Lyon
Pekka	Keski-Rahkonen	International Agency for Research on Cancer, Nutrition and Metabolism Section, Lyon
Reza	Salek	International Agency for Research on Cancer, Nutrition and Metabolism Section, Lyon
Jana	Klánová	RECETOX Centre, Faculty of Science, Masaryk University, Brno, Czech Republic
Lenka	Andrýsková	RECETOX Centre, Faculty of Science, Masaryk University, Brno, Czech Republic
Ondřej	Mikeš	RECETOX Centre, Faculty of Science, Masaryk University, Brno, Czech Republic
Pavel	Čupr	RECETOX Centre, Faculty of Science, Masaryk University, Brno, Czech Republic
Pavel	Piler	RECETOX Centre, Faculty of Science, Masaryk University, Brno, Czech Republic
Richard	Hůlek	RECETOX Centre, Faculty of Science, Masaryk University, Brno, Czech Republic
Zdenka	Dudová	Institute of Computer Science, Masaryk University, Brno, Czech Republic
Nicole	Janssen	RIVM, Centre for Sustainability, Environment and Health

Alonso	Bussalleu	1- Swiss Tropical and Public Health Institute, Basel, Switzerland<br>2- University of Basel, Switzerland
Ayoung	Jeong	1- Swiss Tropical and Public Health Institute, Basel, Switzerland<br>2- University of Basel, Switzerland
Benjamin	Flückiger	1- Swiss Tropical and Public Health Institute, Basel, Switzerland<br>2- University of Basel, Switzerland
Danielle	Vienneau	1- Swiss Tropical and Public Health Institute, Basel, Switzerland<br>2- University of Basel, Switzerland
Dirk	Keidel	1- Swiss Tropical and Public Health Institute, Basel, Switzerland<br>2- University of Basel, Switzerland
Emmanuel	Schaffner	1- Swiss Tropical and Public Health Institute, Basel, Switzerland<br>2- University of Basel, Switzerland
Gianfranco	Lovison	1- Swiss Tropical and Public Health Institute, Basel, Switzerland<br>2- University of Basel, Switzerland<br>3- Università degli Studi di Palermo
Ikenna	Eze	1- Swiss Tropical and Public Health Institute, Basel, Switzerland<br>2- University of Basel, Switzerland
Kees	de Hoogh	1- Swiss Tropical and Public Health Institute, Basel, Switzerland<br>2- University of Basel, Switzerland
Marek	Kwiatkowski	1- Swiss Tropical and Public Health Institute, Basel, Switzerland<br>2- University of Basel, Switzerland
Medea	Imboden	1- Swiss Tropical and Public Health Institute, Basel, Switzerland<br>2- University of Basel, Switzerland
Nicole	Probst-Hensch	1- Swiss Tropical and Public Health Institute, Basel, Switzerland<br>2- University of Basel, Switzerland
Cyrille	Delpierre	UMR 1027, Université de Toulouse, UPS, Inserm, Toulouse, France
Marine	Maurel	UMR 1027, Université de Toulouse, UPS, Inserm, Toulouse, France
Michelle	Kelly-Irving	UMR 1027, Université de Toulouse, UPS, Inserm, Toulouse, France
Raphaële	Castagné	UMR 1027, Université de Toulouse, UPS, Inserm, Toulouse, France
Benoit	Lepage	UMR 1027, Université de Toulouse, UPS, Inserm, Toulouse, France; Department of Epidemiology, University Hospital of Toulouse, Toulouse, France.
Evangelia	Samoli	National and Kapodisitrian University of Athens
Klea	Katsouyanni	1- National and Kapodisitrian University of Athens

		2- Imperial College, UK
Konstantina	Dimakopoulou	National and Kapodisitrian University of Athens
Sophia	Rodopoulou	National and Kapodisitrian University of Athens
Maria Iosifina	Kasdagli	National and Kapodisitrian University of Athens
Dimitris	Evangelopoulos	part of NKUA team, affiliated at Imperial College, UK
Anke	Huss	Utrecht University
Esmeralda	Krop	Utrecht University
Gerard	Hoek	Utrecht University
Jelle	Vlaanderen	Utrecht University
Jingxian	You	Utrecht University
Jules	Kerckhoffs	Utrecht University
Kalliopi	Kyriakou	Utrecht University
Lützen	Portengen	Utrecht University
Martje	Ebberink	Utrecht University
Roel	Vermeulen	1- Utrecht University<br>2- Julius Center for Health Sciences and Primary Care, University Medical Center Utrecht<br>3- Imperial College London
Ulrike	Gehring	Utrecht University
You-chen	Shen	Utrecht University
Zhendong	Yuan	Utrecht University
Jeroen	Lakerveld	Department of Epidemiology and Data Science, Amsterdam UMC, Vrije Universiteit, Amsterdam, the Netherlands
Alessandro	Gialluisi	Department of Epidemiology and Prevention, IRCCS Neuromed, Pozzilli. Italy
Gary	Miller	Columbia University, New York, NY, USA
Jurriaan	van Rijswijk	Game Solutions Lab
Simona	Costanzo	Department of Epidemiology and Prevention, IRCCS Neuromed, Pozzilli. Italy
Werner	Rutten	Game Solutions Lab
Marta	Mańczuk	Maria Sklodowska-Curie National Research Instiute of Oncology
Paweł	Koczkodaj	Maria Sklodowska-Curie National Research Instiute of Oncology
Agata	Ciuba	Maria Sklodowska-Curie National Research Instiute of Oncology
Kinga	Polańska	Nofer Institute of Occupational Medicine
Wojciech	Hanke	Nofer Institute of Occupational Medicine
Agnieszka	Jankowska	Nofer Institute of Occupational Medicine
Agnieszka	Pac	Jagiellonian University Medical College, Chair of Epidemiology and Environmental Medicine
Elzbieta	Sochacka-Tatara	Jagiellonian University Medical College, Chair of Epidemiology and Environmental Medicine
Renata	Majewska	Jagiellonian University Medical College, Chair of Epidemiology and Environmental Medicine
